# Map-based cloning of the susceptibility locus conditioning *bs6* resistance in pepper (*Capsicum annuum* L.) encoding mutant alleles of pseudokinase proteins

**DOI:** 10.1007/s00122-026-05407-2

**Published:** 2026-10-20

**Authors:** Zoltan Toth, Zoltan Gabor Toth, Peter Kalo, Gyorgy B. Kiss, Zoltan Szabo

**Affiliations:** 1 Institute of Genetics and Biotechnology, Hungarian University of Agriculture and Life Sciences, Szent-Györgyi A. u. 4., Gödöllő, 2100 Hungary; 2 AMBIS Biotechnology Research and Development Ltd., Budapest, Hungary; 3 Institute of Plant Biology, HUN-REN Biological Research Centre, Szeged, Hungary

## Abstract

***Key message*:**

The *bs6* resistance gene against bacterial spot was identified by map-based cloning in pepper (C. *annuum* L.).

**Abstract:**

The recessive *bs6*^*61*^ locus of pepper *Capsicum annuum* L. var. 61R (Ca61R) conditions a non-hypersensitive resistance trait, characterized by a slightly swollen, pale green, photosynthetically active leaf tissue, following *Xanthomonas euvesicatoria* Race 6 (XeR6) infection. In contrast, *C. annuum* cv. Feherozon (CaFo) is susceptible to XeR6. Map-based cloning of the *bs6*^*61*^ locus revealed that the *bs6*^*61*^ locus encodes three predicted proteins, pbs6^61^npk, pbs6^61^n, pbs6^61^pk in Ca61R which are the counterpart of the three wild type predicted proteins, pBs6^Fo^NPK, pBs6^Fo^N and pBs6^Fo^PK in CaFo, respectively. Sequence analysis revealed that the pBs6FoNPK protein showed 35% identity and 53% similarity to the ZRK4 pseudokinase (pAtZRK4) identified in *Arabidopsis thaliana*, which possesses N-terminal and C-terminal (pseudokinase) domains. The pBs6^Fo^N and pBs6^Fo^PK proteins resemble the N-terminal and C-terminal domains of the pBs6FoNPK protein, respectively, thus, they also contain pseudokinase domains/sequences. In Ca61R, pbs6^61^npk and pbs6^61^n are truncated counterparts of pBs6^Fo^NPK and pBs6^Fo^N, respectively, as a consequence of a single-nucleotide insertion that causes frameshifts and premature stop codons, whereas pbs6^61^pk differs from pBs6^Fo^PK by a single amino acid, a serine to alanine substitution at position 133. These three proteins are considered functionless. The ORF of pBs6^Fo^N, pBs6^Fo^PK and pbs6^61^n, pbs6^61^pk, respectively, are encoded by a single transcript in both parental plants. For this reason the translation of pBs6^Fo^PK was ambiguous, however, the expression of pBs6^Fo^N-dsRed fusion protein demonstrated that pBs6^Fo^PK in fact can be translated. The three wild type proteins (pBs6^Fo^NPK, pBs6^Fo^N and pBs6^Fo^PK) were independently responsible for ETS (effector-triggered susceptibility), because all three genes had to be in mutant configuration to manifest the resistant phenotype, in addition, any one of the three was able to reverse the resistant phenotype to susceptibility. Functional analysis using virus-induced gene silencing and transient genetic complementation demonstrated that the three CaFo proteins contribute redundantly to XeR6 susceptibility. Silencing either Bs6^Fo^NPK, Bs6^Fo^N and Bs6^Fo^PK alone did not alter susceptibility, whereas simultaneous silencing of these genes conferred resistance. Conversely, the individual expression of pBs6^Fo^NPK, pBs6^Fo^N, and pBs6^Fo^PK, in the resistant Ca61R background each restored susceptibility. These results demonstrate that the three Bs6 proteins act redundantly to promote XeR6 susceptibility and that loss of function of all three corresponding proteins was required for the recessive resistance phenotype conferred by *bs6*^*61*^.

**Supplementary Information:**

The online version contains supplementary material available at https://doi.org/10.1007/s00122-026-05407-2.

## Introduction

The persistent fight against plant pathogens does not always lead to satisfactory results, since dominant resistance genes (mainly used in breeding programs) introgressed into cultivated crops only provide temporary protection, because pathogens will sooner or later break through the resistance and become virulent (Kousik and Ritchie 1998; Romero and Ritchie 2004). To avoid these frustrating manifestations, plant breeders are striving to develop durable resistance. One promising opportunity to achieve this goal is to identify and apply mutated susceptibility genes. Such recessive resistance can be characterized by the loss-of-function genetic change in susceptibility genes which might be appropriate and satisfactory with no deleterious effect on plant growth, development and yield (Jørgensen 1992; Jones et al. 2002; Wang et al. 2014; Kusch and Panstruga 2017; Oliva et al. 2019; Garcia-Ruiz et al. 2021). However, there are examples of disadvantageous mutated recessive susceptibility genes which display a reduction in fitness, abnormal development and morphology, dwarfism and yield loss (Yu et al. 1998; Clough et al. 2000; Jurkowski et al. 2004).

Plant-pathogen interactions can be manifested in three main outcomes: resistance, tolerance or disease. During resistance (incompatible interactions), the pathogen may initially penetrate and colonize host tissues, but their growth and spread are subsequently restricted by the plant immune system preventing disease development. Resistance can be mediated by dominant or recessive resistance genes (R genes) and is achieved by the coordinated activation of innate immune responses, including pattern triggered immunity (PTI), effector triggered immunity (ETI) and the systemic acquired resistance (SAR) pathways. These immune mechanisms have been extensively studied and are comprehensively reviewed elsewhere and therefore they are not discussed here (Jones and Dangl 2006; Ngou et al. 2022).

On the other hand, tolerance and susceptible interactions have received considerably less attention in the scientific literature (Gorshkov and Tsers 2022; Koseoglou et al. 2021). Plant tolerance is defined as the ability of a host to maintain growth and reproductive fitness despite substantial pathogen colonization (Kover and Schaal 2002; Tang et al. 2025). Unlike resistance, tolerance does not limit pathogen multiplication but instead mitigates its detrimental effects on plant performance. Because the molecular mechanisms underlying tolerance remain poorly understood, its exploitation in crop breeding is currently limited. By contrast, susceptibility results from the inability of the host to effectively restrict pathogen infection and proliferation, ultimately leading to disease.

Understanding the genetic, molecular and physiological events leading to susceptibility interactions, and consequently disease development, is essential because achievements in this field can give clues for breeding for crops with durable resistance, thereby improving agricultural production. Molecular dissection of each and every recessive resistance gene will broaden our knowledge and provide breeding solutions to serve environmentally friendly and sustainable modern agriculture.

The causal agent of bacterial spot (BS) in pepper and tomato is *Xanthomonas euvesicatoria* (previously *X. campestris pv. vesicatoria*; Jones et al. 2004). Bacterial spot disease significantly damages pepper and tomato production around the world especially where high temperature and humidity occurs which are ideal conditions for disease development. The behavior, transmission mechanism, genes controlling infection and other aspects of the pathogen are described expansively in many scientific publications (Stall et al. 2009; Potnis et al. 2015; Osdaghi et al. 2021; Poudel et al. 2025). To this end, several loci conditioning resistance to *X. euvesicatoria* infection are known in pepper (*C. annuum* L.) and were used in breeding program to assist high pepper yield (Cook and Guevara 1984; Kim and Hartman, 1985; Hibberd et al. 1987; Ronald and Staskawicz 1988; Tai et al. 1999; Römer et al. 2007; Wai et al. 2015; Jones et al. 2002). These resistance loci controlling bacterial spot disease can be either dominant, e.g. *Bs1*, *Bs2*, *Bs3* and *Bs7* genes or recessive, e.g. *bs5*, *bs6* and *bs8* genes (Stall et al. 2009; Potnis et al. 2012; Jones et al. 2002; Sharma et al. 2022; Szabó et al. 2023; Poudel et al. 2025). These genes have been pyramided into pepper breeding lines, ECW12346 (Vallejos et al. 2010), carrying *Bs1*, *Bs2*, *Bs3* together with the two recessive resistance determinants, *bs5* and *bs6*. The recessive resistance genes *bs5*, *bs6*, *bs8* have been pyramided into ECW568 (Poudel et al. 2025). Four of these resistant genes (*Bs1*, *Bs2*, *Bs3* and *bs5*) have been identified and cloned previously (Tai et al. 1999; Römer et al. 2007; Szabó et al. 2023). In this study we are contributing to the above-mentioned noble goal by identification of the genes conditioning the recessive susceptibility gene *bs6* of pepper (*C. annuum* L.) which could contribute to the breeding of pepper and relative crop plants like tomato (*Solanum lycopersicum* L.) and other species.

## Materials and methods

### Bacterial strains and plant material

For pathogenicity tests, the following *Xanthomonas* species were used:* X. euvesicatoria* Race 6 (XeR6). XeR6 carried no known *avr* genes (Pernezny et al. 1999); *Xanthomonas gardneri* (Xg) (Šutic 1957); *Xanthomonas perforans* (Xp) (Jones et al. 2000). The following plant materials were used in this study: (i) XeR6 susceptible plants (Xe^S^): 1. *C. annuum* L. cv. Feherozon (CaFo), a commercial Hungarian „Sweet Bell pepper” cultivar (https://www.kertimag.hu/bogyosok/feherozon.html); 2. *C. annuum* cv. CM334, a hot pepper Mexican landrace Criollo de Morelos 334 (https://plants.ensembl.org/Capsicum_annuum/Info/Annotation/); 3. *C. annuum* cv. MicropepRed, kindly provided by Byoung-Cheorl Kang, Seoul National University, South Korea; (ii) XeR6 resistant plants (Xe^R^): 1. *C. annuum* cv. Early CalWonder 60R (ECW60R), kindly provided by Jeffrey B. Jones, University of Florida, Gainesville, Florida, USA (Vallejos et al. 2010); 2. *C. annuum* var. 61R (Ca61R), a single seed plant from accession no. PI 271322 (kindly provided by Jeffrey B. Jones, see Sect. “Results”) carrying the *bs6* allele; 3. *C. annuum* cv. Perennial (CaPe). Progeny plant individuals are numbered with a P prefix.

### Plant growth conditions and crossing

Before use, pepper seeds were kept at 4 °C for one to two weeks for vernalization, then sown, germinated and grown under greenhouse conditions in 4-inch pots containing “Florimo®” general flower soil (http://www.florimo.hu/termekeink/viragfoldek/) at 25 °C for 16/8 h light/dark period. To generate F1 hybrids, maternal flowers were emasculated and manually crossed with pollen from the paternal parent. F2 and F3 seeds were collected from self-pollinated plants.

### *Xanthomonas* infection and evaluation of symptoms

**Fig. 1:**
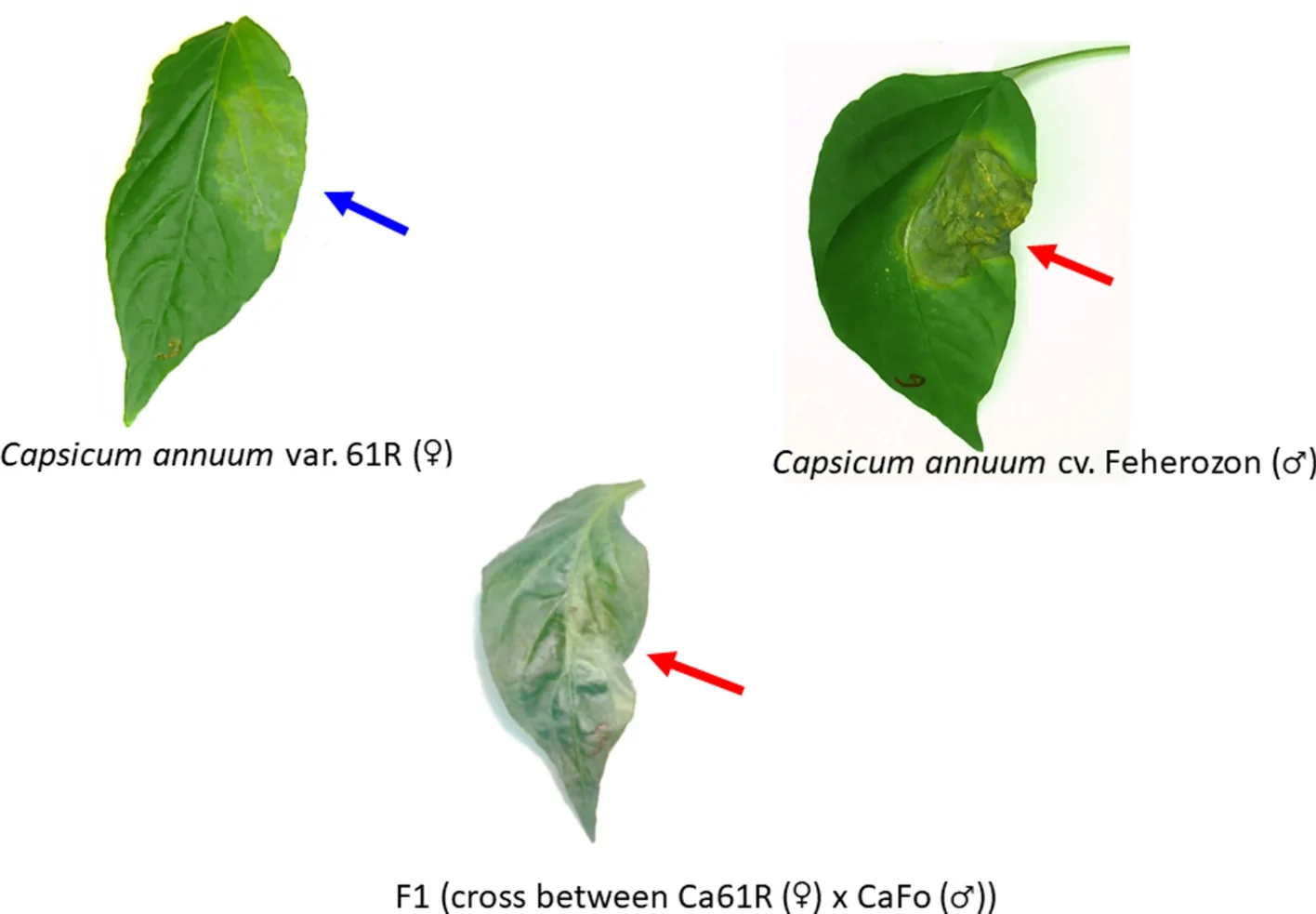
Disease symptoms of leaves following infection with *X. euvesicatoria* R6 strain. Leaf phenotype of parents (*C. annuum* var. 61R, *C. annuum* cv. Feherozon) and F1 (Ca61R x CaFo) hybrid plants 6 days post inoculation with XeR6. Blue arrows show resistant, while red arrows show the susceptible leaf phenotype. The inoculation procedure and phenotypes of the leaves are described in Sect. “Materials and methods”

For the determination of the *Xanthomonas* resistant and susceptible phenotypes, leaf infections with XeR6, Xg, and Xp bacteria were performed. Bacteria were grown in Yeast extract-Dextrose-Calcium Carbonate medium (YDC) at 28 °C until stationary phase, pelleted by centrifugation and suspended in sterile tap water at a concentration of 10^5^ CFU/mL. Inoculation was done with 1–2 × 10^4^ XeR6 bacteria per leaf. The abaxial side of young leaves (8-leaf stage) was infiltrated with a syringe fitted with a rubber tube. Infected plants were grown under greenhouse conditions as described above. The evaluation of the disease symptoms was as follows: XeR6^R^ plants showed slight discoloration, healthy, but swollen tissue (Fig. 1) following 6 days post-inoculation (DPI) and in later stage as well. Abscission of the leaves was not observed. Xe^S^, Xg^S^, Xp^S^ plants exhibited yellowing, water-soaking after 4–6 DPI, complete necrosis after 6–12 DPI and abscission of the infected leaves after two weeks.

### DNA extraction, PCR amplification and electrophoresis of amplicons

Total DNA was isolated from fresh leaves (100 mg/sample) using a Zenogene DNA purification kit (Zenon Bio, Szeged, Hungary) according to the manufacturer’s instructions. The PCR reactions were carried out in 12 µL reaction mixtures containing 1.2 µL of 10× PCR buffer, 0.3 U Taq polymerase, 0.2 mM dNTP, 0.5 µM of each primer, and 10 ng template DNA. Thermal cycling conditions consisted of one cycle of initial denaturation for 4 min at 94 °C, followed by 30 cycles at 94 °C for 60 s, 58 °C for 60 s, 72 °C for 60 s, with a final extension step of 7 min at 72 °C. Amplified products were separated by agarose gel electrophoresis on a 1.0% (w/v) agarose gel stained with 0.5 mg/L ethidium bromide solution and then visualized and photographed on a UV transilluminator. Fragments, which were not polymorphic on agarose gel were subjected to SSCP (Single Strand Conformation Polymorphism; Orita et al. 1989) analysis. Buffer, gels and staining were prepared and performed by standard procedures. Cleaved Amplified Polymorphic Sequence (CAPS) markers (Glazebrook et al. 1998) were resolved on agarose gels after restriction enzyme digestion.

### Marker development

Published genetic markers (Nagy et al. 2007) and newly developed markers were used for mapping the *bs6*^*61*^ locus. New markers were generated based on DNA sequence comparison of CaECW, CaPe and sequences available in different databases (NCBI (https://www.ncbi.nlm.nih.gov/), Sol Genomics Network (https://solgenomics.net/ and sequence reads SRX30438917 of Ca61R were used) were compared and polymorphic sequences were targeted, and genetic markers were developed by designing appropriate primer pairs. Primers were designed by Primer3 version 4.0 software (Untergasser et al. 2012). Synthesized primers were ordered from Thermo Fisher Scientific Inc. Genetic markers used in this study are listed in Table S1.

### Genetic mapping

The genotypes of the molecular markers were resolved after PCR amplification according to length polymorphism (running agarose or polyacrylamide gels) and SSCP. Polymorphisms were converted to genotypes according to the parental pattern. Genetic mapping was done by colormapping as described by Kiss et al. (1998). Genotypes were represented by numbers (in colored background) as follows: 1 (yellow), paternal homozygotes; 2 (dark green), heterozygotes; 3 (purple), maternal homozygotes; 5 (light green), non-maternal homozygotes. XeR6 phenotypes (Xe^R^/Xe^S^) were converted to genotypes as follows: S in pale green background corresponded to susceptible, while R in purple background, to resistant phenotype, respectively.

### Transformation of *Agrobacterium tumefaciens*

Competent cells of *A. tumefaciens* strains (stored at −80℃) were thawed on ice and 5 μL of recombinant plasmids were added, then kept on ice for 5 min. The mixture was fast frozen in liquid nitrogen for 5 min, followed by an incubation at 37℃ for 12 min. The cells were kept on ice for 5 min, and 900 μL of fresh Yeast Extract Beef (YEB) liquid medium was added. The culture was incubated in a shaker for 2–4 h at 28 °C and centrifuged for 30 s at 2000 rpm. The supernatant was discarded, and the cells were resuspended in 100 μL of fresh YEB. The suspension was plated on YEB agar containing rifampicin (25 mg/L) and kanamycin (50 mg/L), and the plates were incubated in the dark at 28 °C for 2–3 days.

### Virus-induced gene silencing (VIGS)

For virus-induced gene silencing (VIGS), gene-specific and gene non-specific DNA regions were selected to maximize silencing specificity for the targeted genes, *Bs6*^*Fo*^*NPK*, and *Bs6*^*Fo*^*N-Bs6*^*Fo*^*PK*, respectively. These specific and non-specific regions were identified by the online *VIGS tools* provided by the Sol Genomics Network (SGN; https://solgenomics.net/). The target region for the specific sequences recognized only their own gene. The target region for the non-specific sequences recognized the transcripts of both genes. The names of the specific and non-specific sequence fragments for gene *Bs6*^*Fo*^*NPK* were: fragment fr_Bs6^Fo^NPK_VSp and fr_Bs6^Fo^NPK_VNs; for gene *Bs6*^*Fo*^*N-Bs6*^*Fo*^*PK*: fragments fr_Bs6^Fo^n_VSp, fr_Bs6^Fo^PK_VSp and fr_Bs6^Fo^PK_VNs. The sequences of these fragments are listed in Table S2. The DNA corresponding to these fragment sequences were amplified using *Bs6*^*Fo*^*NPK*, and *Bs6*^*Fo*^*N-Bs6*^*Fo*^*PK* specific primers (listed in Table S1) and CaFo DNA as template. After amplification and cleavage with EcoRI and BamHI restriction enzymes the amplicons were cloned into pTRV-RNA2 (https://www.addgene.org/). VIGS was performed using the pTRV-RNA1 and pTRV-RNA2 vectors, which were transformed into *A. tumefaciens* strain C58. To confirm successful systemic silencing efficiency via photobleaching symptoms a phytoene desaturase (PDS) gene construct was used as a positive control. The resulting TRV-based constructs were delivered into *Nicotiana benthamiana* plants by *A. tumefaciens*–mediated infiltration. Following systemic spread of the recombinant virus in infiltrated *N. benthamiana*, virus-containing inoculum was subsequently transferred to CaFo seedlings at the two-cotyledon stage. After agroinfiltration and the subsequent passage, plants were maintained in a controlled-environment growth chamber at 20–22 °C under long-day photoperiod conditions (16 h light/8 h dark). Inoculation with XeR6 bacteria was performed on the 5th–6th fully expanded leaf stage. Symptoms were observed 6–10 days post-inoculation (DPI). The silencing efficiency was checked with qPCR (Lightcycler 96, Roche) on leaf samples harvested from VIGS-treated plants (non-specific dual-silencing constructs). Expression analysis was done for *Bs6*^*Fo*^*NPK*, *Bs6*^*Fo*^*N* and *Bs6*^*Fo*^*PK* with GAPDH2 as a control (Zhan et al. 

2025) with gene specific primers (Table S1) using qPCRBIO SyGreen qPCR kit (PCR Biosystems, London, UK). qPCR products were tested on 1.5% agarose gel electrophoresis.

### *Agrobacterium* culture and transient expression

Single positive colonies of *Agrobacterium* strains harbouring constructs and P14 RNA silencing suppressor were inoculated in 1 mL ABLO liquid medium (10 mM MES, 100 mg/L kanamycin, 25 mg/L rifampicin, 200 μM acetosyringone) and cultured for 18–24 h in a shaker at 28℃, 200 rpm. 500 μL *Agrobacterium* cells were transferred into 10 mL fresh LB liquid medium supplemented with GV3101 and construct-specific antibiotics and cultured overnight at 28℃, 200 rpm. An aliquot of 2 mL *Agrobacterium* cells were pelleted by centrifugation (3500 rpm, 15 min) and re-suspended with 1000 μL AVLO medium (10 mM MES, 200 μM acetosyringone, 10 mM MgCl_2_). The re-suspended cells were diluted to OD600 = 0.4 with infiltration solution (AVLO medium). After resting for 3 h, the *Agrobacterium* solution was mixed in a 1:1 ratio with the strain carrying the P14 RNA silencing suppressor. The bacterial suspensions were injected using a needleless syringe into XeR6 resistant *C. annuum* and CaFo seedlings grown at 25℃ with 60% humidity and a 16-h light and 8-h dark photoperiod in a growth chamber. The top three leaves of three independent plantlets (approximately 6–8 weeks old) were infiltrated with each vector or vector combination. This process was repeated in three replicates. The plants expressing each construct were kept in growth chambers at 22℃ with 16 h illumination/8 h dark conditions. For various studies (subcellular localization, infection and evaluation of disease symptoms) samples were collected at various time periods post agroinfiltration. The constructs used for transient gene expression were pCAMBIA0390-*Bs6*^*Fo*^*NPK*, pCAMBIA0390-*Bs6*^*Fo*^*N*, pCAMBIA0390-*bs6*^*61*^*-Bs6*^*Fo*^*PK* and pCAMBIA0390-*Bs6*^*Fo*^*-PsK*^*751*^. The primers used to amplify the DNA from CaFo and P1023F3 plants are listed in Table S1. The unrelated pseudokinase gene was *PsK*^*751*^ (VYZY01010751.1:168822-169922* C. annuum* cv. ECW scaffold68364) used as a negative control. After amplification the fragments were first cloned into pJET1.2 vector (Thermo Fisher Scientific, US) following cleavage with SalI and ApaI restriction enzymes and then cloned into pCAMBIA0390 vector (https://www.addgene.org/). The constructs contained the native promoter of the gene. The chimeric gene was subsequently assembled using overlap-extension PCR (Nelson and Fitch 2011). The resulting plasmids were introduced from *E. coli* into *A. tumefaciens* strain GV3101 by freeze–thaw method and were infiltrated into leaves of 4-week-old Ca61R leaves along with an *E. coli* strain expressing P14 RNA silencing suppressor. Bacterial inoculation was performed concurrently with infection, using 1–2 × 10^4^ XeR6 bacteria per leaf. After 7 days the disease symptoms were evaluated.

## Results

### Selecting the parental plants for the genetic mapping of the *bs6*^*61*^ genetic determinant in pepper

To identify the *bs6*^*61*^ locus, a map-based cloning approach was employed. To select an appropriate single plant individual for genetic mapping of the *bs6*^*61*^ locus, pathogenicity and allelism tests were carried out on 200 plants grown from seeds of accession PI 271322 from which the *bs6*^*61*^ genetic determinant originally derived (Vallejos et al. 2010). After sowing, leaves were inoculated with *X. euvesicatoria* Race 6 (XeR6) and symptoms were visually assessed six days post-inoculation (DPI). One plant displaying non-hypersensitive XeR6 resistant (XeR6^R^) response was selected and denoted *C. annuum* var. 61R (Ca61R*,* Fig. 1). To ascertain whether Ca61R plant carried the *bs6* allele, an allelism test was performed using ECW60R as reference line in which XeR6^R^ resistance is conditioned by a single, homozygous recessive locus (*bs6*^*60R*^; Vallejos et al. 2010). For this purpose, Ca61R was emasculated and used as the maternal parent to cross with ECW60R as paternal parent. F1 seeds were germinated, and the resulting plants were inoculated with XeR6 and evaluated for symptoms. All 31 F1 progeny were XeR6^R^ demonstrating that the *bs6*^*61*^ locus controlling XeR6^R^ in Ca61R is allelic to *bs6*^*60R*^ in ECW60R*.* As a paternal parent, CaFo was selected displaying XeR6 susceptible phenotype (XeR6^S^; Fig. 1).

### Genetic mapping of the *bs6*^*61*^* locus*

To elucidate the genetic basis and chromosomal location of the recessive *bs6*^*61*^ resistance locus conferring resistance to a broad spectrum of *X. euvesicatoria* (*Xe*) races, we performed map-based cloning using an F2 population derived from a cross between the resistant Ca61R and susceptible CaFo pepper lines. To clone DNA sequence conditioning *bs6*^*61*^ locus mediated resistance, an F2 segregation population was generated by self-mating F1 hybrids derived from the cross Ca61R (♀) x *CaFo* (♂) plants. Leaves of F1 hybrids and parental plants were tested for disease symptoms 6 days post-inoculation (DPI) with bacterial strain XeR6. All the 31 F1 plants and the paternal parent (CaFo) showed XeR6^S^ symptoms, including water-soaking, complete necrosis and abscission of the infected leaves, while the resistant maternal parent (Ca61R) displayed XeR6^R^ phenotype (Fig. 1). These results confirmed that: (i) susceptibility conditioned by the *Bs6*^*Fo*^ locus is dominant over the resistance conferred by *bs6*^*61*^; (ii) both *bs6*^*61*^ and *Bs6*^*Fo*^ loci were homozygous in the resistant and susceptible parents. The hybrid nature of F1 plants was further confirmed using polymorphic molecular markers. These results were consistent with the recessive nature of the XeR6^R^ trait of *bs6*^*60R*^ as demonstrated in the allelism test (see above) and reported previously (Vallejos et al. 2010).

Eleven F1 plants were subsequently self-pollinated over several generations yielding more than 4000 F2 seeds. Genetic mapping was initiated by determining the XeR6^R^/XeR6^S^ phenotypes and the genotypes for 30 molecular markers in 16 randomly selected XeR6^R^ and XeR6^S^ F2 plants (P1-P16). The molecular markers were distributed across all the 12 pepper linkage groups (LGs) (see Table S1 for marker and primer details). Genotyping revealed that markers Hpms1-5, CAMS-351 and EPMS-417 showed linkage to the XeR6^R^/XeR6^S^ phenotype with at least 1, 5 and 11 recombination events, respectively, among the 16 plants (Fig. S1). All three markers are located on LG6 of *C. annuum* cv. Perennial genome (https://www.ncbi.nlm.nih.gov/datasets/genome/; ASM2707371v1). Because all three markers (Hpms 1–5, CAMS-351 and EPMS-417) are located on chromosome 6 (Fig. S2), and the *bs6*^*61*^ locus showed genetic linkage to these markers, we concluded, that *bs6*^*61*^ locus is located on LG6 (chromosome 6) of *C. annuum*. This result is consistent with the mapping results previously reported by Sharma et al. (2023). Based on the genetic positions of the linked markers, we also concluded that the *bs6*^*61*^ locus was located on the opposite side of marker Hpms1-5 as compared to markers CAMS-351 and EPMS-417, that is on the proximal end of LG6, corresponding to a region of approximately up to 212 Mb on chromosome 6.

### Fine mapping of the ***bs6***^***61***^*** locus***

To identify the genetic position of the *bs6*^*61*^ locus on LG6 more precisely, fine mapping of the locus was carried out by generating seven polymorphic molecular markers in the interval from 205 to 212 Mb of LG6 in the CaPe genome sequence (Table S1). Genotyping of these markers together with phenotypic assessment of XeR6^R^/XeR6^S^ in 92 randomly selected F2 individuals (including the initial 16 plants, Fig. S1), enabled the *bs6*^*61*^ locus to be delimited between markers m1636 and m1652, spanning a region **<**1.4 Mb. The individuals which carried the informative recombination events were identified in plants P6 and P22 (Fig. S2).

**Fig. 2:**
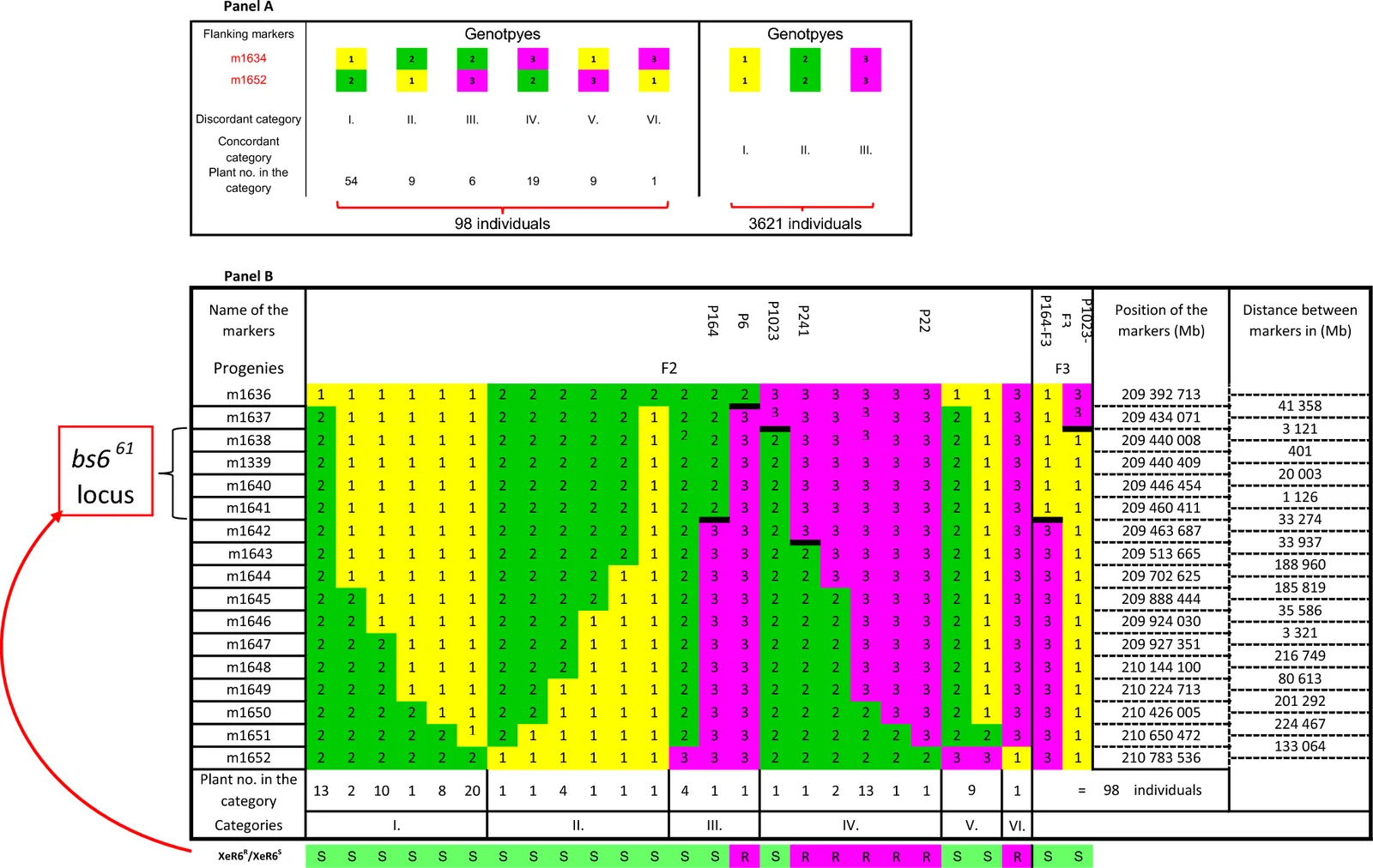
Genetic mapping of 98 F2 individuals with discordant genotype between markers m1636 and m1652. Susceptible and resistant phenotypes were determined as described in Materials and Methods. Polymorphic molecular genetic markers were collected from published sequences based on *C. annuum* 61R and *C. annuum* cv. ECW specific RNASeq reads: SRX30438917, SRX24240619-SRX24240625, SRX24240631, SRX24240642-SRX24240648. Polymorphism of the molecular markers (amplicon length) were converted to genotypes based on the parental pattern. In this figure, genotypes were represented by numbers (in colored background) as follows: 1 (yellow), paternal homozygotes; 2 (dark green), heterozygotes; 3 (purple). Genetic mapping was done by colormapping as described by Kiss et al. (1998). Bold horizontal bars in the colormap highlight the recombination sites that determined the location of the *bs6*^*61*^* locus*. Mb, megabase (pairs); CaPe, *C. annuum* var. Perennial; The positions of the markers were determined from the ASM2707371v1 genomic assembly of CaPe. The categories classified according to the genotypes of the end markers. Panel A. Number of individuals in the discordant categories I-VI. Panel B. Fine mapping of the *bs6*^*61*^* locus*. XeR6 phenotypes (XeR6^R^/XeR6^S^) are indicated as follows: plants labeled with number 5 in pale green background represent the susceptible phenotype; number 3 in purple background represent the resistant phenotype. Because no recombination events occurred between markers m1638 and m1641, the more precise localization of the *bs6*^*61*^* locus* could not been determined.

To further narrow down this interval, markers m1636 and m1652 were genotyped in more than 4000 F2 individuals to identify additional recombination events. As shown in Fig. 2A, 98 individuals had discordant genotypes, between these markers, and 3,621 showed concordant genotypes which were excluded from further analysis. The 98 recombinant individuals (Fig. 2A and B) were further analyzed with newly developed markers within the interval, and their XeR6^R^/XeR6^S^ phenotypes were determined. The genetic analysis of the genotypes and the phenotypes allowed us to further narrow down the position of the *bs6*^*61*^ locus. Plants P6 and P164 positioned the *bs6*^*61*^ resistance locus between markers m1636 and m1642, whereas plants P1023 and P22 positioned it between markers m1637 and m1643 (Fig. 2). Taken together, these recombination events localized *bs6*^*61*^ to the interval between markers m1637 and m1642, corresponding to an approximately 30 kilobase pairs (kb) region. This region was denoted CaFo-Bs6 DNA region or „Bs6^Fo^ region” for short for the susceptible plant CaFo, and Ca61R-bs6 region or „bs6^61^ region” for short, for the resistant plant Ca61R (the superscript characters were introduced to distinguish between plant lines). In recombinant plants P164 and P1023 (both were XeR6^S^), which carried flanking recombination breaks, the Bs6^Fo^ and bs6^61^ regions were present in heterozygous configurations (green color) on one side and in maternal homozygous configuration (purple color) on the other, but in reverse orientations (Fig. 2). These results indicate that the genetic determinants conditioning the XeR6^S^ and XeR6^R^ traits reside within the Bs6^Fo^ and bs6^61^ regions, respectively. To identify the causal genetic differences conditioning the susceptible and resistant phenotypes, the Bs6^Fo^ and the bs6^61^ regions were sequenced. These sequences were deposited into the GenBank under the accession numbers PZ343843 (Bs6^Fo^ region) and PZ343844 (bs6^61^ region), respectively.

### DNA sequence analysis of the Bs6^Fo^ and bs6^61^ regions of the parental plants

To characterize the fine-mapped Bs6^Fo^ and bs6^61^ regions and identify candidate functional sequences associated with the contrasting susceptible and resistant phenotypes, we compared their sequence organization, transcriptional activity, and conservation across available *C. annuum* genomes. The length of the nucleotide sequence of the Bs6^Fo^ and bs6^61^ regions were 29,743 bp and 29,991 bp in length, respectively. Both sequences can be artificially divided into two subregions, designated Bs6^Fo^A, Bs6^Fo^B and bs6^61^A, bs6^61^B regions, respectively.

(i) The Bs6^Fo^A and bs6^61^A regions (spanning from sequences 5’-TATTGGGTATTGAA to 5’-CACTTAGCCCAACCCAGTCT) were the only regions in which RNA transcripts and protein coding genes could be identified. The nucleotide sequences of the Bs6^Fo^A region of CaFo and the bs6^61^A region of Ca61R are shown in Figs. S3 and S4, respectively;

(ii) The Bs6^Fo^B and bs6^61^B regions (spanning from sequence 5’- AACCCACCTAAACC to 5’- CTAATTTATAAAACATATCTACACGTATACGTTAAA) comprised hypervariable regions with low complexity DNA sequences that were characterized by simple repetitive patterns. Importantly, these regions were transcriptionally inactive.

It is interesting to note that the DNA sequence of the Bs6^Fo^A region was identical in the following genomes of pepper cultivars/isolates: CaECW, CaFo, *C. annuum* ssp. F1-HDA149xHDA330 (GCA-033026605.1), *C. annuum* cv. PG1 (GCA-027035075.1), *C. annuum* cv. Maor (GCA-027073695.1) and *C. annuum* cv. LaMuyo-01. Similarly, the DNA sequence of the bs6^61^A region was identical to that of *C. annuum* cv. Perennial (GCA-027073715.1), Ca61R, *C. annuum* cv. CV3 (GCA-027073615.1) and *C. annuum* cv. ThaiHot (GCA-027073735.1).

### DNA sequence of the recombination sites in plants P164F3 and P1023F3

To resolve the informative recombination events and determine whether the *bs6*^*61*^ resistance determinant was located within the transcriptionally active portion of the fine-mapped interval, recombinant F3 lines were further analyzed. The informative recombination breakpoints flanking the *Bs6*^*Fo*^ / *bs6*^*61*^ loci occurred in plants P164 and P1023, respectively (Fig. 2). Because these regions were present in heterozygous configuration, and to facilitate phenotypic analysis and precise determination of the recombination sites, homozygous lines were generated by self-pollination. F3 progeny (individuals P164F3 and P1023F3) carrying the relevant recombination regions were selected for further analysis. In plant P164F3, the recombination event occurred between markers m1641 and m1642. To pinpoint the recombination site, the nucleotide sequence of the genomic region, amplified with primers pr1641-F and pr1642-R, was determined (Fig. S6). Since this genomic region corresponded to part of the Bs6^Fo^B and bs6^61^B regions of the parental genotypes and was transcriptionally inactive (see below)**,** it was excluded from further studies. Instead, we focused on the Bs6^Fo^A and bs6^61^A regions, assuming that the *bs6*^*61*^ genetic determinant is located within these transcriptionally active regions.

**Fig. 3:**
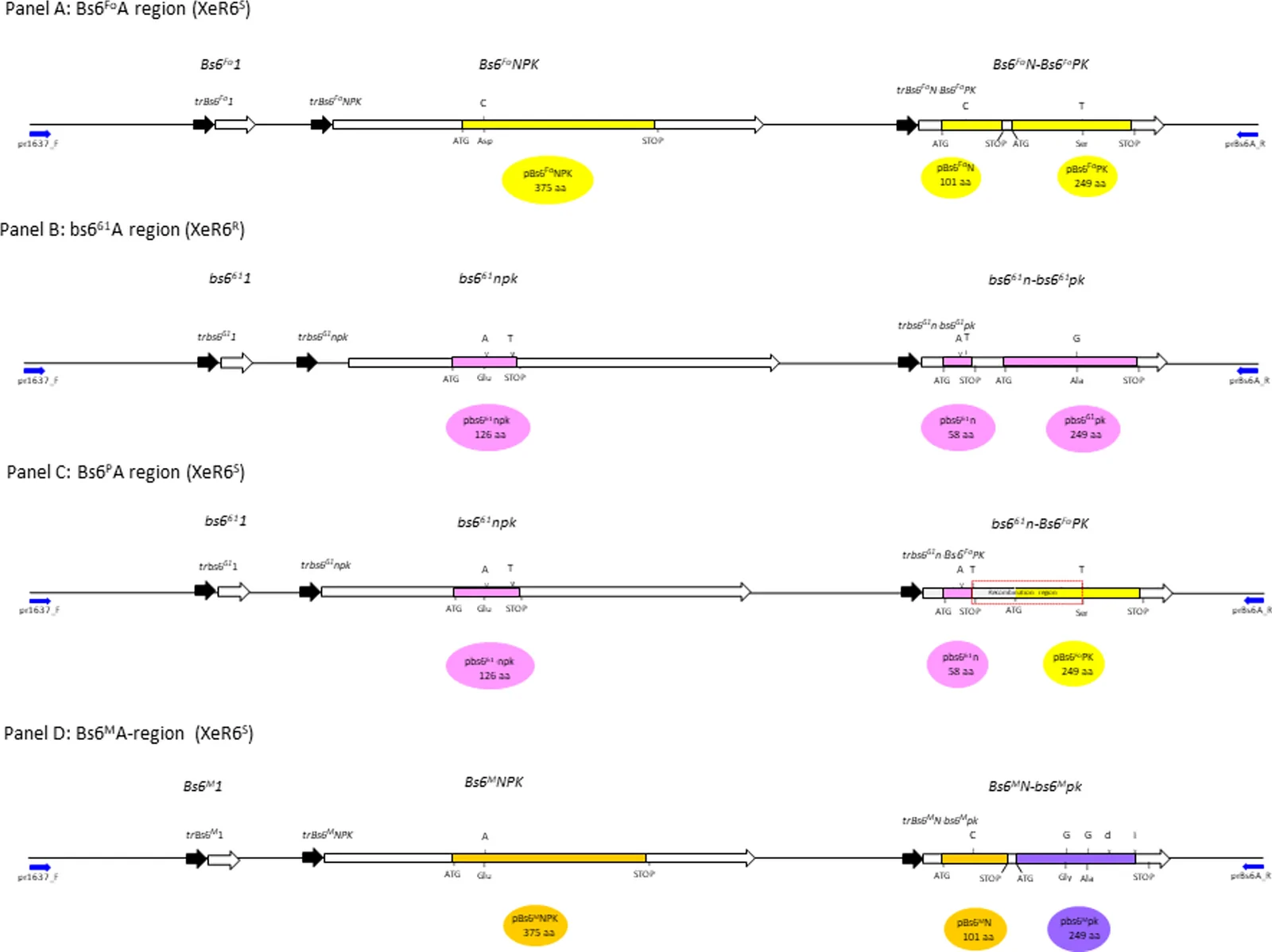
DNA, RNA and protein features of the Bs6^Fo^A, bs6^61^A, Bs6A^P^, and bs6A^M^ region of plants CaFo, Ca61R, P1023F3, CaMpR, respectively, between the flanking primers pr1637-F and prBs6A-R. Each panel (A, B, C, D) represents the schematic structure of the region including the name of the genes in italics (eg. *Bs6*^*Fo*^*NPK*, *Bs6*^*Fo*^*PK*, *bs6*^*61*^*N-bs6*^*61*^*PK*); the name of the transcripts with a mRNA prefix*;* the name of the proteins with a p prefix. Capital B in Bs6 and lower case b in bs6 terms refer to sensitive and resistant nature of the genes, mRNA and proteins, respectively. Open arrows, transcribed regions; yellow, purple and orange colors symbolize CaFo, Ca61R and CaMpR specific features, respectively. Other Abbreviations: XeR6^S^ and XeR6^R^ refer to susceptible and resistant nature of the plants; ATG, start codon; STOP, stop codon; nt, nucleotides; aa, amino acids; A, T, G, Ser and Ala abbreviations stand for adenine, thymine, guanine, serine and alanine, respectively; ORF starts with ATG and ends with STOP terms; black arrows: promoter regions; ellipsis represents proteins; v, insertion of a single nucleotide; vertical bar (l), indicates a position; blue arrows represent the position of the flanking primers. The length of arrows and lines are out of scale. For sequence informations see Figs. 4, S3, S4 and S6. The region where the recombination event occurred in P1023F3 is highlighted by a dotted red box and named Recombination region.

Similarly, the nucleotide sequence between recombination sites defined by markers m1637 and m1638 in plant P1023F3 was determined. The DNA sequence amplified with primers pr1637-F and prBs6A-R in plant P1023F3 was denoted P1023F3-Bs6A region or „Bs6^P^A region” for short. The nucleotide sequence of the Bs6^P^A region aligned to the corresponding sequence of their parental sequences is shown in Fig. S7. The section where the recombination event occurred (the “Recombination region”) is highlighted by red dotted box in Fig. 3, and by gray background in Fig. S7.

### RNA transcripts of the Bs6^Fo^A region

To identify transcriptionally active regions and candidate genes within the fine-mapped Bs6^Fo^ region, CaECW specific RNA-Seq reads showing 100% sequence identity were retrieved from the *Sequence Read Archives* (SRA) database of NCBI (https://www.ncbi.nlm.nih.gov/sra/?term=capsicum) and aligned to the DNA sequence template of the Bs6^Fo^ region **(**Camacho et al. 2009). The use of CaECW reads was appropriate as mentioned above, because the Bs6^Fo^ region of CaFo was identical to that of CaECW. This analysis revealed that the Bs6^Fo^B region was transcriptionally inactive, that is no RNA reads of 100% identity were found in the 15 SRX accessions of CaECW. In contrast, RNA reads were mapped into three transcriptionally active regions within the Bs6^Fo^A region. Similar results were obtained for the bs6^61^A region using Ca61R specific reads (SRX30438917, data not shown)**.** The three transcribed regions corresponded to three genes *Bs6*^*Fo*^*-1*, *Bs6*^*Fo*^*NPK*, *Bs6*^*Fo*^*N*-*Bs6*^*Fo*^*PK*, and *bs6*^*61*^*-1*, *bs6*^*61*^*npk*, *bs6*^*61*^*n-bs6*^*61*^*pk* in CaFo and Ca61R*,* respectively (Figs. 3, S3, S4 and S7).

### Proteins encoded in the Bs6^Fo^A regions

**Fig. 4:**
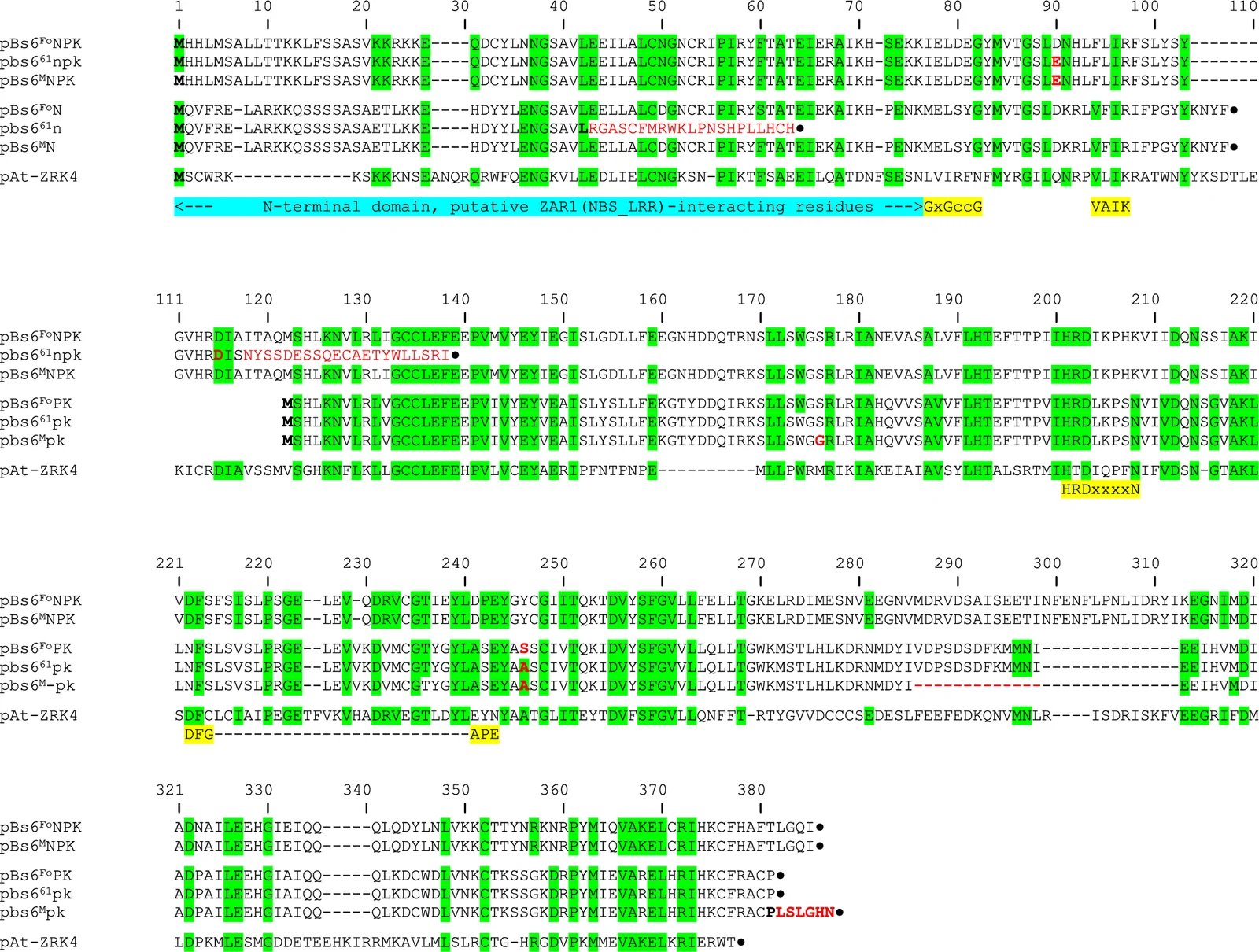
Multiple sequence alignment of the amino acid sequences deduced from coding regions of the Bs6^Fo^A, bs6^61^A, Bs6^P^A and Bs6^M^A regions, and ZRK4 protein of *A. thaliana* (as a representative of the pseudokinase family, protein ID NP-191333.1). NPK/npk domains contain both N-terminal and pseudokinase domains, while N/n and PK/pk domains contain only N-terminal and pseudokinase domains, respectively (see Fig. 3). N-terminal domain is highlighted under the sequences by light blue color. N-terminal domain contains amino acids of putative ZAR1-interacting residues (Seto et al. 2020). Glycine loop (GxGxxG), VAIK, HRDXXXXN, DFG—APE motifs are highlighted under the sequences by yellow color. Amino acid which are identical to that of ZRK4 are highlighted by green background color. ●, represents the position of stop codons, **M**, methionine start codons. -, indels. The amino acids after which the frameshift occurred are shown with **L** (position 42) and **D** (position 114) characters in pbs6^61^n and pbs6^61^npk, respectively, generating the truncated proteins pbs6^61^npk and pbs6^61^n, respectively.** S,** at position 246 in pbs6^61^pk, is serine residue, which changed to alanine in pbs6^61^pk

To characterize the candidate genes within the transcriptionally active Bs6^Fo^A regions and identify sequence differences potentially underlying the contrasting resistance and susceptibility phenotypes, we combined transcript analysis, sequence similarity searches, and comparative analysis of the predicted proteins in the susceptible CaFo, resistant Ca61R, and recombinant P1023F3 genotypes. Based on RNA transcript analysis combined with BLAST searches, three transcripts were identified in the Bs6^Fo^A region: *trBs6*^*Fo*^*-1*, *trBs6*^*Fo*^*NPK* and *trBs6*^*Fo*^*N-Bs6*^*Fo*^*PK*. Two of these transcripts, *trRNABs6*^*Fo*^*NPK* and *mRNABs6*^*Fo*^*N-Bs6*^*Fo*^*PK* encode three predicted proteins. The first transcript, *trRNABs6*^*Fo*^*-1* did not exhibit significant similarity to any sequences in the NCBI non-redundant protein sequence database. Furthermore, no functional known RNA sequences (tRNA, protein-coding mRNA, miRNA) was identified. As its biological function remains unknown, this transcript and this region was not investigated further in this study. The second transcript, *trBs6*^*Fo*^*NPK* encoded a predicted protein of 375 amino acids. Similarity searches (eg. UniProt BLAST search, https://www.uniprot.org/blast) revealed 35% sequence identity and 53% similarity to the *A. thaliana* ZRK4 pseudokinase (pAtZRK4), therefore we denoted this protein pBs6^Fo^NPK (NPK stands for **N**-terminal domain containing PseudoKinase domain). The third transcript (*trBs6*^*Fo*^*N-Bs6*^*Fo*^*PK*) encoded two ORFs, consequently two distinct proteins could be deduced. RNA-Seq analysis confirmed that both ORFs are transcribed as a single polycistronic transcript. Because these two proteins were similar to the N-terminal domain and the pseudokinase domain, of pBs6^Fo^NPK, they were denoted pBs6^Fo^N and pBs6^Fo^PK, respectively. The genomic organization of region, including the positions and coordinates of the transcripts, ORFs and the encoded proteins, is shown in Figs. 3, 4 and S3.

Similar to Bs6^Fo^A**,** the bs6^61^A region of Ca61R also encoded three proteins (Figs. 3 and S4). Gene *bs6*^*61*^*-1* resembled *Bs6*^*Fo*^*-1*, in the sense that no functional RNA could be identified, therefore this region was also excluded from further study. The predicted protein encoded by *bs6*^*61*^*npk* was a truncated counterpart of pBs6^Fo^NPK, therefore we denoted pbs6^61^npk. The shorter nature of pbs6^61^npk protein was the result of a single-nucleotide insertion causing frameshift and premature stop codon (Figs. S4 and S6). The third transcript, *trbs6*^*61*^*n-bs6*^*61*^*pk* also contained two ORFs similarly to the tr*Bs6*^*Fo*^*N*-*Bs6*^*Fo*^*PK* transcript in CaFo. The first predicted protein represented truncated version of pBs6^Fo^N due to a single-nucleotide insertion caused a frameshift and premature stop codon, therefore this protein was denoted pbs6^61^n. The second predicted protein corresponded to pBs6^Fo^PK except for a serine-to-alanine substitution at position 133. We denoted this protein pbs6^61^pk (Fig. S4).

The Bs6^P^A region of plant P1023F3 was a particularly informative recombinant DNA sequence because it carried a recombination breakpoint between genes *Bs6*^*Fo*^*N-Bs6*^*Fo*^*PK* and *bs6*^*61*^*n-bs6*^*61*^*pk*, respectively, highlighted by red dotted box and labelled „Recombination site” in Fig. 3C. The DNA sequences located to the left and right of the Recombination Site were identical to the corresponding sequences of the bs6^61^A and Bs6^Fo^A regions, respectively (Fig. S7). Two of the three encoded proteins (pbs6^61^npk and pbs6^61^n) were identical to the maternal proteins in Ca61R (compare Fig. 3B and C). Interestingly, the third protein, pBs6^Fo^PK was identical to the paternal protein pBs6^Fo^PK. This occurred because the recombination event took place within a region that was identical between the maternal and the paternal alleles. Consequently, the maternal specific protein pbs6^61^n and the paternal specific protein pBs6^Fo^PK became fused together generating a hybrid protein (Figs. 3C and S7).

### Predicted proteins encoded in the Bs6^Fo^A region belong to the pseudokinase family

To determine the predicted functional properties of the proteins in the Bs6^Fo^A region, we compared their sequences with homologous sequences in publicly available databases. As mentioned above, similarity search revealed that pBs6^Fo^NPK shares 35% identity and 53% similarity with the *A. thaliana* ZRK4 pseudokinase (pAtZRK4). Alignment of pBs6^Fo^NPK, pBs6^Fo^N and pBs6^Fo^PK with the ZRK4 sequence (Fig. 4), identified several conserved residues characteristic for the Receptor-Like Cytoplasmic Kinase (RLCK) XII-2 family of pseudokinases (Seto et al. 2020; Lewis et al. 2013; Wang et al. 2019a, b). Similar to other RLCK XII-2 family members, pBs6^Fo^NPK, pBs6^Fo^N and pBs6^Fo^PK proteins, showed alterations in the following motifs: the glycine loop (GxGxxG, binding and positioning ATP), the VAIK-lysine, the HRD-aspartate, the DFG-aspartate and the APE motif (located at the C-terminal end of the activation loop; Kwon et al. 2013; Roux et al. 2014). From these results, we concluded, that pBs6^Fo^NPK, pBs6^Fo^N and pBs6^Fo^PK of the Bs6^Fo^A region of CaFo belong to the RLCK XII-2 pseudokinase family and likely lack kinase activity. In Fig. 4, one can also see the alterations present in the protein sequences of pbs6^61^npk, pbs6^61^n, pbs6^61^pk, and pBs6^Fo^NPK, pBs6^Fo^N, pBs6^Fo^PK, respectively, resulting from mutations that occurred in the genes (*bs6*^*61*^*npk*, *bs6*^*61*^*n-bs6*^*61*^*pk*) of plant Ca61R (see also Fig. S4).

### Functional analysis of the genes in the Bs6^Fo^A region by VIGS

**Fig. 5:**
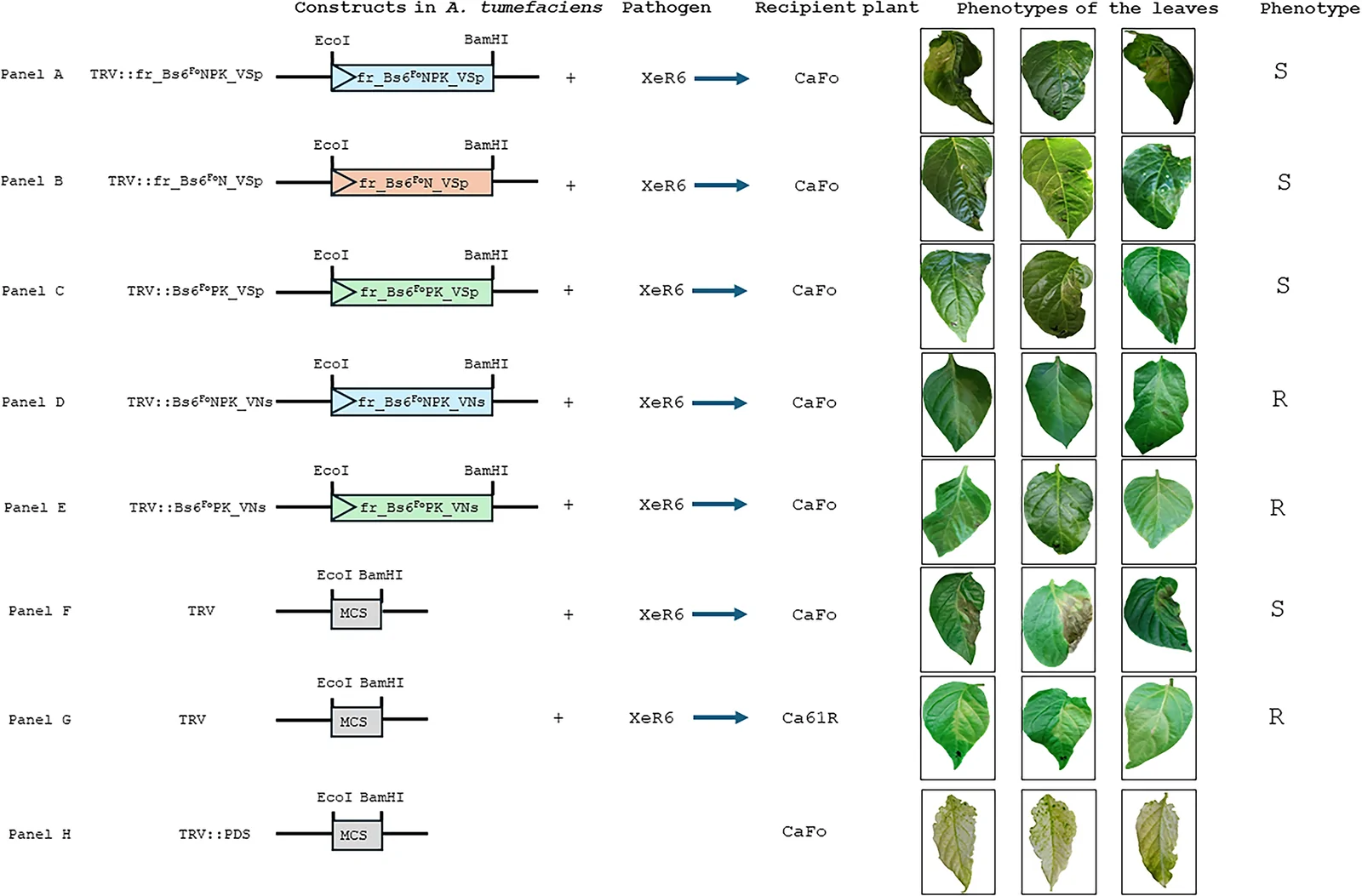
Virus Induced Gene Silencing of pseudogenes *Bs6*^*Fo*^*NPK*, *Bs6*^Fo^*N* and *Bs6*^Fo^*PK* reside in *C. annuum* cv. Feherozon. The specific (VSp) and non-specific (VNs) sequences were for gene *Bs6*^*Fo*^*NPK*: fragments fr_Bs6^Fo^NPK_VSp and fr_Bs6^Fo^NPK_VNs; for gene *Bs6*^*Fo*^*N-Bs6*^*Fo*^*PK*: fragments fr_Bs6^Fo^N_VSp, fr_ Bs6^Fo^PK_VSp and fr_Bs6^Fo^PK_VNs. The sequences of these fragments are listed in Table S2. The name, schematic representation of the constructs, the name of the pathogen, the name of recipient plant and the leaf phenotype after infection are shown in the picture. Symptoms were observed 6–10 days post-inoculation. More information can be found in the text and in the Materials and Methods.

To identify which of the candidate genes and predicted proteins within the fine-mapped *bs6*^*61*^ locus contribute to the resistant phenotype, we used virus-induced gene silencing (VIGS, Baulcombe 1999) to silence the corresponding genes selectively or simultaneously in the susceptible CaFo background. The map-based cloning of the *bs6*^*61*^ resistance determinant—as described above—delimited the bs6 resistance trait to two genes, *bs6*^*61*^*npk* and *bs6*^*61*^*n-bs6*^*61*^*pk*, encoding three predicted proteins, pbs6^61^npk, pbs6^61^n and pbs6^61^pk, respectively. Before investigating the function of these proteins, it was necessary to determine which of the two genes (or three proteins) conditions the resistant phenotype. We applied VIGS, a transient reverse genetics approach widely used to study gene function in a biological process by sequence-specific degradation of target mRNAs resulting in altered phenotypes. To perform VIGS experiment, gene-specific and gene non-specific DNA fragments of *Bs6*^*Fo*^*NPK* and *Bs6*^*Fo*^*N-Bs6*^*Fo*^*PK* were cloned into the TRV vector (Ratcliff et al. 2001) (Table S2). Systemic silencing efficiency was confirmed using a phytoene desaturase (*PDS*) gene construct as a positive control (Fig. 5H). The gene non-specific fragments were designed to generate siRNA populations with partial complementarity to both transcripts, thereby enabling simultaneous cross-silencing of the two genes. The constructs were then introduced into the susceptible CaFo plant by *A. tumefaciens*–mediated infiltration. As shown in Fig. 5A, B and C, silencing *Bs6*^*Fo*^*NPK* gene (with construct TRV::fr_Bs6^Fo^NPK_VSp), or *Bs6*^*Fo*^*N-Bs6*^*Fo*^*PK* gene (with either TRV::fr_Bs6^Fo^N_VSp, or TRV::Bs6^Fo^PK_VSp) construct individually did not alter the susceptible phenotype of CaFo plants, they remained sensitive (note that constructs TRV::fr_Bs6^Fo^N_VSp or TRV::Bs6^Fo^PKSp silence the same gene). In contrast, simultaneous silencing of both genes *Bs6*^*Fo*^*NPK* and *Bs6*^*Fo*^*N-Bs6*^*Fo*^*PK*, using non-specific fragments, which targeted both transcripts, rendered the plant resistant to XeR6 (Fig. 5D and E). High silencing efficiency was detected with *Bs6*^*Fo*^*NPK*, and *Bs6*^*Fo*^*N and Bs6*^*Fo*^*PK* gene specific primers as neither of the two transcripts could be detected on CaFo plants silenced with non-specific dual-silencing constructs (Fig. S5). On the other hand, infiltration with empty TRV vector did not affect the resistant/susceptible phenotype. From these results, we concluded that the presence of the mutant alleles of the three ORFs were required for conditioning bs6-mediated resistance phenotype (see Table 1 as summary).

**Table 1:**
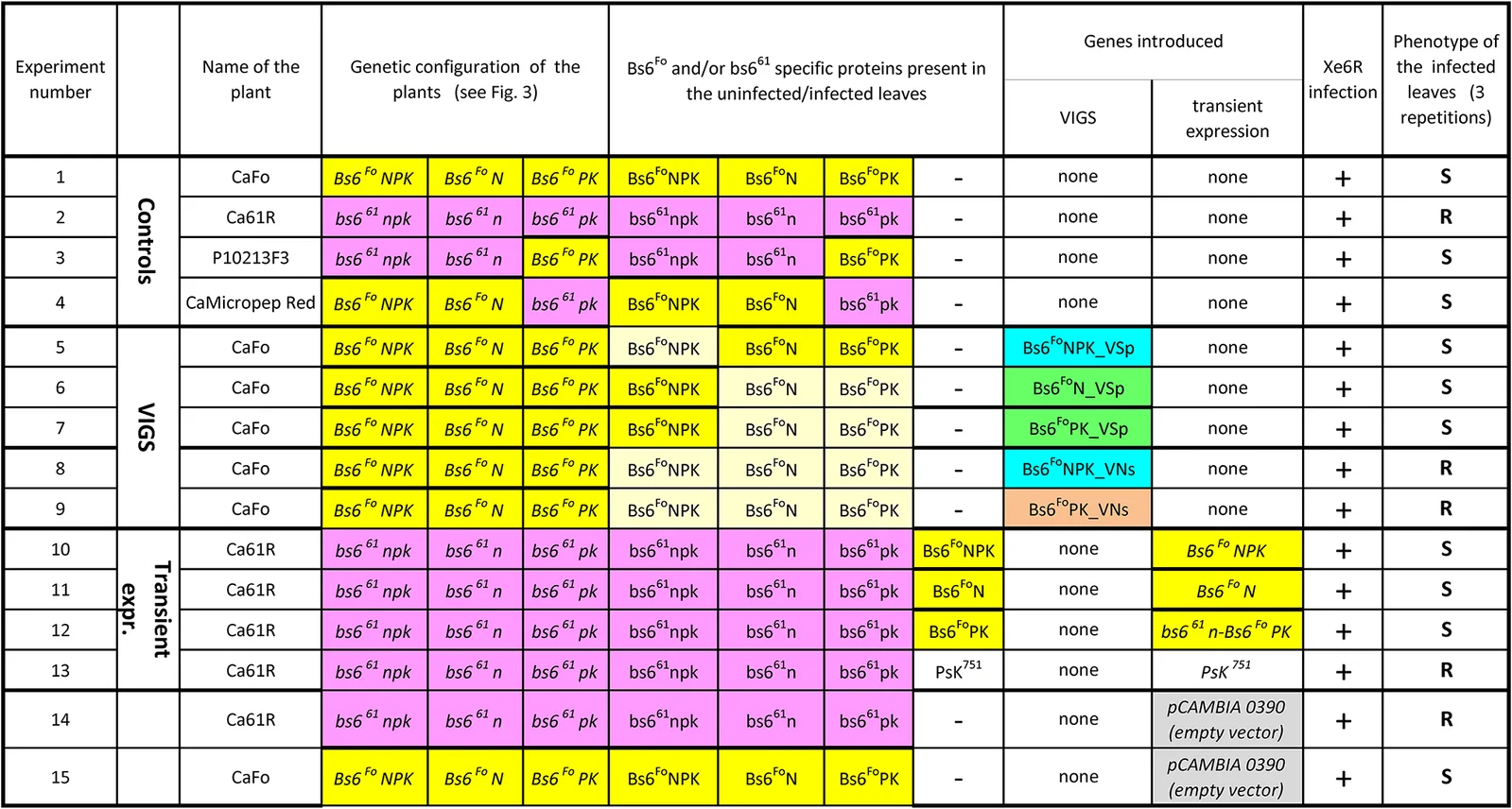
Summary of the genotypes and phenotypes of the Virus Induced Gene Silencing and transient expression experiments (S—indicates susceptible phenotype, R – indicates resistant phenotype)

Colors in the Table 1 are corresponding with the constructs introduced in the VIGS and transient experiment see also Fig. 5

### Functional analysis of the protein-coding sequences in the Bs6^Fo^A region

**Fig. 6:**
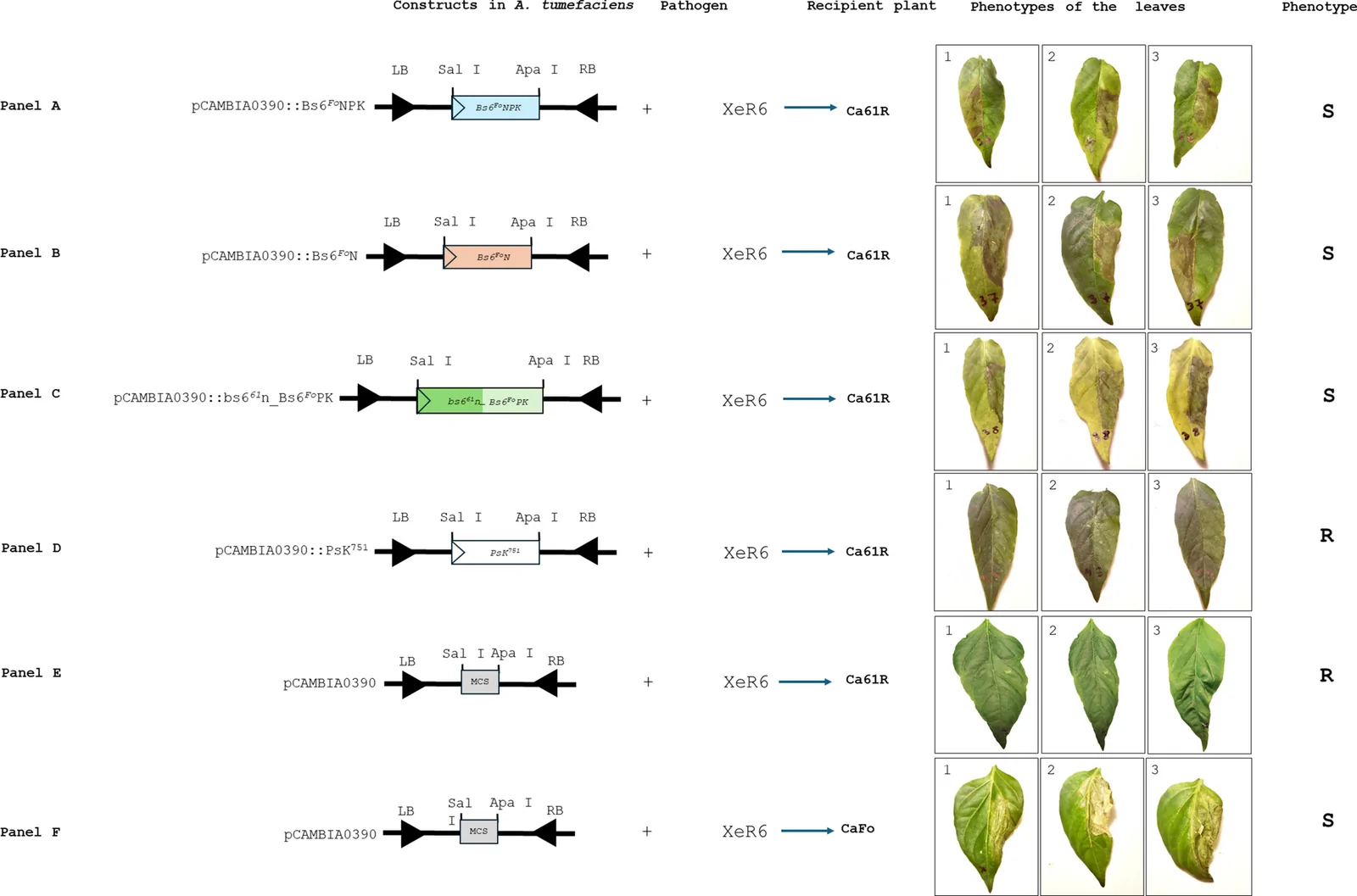
Transient gene expression in XeR6 resistant *C. annuum* var. 61R (Ca61R). The constructs used for transient gene expression studies were pCAMBIA0390-*Bs6*^*Fo*^*NPK*, pCAMBIA0390-*Bs6*^*Fo*^*N*, pCAMBIA0390-*bs6*^*61*^*n-Bs6*^*Fo*^*PK* and pCAMBIA0390-*Bs6*^*Fo*^*-PsK*^*751*^ (for constructs see Fig. 3). The promoter and coding sequence of these genes were cloned into plasmid pCAMBIA0390. Agrobacterium clones containing the above mentioned vectors were infiltrated into leaves of 4-week-old XeR6 resistant Ca61R plants. Bacterial inoculation was performed concurrently with infection, using 1–2 × 10^4^ XeR6 bacteria per leaf. After 7 days the disease symptoms were evaluated.

To determine the individual contributions of the three Bs6 protein-coding sequences to the susceptibility phenotype, we performed genetic complementation assays by expressing the corresponding susceptible alleles under their native promoters in the resistant Ca61R background. Based on the results obtained so far, the genes within the Bs6^Fo^A region appear to have redundant functions, since simultaneous silencing of both *Bs6*^*Fo*^*NPK* and *Bs6*^*Fo*^*N*-*Bs6*^*Fo*^*PK* genes were required to confer resistance in the susceptible CaFo background. To dissect the contribution of the individual protein-coding sequences to the susceptible/resistant phenotype, genetic complementation tests were performed, which can be considered as the reverse approach (as compared to VIGS) for functional analysis. Because the wild type *Bs6*^*Fo*^ alleles—conferring XeR6 susceptibility – behave as dominant genetic determinants, we hypothesized that expression of the corresponding susceptible alleles in the XeR6^R^ pepper plant Ca61R would restore susceptibility. Therefore the protein coding sequences of *Bs6*^*Fo*^*NPK*, *Bs6*^*Fo*^*N* and *Bs6*^*Fo*^*PK*, driven by their native promoters, were cloned into pCAMBIA 0390 vector (see constructs in Fig. 6) and transiently expressed in leaves of Ca61R plants by Agrobacterium-mediated infiltration. Clear susceptible symptoms (water-soaking, wet, discolored tissue and necrotic lesions)—similar to the infected sensitive plant CaFo—were observed after 10 days post-inoculation (DPI) on the leaves of Ca61R plants expressing the sensitive alleles of *Bs6*^*Fo*^*NPK*, *Bs6*^*Fo*^*N* and *bs6*^*61*^*n/Bs6*^*Fo*^*PK* (for constructs see Fig. 3 and leaf phenotype Fig. 6). In contrast, no susceptible symptoms were detected 10 dpi on leaves of Ca61R plant expressing another *Bs6*^*Fo*^ specific pseudokinase gene, PsK^751^ CaFo (VYZY01010751.1:168822–169922* C. annuum* cv ECW scaffold68364). Similarly, infiltration with the empty vector into either parental plants did not alter the resistant/susceptible phenotype (see for summary Table 1).

### Analysis of the recombinant plant P1023F3 and the *C. annuum* cv. MicropepRed line

To prove whether pBs6^Fo^PK alone is sufficient to confer susceptibility and to further assess the functional redundancy and contribution of the three Bs6 proteins to the XeR6 susceptibility phenotype, we analyzed the recombinant P1023F3 plant and the *C. annuum* cv. MicropepRed line. The analysis of P1023F3 plant suggested that the putative pBs6^Fo^PK protein may be responsible for susceptibility, because the other two proteins, pbs6^61^npk and pbs6^61^n, are truncated and non-functional. (note that considering the sensistive P1023F3 and the resistant Ca61R plants there is only one amino acid difference between pbs6^61^npk, pbs6^61^n-Bs6^Fo^PK and pbs6^61^npk, pbs6^61^n-bs6^61^pk, respectively, taking into account the two proteins in question). However, the predicted pBs6^Fo^PK protein was encoded by the second ORF of the single transcript *trRNABs6*^*61*^*N-Bs6*^*Fo*^*PK* derived from the *Bs6*^*61*^*N*-*Bs6*^*Fo*^*PK* gene (see Fig. 3), raising the question whether this ORF is translated in vivo. To answer this question, DsRed (Discosoma Red) fluorescent protein reporter constructs (pBs6^Fo^N^+^-DsRed and p35S::dsRed, Fig. S8) were transiently expressed in *N. benthamiana* leaves. Transient expression analysis revealed that both constructs, including the p35S::DsRed positive control, were expressed in *N. benthamiana* leaf cells (Fig. S8). These results provide evidence that protein pBs6^Fo^PK in fact can be translated as the second ORF of the *trRNAbs6*^*61*^*n-Bs6*^*Fo*^*PK* transcript and therefore, it alone could account for the susceptible phenotype of plant P1023F3.

However, the possibility that the pBs6^Fo^PK protein, which has a serine residue as compared to an alanine in pbs6^61^pk protein, (see Fig. 3) might be the only gene in the Bs6A region responsible for the susceptibility phenotype was considered unlikely because of the results of the VIGS and complementation experiments, as well as the XeR6^S^ phenotype of plant CaMpR. Plant CaMpR was sensitive to XeR6 race, since the pbs6^M^PK protein is predicted to be non-functional having two amino acid substitutions and two deletions, as compared to pBs6^Fo^PK (Figs. 3 and 4). In contrast, the two other proteins, pBs6^M^NPK and pBs6^M^N, are predicted to remain functional.

Taking together, the results of the VIGS and genetic complementation experiments, as well as the phenotype of plants P1023F3 and CaMpR, strongly suggest that the three proteins have functionally redundant and mutually substitutable roles, such that any one of them is sufficient to confer susceptibility. Conversely, resistance requires that all three proteins be non-functional, as observed in the resistant Ca61R line.

## Discussion

In this study, we applied a map-based cloning approach to clone the *bs6*^*61*^ locus which confers a high level of recessive resistance against all races of *X. euvesicatoria* in pepper (*C. annuum* var. 61R, in short Ca61R). The XeR6^R^ resistant plant Ca61R showed no hypersensitive reaction (HR, Klement et al. 1964; Balint-Kurti 2019) on their leaves after XeR6 infection – similarly to the previously described bs5 resistance (Vallejos et al. 2010; Szabó et al. 2023). In contrast to the rapid and localized cell death characteristic of the hypersensitive response, the inoculated leaves of Ca61R remained viable with only a slight pale green discoloration visible at the inoculation site. This non-hypersensitive resistance phenotype may represent an important agronomic advantage of recessive resistance alleles. In particular, recessive resistance genes such as *bs5* and *bs6*, which can be potentially exploited by targeted gene editing, provide valuable resources for breeding durable disease-resistant cultivars. To date, no breakdown of *bs5*- or *bs6*-mediated resistance by *X. euvesicatoria* has been reported, and the absence of extensive localized cell death preserves viable leaf tissue following infection. These characteristics highlight the potential value of recessive alleles of host susceptibility genes that confer resistance in pepper (Jones et al. 2002).

Map based cloning was conducted by crossing the bs6 resistant line Ca61R carrying *bs6*^*61*^ locus with the susceptible parent *Capsicum annuum* var. Feherozon (CaFo). F1 plants were self-pollinated and F2 seeds were collected. More than 4000 individual F2 plants were genotyped by polymorphic molecular PCR markers and tested for XeR6 sensitivity/resistance. As a result, the *bs6*^*61*^ genetic determinant was localized within a ~28 kb DNA region, the bs6^61^ region, on chromosome 6 of pepper (Fig. 2). Gene expression studies by aligning RNASeq reads, revealed that RNA molecules were transcribed only from the Bs6^Fo^A and bs6^61^A regions, respectively, therefore we supposed, that the *bs6* resistance trait was conditioned in this transcriptionally active region. The analysis of the DNA/RNA sequences of the bs6^61^A/Bs6^Fo^A regions elicited that out of the three transcripts (*trBs6*^*Fo*^*1*, *trBs6*^*Fo*^*NPK* and *trBs6*^*Fo*^*N-Bs6*^*Fo*^*PK*) only two (*trBs6*^*Fo*^*NPK* and *trBs6*^*Fo*^*N-Bs6*^*Fo*^*PK*) encoded ORFs (Open Reading Frames). As seen in Fig. 3, the two transcripts (*Bs6*^*Fo*^*NPK* and *Bs6*^*Fo*^*N-Bs6*^*Fo*^*PK*) of the Bs6^Fo^A region codes for three predicted proteins (pBs6^Fo^NPK, pBs6^Fo^N and pBs6^Fo^PK) and these deduced proteins should be considered as functional, wild type proteins conditioning susceptible phenotype in CaFo. On the other hand, the three ORFs (pbs6^61^npk, pbs6^61^n and pbs6^61^pk) in the bs6^61^A region of Ca61R were considered as protein products of the mutated alleles (Fig. 4) of the two genes (*bs6*^*61*^*npk and bs6*^*61*^*N-bs6*^*61*^*PK*). Protein sequence analysis revealed, that the wild type and the mutant proteins, respectively, are homologous to the Receptor-Like Cytoplasmic Kinase (RLCK) XII-2 family of pseudokinases (Fig. 4; Kwon et al. 2019; Zulawski et al. 2014). The three predicted proteins (pBs6^Fo^NPK, pBs6^Fo^N and pBs6^Fo^PK)—considered wild type ones—were similar to each other in such a way that the N-terminal domain of pBs6^Fo^NPK was homologous to pBs6^Fo^N (60% identity), while the C-terminal domain was homologous to pBs6^Fo^PK (61% identity, see alignment in Fig. 4). Another interesting feature of proteins pBs6^Fo^N and pBs6^Fo^PK was that they were encoded by one mRNA molecule, therefore this transcript must be considered polycistronic—strictly speaking dicistronic—mRNA. One should ask the question whether or not the second ORF (encoding pBs6^Fo^PK) of *trBs6*^*Fo*^*N-Bs6*^*Fo*^*PK* is in fact translated. Using DsRed fusion construct, it was demonstrated that the DsRed protein in fact was translated from a gene fusion *Bs6*^*Fo*^*N-DsRed* (Fig. S8). This translation requires an internal ribosomal entry site (IRES) sequence (Hellen and Sarnow 2001; Zhao et al. 2020), which will be investigated in the future.

In the susceptible plant CaFo, three proteins (pBs6^Fo^NPK, pBs6^Fo^N and pBs6^Fo^PK) were functional in the Bs6^Fo^A region, while in contrast in the resistant plant Ca61R all three orthologue proteins (pbs6^61^npk, pbs6^61^n and pbs6^61^pk) were mutated, consequently it was not evident which gene conditioned the XeR6 susceptible/resistant phenotype. To answer this question, the two transcripts coding for the three proteins were independently silenced in VIGS experiments, which showed that none of these two silenced mRNAs alone resulted in the resistant phenotype. On the other hand, when both mRNAs were silenced simultaneously resistant phenotype appeared. In a reverse situation, when one of the two wild type transcripts (*trBs6*^*Fo*^*NPK* or *trBs6*^*Fo*^*N-Bs6*^*Fo*^*PK*) was transiently expressed in the leaves of the resistant plant Ca61R, the resistant trait became sensitive. These results clearly indicated that the function of the three proteins (pBs6^Fo^NPK, pBs6^Fo^N and pBs6^Fo^PK) were redundant, and that the non-functional alleles of all three proteins (pbs6^61^npk, pbs6^61^n and pbs6^61^pk) were required for the formation of *bs6* resistance. The sensitive nature of the recombinant plant P1023F3 and CaMpR fits perfectly into this picture (see Table 1 for summary).

Similarity search of the three wild type proteins (pBs6^Fo^NPK, pBs6^Fo^N and pBs6^Fo^PK) in the Bs6^Fo^A region revealed that they were homologous to the Receptor-Like Cytoplasmic Kinase (RLCK) XII-2 family of pseudokinases (Zeqiraj and van Aalten 2010; Kwon et al. 2019). Pseudokinases have lost phosphotransferase activity but nonetheless perform critical non-enzymatic roles (Boudeau et al. 2006; Lewis et al. 2013; Blaum et al. 2014; Roux et al. 2014; Wang et al. 2015; Murphy et al. 2017; Bastedo et al. 2019; Seto et al. 2020; Brauer et al. 2022; Diplock et al. 2024). Pseudokinases lack at least one key amino acid normally required for metal and adenosine triphosphate (ATP) binding and for catalysis (Hanks and Hunter 1995; Boudeau et al. 2006; Eyers and Murphy 2013). Prominent catalytic motifs include the “catalytic triad” residues, composed of the ATP-binding beta3 lysine, the catalytic aspartate within the catalytic loop His-Arg-Asp-X-X-X-X-Asn (HRDXXXXN) motif, and the metal-binding aspartate of the activation loop Asp-Phe-Gly (DFG) and APE motif (Roux et al. 2014; Kwon et al. 2019; Seto et al. 2020). As shown in Fig. 4, none of the proteins (pBs6^Fo^NPK, pBs6^Fo^N and pBs6^Fo^PK) conditioning susceptible trait contains consensus catalytic motifs, therefore they were considered members of genuine pseudokinases, although catalytic kinase activity had not been tested in this study.

The fundamental role of pseudokinases in plant-pathogen interactions has been studied in most detail in *Arabidopsis* systems. Pseudokinases, such as ZED1 and some ZRKs, form protein–protein complexes with ZAR1-type proteins which are NBS-LRR type R-proteins. These complexes are involved in ETI (Effector-Triggered Immunity) and defend (in many but not in all cases) the plants against pathogenic bacterial attack (Lewis et al. 2013; Blaum et al. 2014; Wang et al. 2017; Bastedo et al. 2019; Diplock et al. 2024; Roux et al. 2014). In general, pseudokinases – which can be called allosteric activators—are molecules that bind to enzymes or receptors at sites distinct from the active (orthosteric) site, inducing a conformational change that increases (or abolishes) the protein’s activity or affinity for its substrate. They enhance/prevent enzymatic functions and are essential for regulating metabolic pathways.

It is currently unknown how the pBs6^Fo^NPK, pBs6^Fo^N, and pBs6^Fo^PK proteins perform their redundant functions on their own, leading to sensitivity. It can be assumed, that when these three genes—alone or in any combination—are in a functional state, the ETS (Effector-Triggered Susceptibility) program prevails and is implemented although several of the resistant genes under the control of ETI (Effector-Triggered Immunity) or PTI (Pattern-Triggered Immunity) are also expressed (Subedi et al. 2024). That is, in simpler terms, the effect of the genes responsible for susceptibility manifests and efficiently functions, irrespective of those resistant genes that are expressed following XeR6 infection in the susceptible CaFo plant. It might well be, that in the presence of functional pBs6^Fo^NPK, pBs6^Fo^N, and pBs6^Fo^PK proteins, one or more of the resistance genes were not expressed which are crucial for establishing resistance. However, when *Bs6*^*Fo*^*NPK*, *Bs6*^*Fo*^*N*, and *Bs6*^*Fo*^*PK* genes are simultaneously mutated resulting in functionless protein products (pbs6^61^npk, pbs6^61^n—which are truncated proteins—and pbs6^61^pk, which carries a serine to alanine change), then the whole scenario changes by allowing expression of essential resistant genes and ETI manifests by suppressing/overcoming ETS and providing a high level of resistance against *X. euvesicatoria* in pepper. Moreover, they not only provide resistance, but unlike HR-type resistance where rapid cell death occurs to prevent the spread of pathogens—the leaf tissue remains alive and active in such a way that the plant is healthy, exhibiting normal growth and development and most importantly they are successfully and profitably being used in agriculture for pepper cultivation.

In conclusion, proteins pBs6^Fo^NPK, pBs6^Fo^N, and pBs6^Fo^PK conditioning Bs6 susceptibility are pseudokinases which may be allosteric inhibitor molecules (Zeqiraj and van Aalten 2010) that bind to enzymes e.g., NBS-LRR receptors (kinases) at the active or other sites of the enzyme and prevent enzymatic functions leading to signal transduction that are essential for triggering ETI. If they are mutated, the putative NBS-LRR receptors (kinases) may be free to interact with *X. euvesicatoria* effectors to initiate the expression of resistant genes. Exploring this pathway at the molecular level—using yeast two-hybrid systems or other means – can reveal the detailed mechanism of this pathway, which is necessary for understanding this new mechanism of effector-triggered resistance at the molecular level.

## Supplementary Information

Below is the link to the electronic supplementary material.

Supplementary file1 (DOCX 6559 KB)

Supplementary file2 (PDF 519 KB)

Supplementary file3 (PDF 585 KB)

Supplementary file4 (PDF 151 KB)

Supplementary file5 (PDF 319 KB)

Supplementary file6 (TIF 223 KB)

Supplementary file7 (PDF 56 KB)

Supplementary file8 (PDF 177 KB)

Supplementary file9 (TIF 348 KB)

Supplementary file10 (PDF 135 KB)

Supplementary file11 (PDF 67 KB)

## Data Availability

All data supporting the findings of this study are available within the paper and its Supplementary Information. NGS data is available on NCBI SRA database under SRX30438917.
