# Supplementary material for: Map-based cloning of the susceptibility locus conditioning bs6 resistance in pepper (Capsicum annuum L.) encoding mutant alleles of pseudokinase proteins
Source: Theor Appl Genet. 2026 Oct 20;139(11):308. doi: 10.1007/s00122-026-05407-2 (PMC13683501; doi:10.1007/s00122-026-05407-2)
Supplement: Supplementary file 1 [file 122_2026_5407_MOESM10_ESM.pdf]

Table S1

	Name of the primers	Sequence of the primers	Length of the primers (nt)	Length of the amplicons (nt)	Type of polymorphism	References
**Primers for genetic mapping**	prBs6A_R	AGACTGGGTTGGGCTAAGTG	20	Not relevant		**This study**
	pr1634_F	TCCATTTTCTGTACGGATTAGCT	23	291	SSCP, PAGE	
	pr1634_R	AAAACTCAGACTATGGCAGACT	22			
	pr1635_F	CTCACTCAACTGCAAACTACTC	22	300	SSCP, PAGE	
	pr1635_R	ATGGGGTAGAAATCAGGCTCAA	22			
	pr1636_F	AAAATCATGACGAACTCAAA	20	382	SSCP, PAGE	
	pr1636_R	TGATTTATAGTCACGCAAATAGGG	24			
	pr1637_F	TATTGGGTATTGAAGTTTGTGCTG	24	1007	InDel, Agarose	
	pr1637_R	TGCTGCTATGAAGAGTCCAAGTAG	24			
	pr1638_F	GAGACTTGTCGGTTGCTGTC	20	423	SSCP, PAGE	
	pr1638_R	ACAACTCCGAAGCTATAGACGT	22			
	pr1639_F	ACGTCTATAGCTTCGGAGTTGT	22	1005	InDel, Agarose	
	pr1639_R	TTGTTGAGTTATTAGAGGCAATGA	24			
	pr1640_F	ACACCTCTCTAGCAGTCTCCCTAA	24	198	SSCP, PAGE	
	pr1640_R	TCAGCATAGGATGTTAGTTCTGGA	24			
	pr1641_F	TAAAATTGGTAGTGCAACACATGC	24	161	SSCP, PAGE	
	pr1641_R	CATTGAAGAGCCTGTAAAAGACAA	24			
	pr1642_F	CGATTTAGATTTTGAGTAAAGGTTCA	26	375	SSCP, PAGE	
	pr1642_R	TTTATTTTTCATCCCCGTAATTTG	24			
	pr1643_F	CCGAATTCCCATCCGTTACTTC	22	242	SSCP, PAGE	
	pr1643_R	ATTCGATACAGCAACCGACAAG	22			
	pr1644_F	CACCCAAATATCATTGCTTGAA	22	195	SSCP, PAGE	
	pr1644_R	ATAACAAACTTCATGCTCAACATT	24			
	pr1645_F	GTGTGGGACCTATATTTTGTCGA	23	245	SSCP, PAGE	
	pr1645_R	CCCAAGGAGTCATGTTTGCA	20			
	pr1646_F	AAGGGGCATGTCGGTTTGAT	20	314	SSCP, PAGE	
	pr1646_R	TGCATGAACAATACTGACTGAATGA	25			
	pr1647_F	AGGGGTAGAACATCTAGGCCA	21	306	SSCP, PAGE	
	pr1647_R	ACGAGTGACCACATCAGGAG	20			
	pr1648_F	GCTTCATTGACCCGGTGTTT	20	307	SSCP, PAGE	
	pr1648_R	TTATGTCCTCCTCGGCTTCC	20			
	pr1649_F	TGACTTTAATCCTATGTATGTGAAGTC	27	306	SSCP, PAGE	
	pr1649_R	AACAAAATGCGCTACCATCG	20			
	pr1650_F	GATCCATCTCATCTTGAAGCCT	22	240	SSCP, PAGE	
	pr1650_R	TCCTCATTCCGAATCCCCTT	20			
	pr1651_F	GTTTTACTATTCTAGACCAACTCC	24	140	SSCP, PAGE	
	pr1651_R	TTCGTAACATATGCACACTCACA	23			
	pr1652_F	AAGCGCTCTCTATATTGGCTACA	23	350	SSCP, PAGE	
	pr1652_R	TGCATTTGTTCTATTGTGGAAT	22			
	pr1653_F	GTTGGATTTTGACACACAAATTAAG	25	290	SSCP, PAGE	
	pr1653_R	CGGGTGTAATTACTGAAATGGACT	24			
	pr1654_F	GCTCATTTAATTGCAACCTTATCT	24	330	SSCP, PAGE	
	pr1654_R	ACACACATCAATTTCACAGGAATC	24			
	pr1655_F	CATTTGTATCGGTTCACATTACG	23	467	SSCP, PAGE	
	pr1655_R	GCTTTCTGATGTGCGGTATG	20			
	Hpms 1-5 F	CCAAACGAACCGATGAACACTC	22	313	SSR, NuSieve agarose	Lee et al., 2004
	Hpms 1-5 R	GACAATGTTGAAAAAGGTGGAAGAC	25			
	CAMS-351 F	CGCATGAAGCAAATGTACCA	20	214	SSR, NuSieve agarose	Kwon et al., 2013
	CAMS-351 R	ACCTGCAGTTTGTTGTTGGA	20			
	EPMS-417 F	CGCATATACATACATAAATTCTTTC	25	113	SSR, NuSieve agarose	Nagy et al., 2007
	EPMS-417 R	TCAACATCTCACCGAAGCTG	20			
**Primers for contructs**	prBs6^{Fo}-NPK_VSpE	ctagaattcGTGCGGAACAATCGAGTATTTAGA	**33**	**255**	**Not relevant**	**This study**
	prBs6^{Fo}-NPK_VSpB	tcaggatccATATAGCGATCGATGAGGTTAGGT	**33**			
	prBs6^{Fo}-NPK_VNsE	ctagaattcGATTACGAATTGCCAATGAGGT	**31**	**441**		
	prBs6^{Fo}-NPK_VNsB	tcaggatccAGCGATCGATGAGGTTAGGTAG	**31**			
	prBs6^{Fo}-N_VSpE	ctagaattcACTGCCACTGAGATAGAAAAAGCA	**33**	**158**		
	prBs6^{Fo}-N_VSpB	tcaggatccTAAAAGTAATTTTTATAATATCCG	**33**			
	prBs6^{Fo}-PK_VSpE	ctagaattcGTCGGTTGCTGTCTAGAATTCG	**31**	**194**		
	prBs6^{Fo}-PK_VSpB	tcaggatccAAAACGACTGCAGAAACGACCT	**31**			
	prBs6^{Fo}-PK_VNsE	ctagaattcTCGAAGAACCAGTTATAGTGTATGA	**34**	**322**		
	prBs6^{Fo}-PK_VNsB	tcaggatccTTTTACTACTTCCAATTCTCCCCG	**33**			
	prBs6^{Fo}-NPK-SalI	tcagtcgacATACCTCGCTGTCAAC	**25**	**3164**		
	prBs6^{Fo}-NPK-ApaI	ctagggcccCTAAATCTGGCCAAGAGTGA	**29**			
	prBs6^{Fo}-N-SalI	tcagtcgacCTATATAAGATGGGGAAAAAAATTAAACA	**38**	**1771**		
	prBs6^{Fo}-N-ApaI	ctagggcccTTAAAAGTAATTTTTATAATATCCGGGG	**37**			
	prbs6^{61}-n-SalRI	tcagtcgacCTATATAAGATGGGGAAAAAAATTAAACA	**38**	**2142**		
	prbs6^{61}-N-SalI-R	TTGCTACATCTCGAAAAATATTAAAAGTAATTTTTATAATATCC	**44**			
	prBs6^{Fo}-PK-F-ApaI	ATATTATAAAAATTACTTTTAATATTTTTCGAGATGTAGCAAT	**43**	**812**		
	prBs6^{Fo}-PK-ApaI	ctagggcccCTAAGGGCATGCACG	**24**			
	prPsK^{751}-SalI F	tcagtcgacCAGGAGAGCTAGGAGTTGGAAA	**38**	**2199**		
	prPsK^{751}-ApaI R	ctagggcccCTAATTCCGGCAAAGAGTGAGG	**45**			
	prbs6^{61}-N-DsRed_SalI	tcagtcgacCTATATAAGATGGGGAAAAAAATTAAACA	**61**	**699**		
	prbs6^{61}-N_prBs6^{S}-PK	AAGCGCATGAACTCCTTGATTACATTTTTGAGATGGCTCATCTGA	**28**			
	prBs6^{Fo}-PK-DsRed F	ATTACTGCTCAGATGAGCCATCTCAAAAATGTAATCAAGGAGTTCATG CGCTTCAAGGTGC	**31**	**2904**		
	prDsRed-SalIR	tcagtcgacCTACAGGAACAGGTGGTGG	**31**			
**Primers for qPCR**	prBs6Fo-NPK_qPCR F	CACTCTGAAAAGAAAATAGAAC	22	**143**	**Not relevant**	**This study**
	prBs6Fo-NPK_qPCR R	TTGAGATGACTCATCTGAGCTG	22			
	prBs6^{Fo}-N_qPCR F	ATGCAGGTCTTTAGGGAACTCG	22	**153**		
	prBs6^{Fo}-N_qPCR R	GGGAATTCGGCAATTTCCATCG	22			
	prBs6^{Fo}-PK_qPCR F	CTTCGGAGTTGTTCTCCTTCAG	22	**133**		
	prBs6^{Fo}-PK_qPCR R	ATGAATCTCTTCAATGTTCATC	22			
	prGADPH_qPCR F	CTGCTGCCCACTTGAAGGGT	20	**161**		Zhan et al., 2025
	prGADPH_qPCR R	GCCAGAGGAGCAAGGCAGT	19			

I
