# Supplementary material for: Map-based cloning of the susceptibility locus conditioning bs6 resistance in pepper (Capsicum annuum L.) encoding mutant alleles of pseudokinase proteins
Source: Theor Appl Genet. 2026 Oct 20;139(11):308. doi: 10.1007/s00122-026-05407-2 (PMC13683501; doi:10.1007/s00122-026-05407-2)
Supplement: Supplementary file 2 [file 122_2026_5407_MOESM11_ESM.pdf]

fr_Bs6^{Fo}NPK_VSp

ctagaattcGTGCGGAACAATCGAGTATTTAGATCCAGAATATGGCTACTGTGGTATTATTACTCAAAAAACTGACGTGTACAGCTTTGGAGTTCTTCTCTTTGAGCTATTAACT
GGAAAGGAATTGCGTGATATTATGGAATCCAACGTTGAAGAGGGTAATGTTATGGATAGAGTGGATTCTGCAATTTCAGAAGAGACAATAAATTTTGAAAATTTTCTACCTAACC
TCATCGATCGCTATATggatcctga

fr_Bs6^{Fo}N_VSp

ctagaattcACTGCCACTGAGATAGAAAAAGCAATAAAGCACCCTGAAAACAAAATGGAGCTTTCCTATGGGTATATGGTTACAGGATCACTGGACAAACGCCTCGTTTTCATTA
GGATTTTCCCCGGATATTATAAAAATTACTTTTAggatcctga

fr_Bs6^{Fo}PK_VSp

ctagaattcGTCGGTTGCTGTCTAGAATTCGAAGAACCAGTTATAGTGTATGAATATGTTGAGGCTATAAGTCTTTATTCTTTACTTTTTGAAAAGGGGACATATGATGATCAAA
TTAGAAAATCATTACTATCTTGGGGAAGTAGACTACGTATAGCCCACCAGGTCGTTTCTGCAGTCGTTTTggatcctga

fr_Bs6^{Fo}NPK_VNs

ctagaattcGATTACGAATTGCCAATGAGGTTGCTTCTGCACTCGTCTTTCTCCATACCGAATTTACTACACCTATCATCCACAGAGATATAAAGCCTCACAAAGTGATAATAGA
TCAGAATAGCAGCATTGCCAAAATAGTGGACTTCTCATTTTCCATATCATTGCCTTCAGGGGAATTGGAGGTACAAGATCGTGTGTGCGGAACAATCGAGTATTTAGATCCAGAA
TATGGCTACTGTGGTATTATTACTCAAAAAACTGACGTGTACAGCTTTGGAGTTCTTCTCTTTGAGCTATTAACTGGAAAGGAATTGCGTGATATTATGGAATCCAACGTTGAAG
AGGGTAATGTTATGGATAGAGTGGATTCTGCAATTTCAGAAGAGACAATAAATTTTGAAAATTTTCTACCTAACCTCATCGATCGCTggatcctga

fr_Bs6^{Fo}PK_VNs

ctagaattcTCGAAGAACCAGTTATAGTGTATGAATATGTTGAGGCTATAAGTCTTTATTCTTTACTTTTTGAAAAGGGGACATATGATGATCAAATTAGAAAATCATTACTATC
TTGGGGAAGTAGACTACGTATAGCCCACCAGGTCGTTTCTGCAGTCGTTTTTCTCCATACCGAATTTACTACGCCCGTCATCCvgaaggagaacaactccgaagcAATGTGATTG
TAGATCAGAACAGCGGCGTCGCTAAACTATTGAATTTCTCATTATCTGTATCATTGCCCCGGGGAGAATTGGAAGTAGTAAAAggatcctga

fr_Bs6^{Fo}NPK_Sal_Apa

tcagtcgacATACCTCGCTGTCAACCCTGTGTTCCATGCTCGCACTAAACATATTGAACTTGATTTTCACTTTGTGGGGGAGAAGGTAGTTTCAAGAGACATGAAAGTCTGCTAC
ATAAGTTCTATGGATCGGGTGGCAGATATCATGACTAAAAGTCTTGCTACACGTCGATTTTCTATTTCTCAGGACAAGTTTGCTATGGTTGTACCATTGCTGCACTTACGTGGGG
CTGTAAGGATAAAGGAATTGCCTAATAGATAATGATACTGTTTTGATTTTAAAAATTCAAATGTATAGTTTGCAATTATATTCCTTTATCTAGTGGATATATGATTAGCTTAAAC
TCACATACTAGAGTCCTACTTGGACTCTTCATAGCAGCAACTTTATGTATATATATAGAGGTCCAATCCTCTAAACGTAATATAATATATTCCAGGATTAAATCCTTTCAAATTG
TAACCAAAGAAATAATTGTTTTTATAATTCTAAAATCAACAATAAAAGCTAAGACGTAATTAGATGGGTTGTAGACAATACTCCTTTCATATCACCCAAAATGCTAAAAAGACA
CTAGTATTAAGTGGCAAGATGTTTGCCCCAACCCCGATCTTGACTCTTGACCTTGATTCGAGACTCTATTTTGACCTTGACTTTAGACCCGAGACATGATTTTGGATCTCGACCA
GGGATAAAATTCCCAATCCCATCCCATAATCCATTTCTTGATCCCCGACTCTGACTACTTGGAATTCGTTTCTCGATCCCCTACCTTGACTCGGGATCGTCACCTAACTTGGAAC
CCACTCCTTATCTGATTCTAGACCCAACCCTGTGTTGGGAAACAACAAAATAAAAATGTTAACAAAATATTATAATAATAATATTAACAATACAATAACATAACAGTCACCTAAC
TACTTCTTAACAAGTTAATATATTAATTATACTTGAGTCATGCTAGGCACTGTCCTAAGAAACTATTTCAGCAGTGTGAAATATTTTTTCAGGATTAAACAAACACTTCCATGAT
CAACTAAAGGATTTCTGAGTTAAATAAGTCTTTCCTAATTAACCAGAGTATACATGAATCAACAAAACTATTAATATAAATATCAACAAATTAAACTATAGGTTTACTAAATACT
AACTAAAATCACTAAGGAATATAATATAGAAGGGAAGAGGAAAATGTCAAAAAGAAAGGAGATTGAAAGCAGGAATGTTCCTTCTTTTCTGTAAGGATTGAGAGAGTATTGTAAC
TAAGACTTAAATTCTCAAGTACATCTCTTGATGACTTCTAGAGTAAGGTTTCTCAAGTACATCTCTTGATGACTTCCAGAGCCTTCTCAAGTATATCTCTTGATAACTTTCAAAG
TAAGCCTTCTCAAGTACATTTGTTGATGACTTTCAAAGTAAGTCTTTTAAGTAGCCTCTAAGATACATTTAAAGACATGACTTTTTCAATACACTTCAAATATAAATTATTTTGA
AATAACTAAATAATTTTCCTACACCCTAACCCCACTTTGACCCTGATCCAAGATTGGACTCCCGGCCTCGACCAGGAACCCAATTCTAATCAGTAACCAGCCTCTAAAACCGACC
CAAGAGTTGGATCTCGGGTTGGGATCGGACTCAGATAGAAAAGACATTTTTCTTGAATTTGAGAAGAATAAGTTCACTCAGAAAAATATTTTTCAAAACATTAATTTAACTAACC
AAACATGAAAACATTGGAGTTACAACCTACCAAACACCCATTCTATACTGTTTTTCATGAAGTTCAAATTCTAGTCAAGAGAGACACTGCAGCCAGTGACAATGAAACTCCCCAA
TAACATGAGAGACTCTAGTCAAGACCAAAGTAACGTGGAACAATGCACACCCTCTAGTACACGGACCATCAGGAAACTTCCCAACTAATAGATTCATTCCACGCGCGAGAGATCA
AGAGTTGGATCAGAGAAACCAGACTTATGAATTTATTTGTTGCTATTGCAATTGGTCTTGGACAAGAACAAAATGCATCATTTGATGAGTGCACTACTCACAACCAAAAAACTTT
TTTCCTCTGCCTCCGTAAAAAAGCGGAAGAAAGAGCAGGATTGCTATTTAAATAATGGAAGTGCAGTGCTAGAGGAGATTCTTGCTTTATGCAACGGAAATTGCCGAATTCCCAT
CCGTTACTTCACTGCCACTGAGATAGAAAGGGCGATCAAGCACTCTGAAAAGAAAATAGAACTCGACGAAGGCTATATGGTTACAGGCTCCCTAGACAACCACCTCTTTTTAATT
AGGTTCTCTCTCTATTCCTATGGCGTGCACCGAGATATAGCAATTACAGCTCAGATGAGTCATCTCAAGAATGTGCTGAGACTTATTGGTTGCTGTCTAGAATTTGAAGAACCGG
TTATGGTGTATGAATATATTGAAGGGATATCTCTCGGCGATCTACTTTTCGAAGAGGGTAATCATGATGATCAAACTAGAAATTCACTATTATCTTGGGGAAGTAGATTACGAAT
TGCCAATGAGGTTGCTTCTGCACTCGTCTTTCTCCATACCGAATTTACTACACCTATCATCCACAGAGATATAAAGCCTCACAAAGTGATAATAGATCAGAATAGCAGCATTGCC
AAAATAGTGGACTTCTCATTTTCCATATCATTGCCTTCAGGGGAATTGGAGGTACAAGATCGTGTGTGCGGAACAATCGAGTATTTAGATCCAGAATATGGCTACTGTGGTATTA
TTACTCAAAAAACTGACGTGTACAGCTTTGGAGTTCTTCTCTTTGAGCTATTAACTGGAAAGGAATTGCGTGATATTATGGAATCCAACGTTGAAGAGGGTAATGTTATGGATAG
AGTGGATTCTGCAATTTCAGAAGAGACAATAAATTTTGAAAATTTTCTACCTAACCTCATCGATCGCTATATTAAAGAGGGTAACATAATGGACATAGCAGATAATGCCATTTTA
GAAGAGCATGGAATTGAGATCCAACAGCAATTGCAAGATTACTTGAATCTTGTGAAGAAATGCACAACTTACAACAGAAAAAATAGACCATACATGATTCAAGTTGCAAAGGAAT
TATGCCGAATTCACAAGTGCTTCCATGCCTTCACTCTTGGCCAGATTTAGgggccctag

fr_Bs6^{Fo}N_Sal_Apa

tcagtcgacCTATATAAGATGGGGAAAAAAATTAAACAGCACATCATTTGCATGAAATAATCATGGCTTTATCTGTAGTGGTTTCAAGTTTCAAAGGAAATATAGTTTGTTTGCA
GAGTCATATTATGTAGGGATTTGGACGATAGAAGAGATCTGTAGATAATGAAATTGTCAATTTGGCATACTTAACAGGGAAAAGGTCTTACTAAATGCTAGTTACATCGATCTTT
TCAGTGCTTAATGAAGAAGCCAGGTATCGAGATAGGTCAATTTGAAATTTATGGGTTCGTCATGTTAAATTTCCTCTCTGTTATGATAACAGGGATATTCTCAGATTTTAATACA
GTATAGTTAATTTGCCTGCAAGAAGAAACCTTTTTATTTTTACATTTATCAAAACTTGATGTTGTTACCTTTATTTTTTCCTTCAGAAGATATTTTGTAAGAGGGGTGAAAAAGA
AATTAAAGGAGGGCAAATATGGCAAAGAAGCAAGATGATTGGAAAGTGGGACCTAAATCAGTCCATCTTTCAAATCAAAGGCCATTTATTGTCATTTTGTGTGGAAATTGTCAAA
GTTCAAAAGAGCAGCAGGATCCAGGAAGCTCACAACTTCCTTATCCATTCATTCCCACTAGTGTTGCCTTATTGGATCCAATCCAACGAGTAATGGCCCATTTATTGTCAAAATA
ATGTATTTTTAGACACAATCCTAATATCATTAATTTTTTTAATAAAAGCTTCTCATATTCATATCGGGTAAATATAAGTAGCATTAAACAAAGAAAATCAATTGAAAAACACATC
AAATAAGCCTTGAGGCATCGACGCTGCATCAACAATTCAGATTTCTTTGTATCATTACATCAATAATTGTTTCAATCTATTAATTTCATGATCTAATAAGACTCATTTAGATCAA
AGATGATATCAACTTCTACGTTAGGTGATTTCAGAATCTTCAGTAGAGGTTGGTTAGAATTTTCATTAGCATCTAAAATTTTGTCTAAGAGAAGTTTCATACGCTGTAACTCAGG
CTCTTAGATTACAAAATCTTCCGAGTCAACTTTTTTGAGGTAATTAAATATTATATTAGTGAAACGTTCCACTTGAAGAAATTTTGGGATAGGAATATAATCGTAAAACATTGTC
ACCTAAAACATTAACGGTGAGGTTTGTCAGTAACATTTTGCAGGTGAGGTTTTTTTTGGAGGAAAAAAAATAGTGTGATAGAAAGGTGATTTGACTGTTGTTAAAAAAATTAACG
GTGTTAGGATTATGACTAAAAATACACCACTTAACTAAATAAAAAGGACTATTAGTGCTTTAGTTGAAAGAATATGTATTATTTTGTGTTTAAAACAAAAATAATGTATGATTTT
GGGCCATTACTCTAATCCAACTAGTAAACATGAGATATTTATAGTGTTAGTGAGAAAAGATACTTCAGTATTAGCAATGCAGGTCTTTAGGGAACTCGCAAGGAAGAAACAATCA
TCATCCTCTGCCTCTGCAGAAACGCTGAAGAAAGAGCATGATTATTATTTAGAGAATGGAAGTGCAGTACTAGAGGAGCTTCTTGCTTTATGCGATGGAAATTGCCGAATTCCCA
TCCGTTACTCCACTGCCACTGAGATAGAAAAAGCAATAAAGCACCCTGAAAACAAAATGGAGCTTTCCTATGGGTATATGGTTACAGGATCACTGGACAAACGCCTCGTTTTCAT
TAGGATTTTCCCCGGATATTATAAAAATTACTTTTAAgggccctag

fr_bs6^{61}n_Bs6^{Fo}PK_Sal-Apa

tcagtcgacCTATATAAGATGGGGAAAAAAATTAAACAGCACATCATTTGCATGAAATAATCATGGCTTTATCTGTATTGGTTTCAAGTTTCAAAGGAAATATAGTTTGTTTGCA
GAGTCATATTATGTAGGGATTTGGACGATAGAAGAGATCTGTAGATAATGAAATTGTCAATTTGGCATACTTAACAGGGAAAAGGTCTTACTAAATTCAATTGCTTTTTAAATAC
AACATCTGATATATGAATATTCAGGATATAAATGTTATTCTCTTCGTTTCATAATAAATAAATTATTAAATTTTGACACATATATTAAAAAAAAATCACTAAAAATATGAATTTAAC
ACAATTTTTTATTTTTATCTCAAAAAAAAGTTGATGTAATAATACGTTTTCAAAAGTTAATTGATTGTCAAGTTATAAGAATAATTTTTTTAAAAAAAATTAATGATTCATTTAT
TTTAAAATATCAATAAATATCCTAACAATTTATTTATTTTGAAAAGGAGGAAGTAATTGTGTCACCGTTGAAAATATTGGAAGATGAGATTGTAACTTATCAAGTCATGTTAAAT
TTCCTCTCTGTTATGATAACAGGGATATTCTCAGATTTTAATACAGTAGTAGTTAATTTGCCTGCAAGAAGAAACCTTTTTATTTTTACATTTATCAAAACTTGATGTTGTTACC
TTTATTTTTTCCTTCAGAAGATATTTTGTAAGAGGGGCTGAAAAAGAAATTAAAGGAGGGCAAATATGGCAAAGAAGCAAGATGATTGGAAAGTGGGACCTAAATCAGTACATCTT
TCAAATCAAAGGCCATTTATTGTCATTTTGTGTGGAAATTGTCAAAGTTCAAAAGAGCAGCAGGATCCAGGAAGCTCACAACTTCCTTATCCATTCATTCCCACTAGTGTTGCCT
TATTGGATCTAATCCAACGAGTAATGGCCCATTTATTGTCATTATGTTTTATTTTAGGCATAAAATAATATATATTTTTTCAATTGGTGTATTAATAGTTTTTTTAATTTAAAAA
ATAGTGTATTTTTAGGCACAATCCTAATATCATTAATTTTTTTAACAAAAGCTTCTCAAATTCATATCAGGTAAATATAAGTACCATTAAATAAAGAAAATCAATTGAAAAACAC
ATCAAATAAGCCTTGAGGCATCGACACTGCATCAACAATTCAGATTTCTTTGTATCATTACATCAATAATTGTTTCAATCTATTAATTTCATGATCTGATAAGACTCATTTAGAT

CAAAGATGATATCAACTTCTACGTTAGGTGATTTCAGAATCTTCAGTAGAGGTTAGTTAGAATTTTCATTAGCATCTAAAATTTTGTCTAAGAGAAGCTTCATACGCTGTAACTC
ACGCTCTTAGATTACAAAATCTTCCGAGTCAACTTTTTTGAGGTAATTAAATATTATATTAGTGAAACGTTCCACTTGAAGAAATTTTGGGATAGGAATATAATCGTAAAACATT
GTCATCTAAAACATTAACGGTGAGGTTTGTCAATAACATTTTGCAGGTGAGGTTTTTTTTTGGAGGGAAAAAATAGTGTGATAGAAAGGTGATTTGATTGCTGTTAAAAAAATTA
ACGGTGTTAGGATTATGACTAAAAATACACCACTTAACTAAATAAAAAGGACTATTAGTGCTTTAGTTGAAAGAATATGTATTATTTTGTGTTTAAGACAAAAATAATGTATGAT
TTTGGGCCATTACTCTAATCCAACTAGTAAACATGAGATATTTAAAGTGTTAGTGAGAAAAGATACTTCAGTATTAGCAATGCAGGTCTTTAGGGAACTCGCAAGGAAGAAACAA
TCATCATCCTCTGCCTCTGCAGAAACGCTGAAGAAAGAGCATGATTATTATTTAGAGAATGGAAGTGCAGTACTAAGAGGAGCTTCTTGCTTTATGCGATGGAAATTGCCGAATT
CCCATCCGTTACTTCACTGCCACTGAGATAGAAAAAGCAATAAAGCACCCTGAAAACAAAATGGAGCTTTCCTATGGGTATATGGTTACAGGATCACTGGACAAACGCCTCGTTT
TCATTAGGATTTTCCCGGATATTATAAAAATTACTTTTAATATTTTTCGAGATGTAGCAATTACTGCTCAGATGAGCCATCTCAAAAATGTACTGAGACTTGTCGGTTGCTGTC
TAGAATTCGAAGAACCAGTTATAGTGTATGAATATGTTGAGGCTATAAGTCTTTATTCTTTACTTTTTGAAAAGGGGACATATGATGATCAAATTAGAAAATCATTACTATCTTG
GGGAAGTAGACTACGTATAGCCCACCAGGTCGTTTCTGCAGTCGTTTTTCTCCATACCGAATTTACTACGCCCGTCATCCATCGAGATCTGAAGCCATCGAATGTGATTGTAGAT
CAGAACAGCGGCGTCGCTAAACTATTGAATTTCTCATTATCTGTATCATTGCCCCGGGGAGAATTGGAAGTAGTAAAAGATGTTATGTGCGGAACGTATGGGTACTTAGCTTCAG
AATATGCATCGTCTTGTATTGTTACTCAAAAAATAGACGTCTATAGCTTCGGAGTTGTTCTCCTTCAGCTATTAACTGGATGGAAAATGAGTACTCTACATTTAAAAGATCGCAA
TATGGACTACATAGTGGATCCTTCGGATTCAGATTTTAAGATGATGAACATTGAAGAGATTCATGTAATGGACATAGCAGATCCGGCCATTTTGGAAGAACATGGAATTGCGATT
CAACAACAACTGAAAGATTGCTGGGATCTTGTTAACAAATGCACAAAGTCAAGCGGAAAAGATAGACCATACATGATTGAAGTTGCAAGGGAATTACACCGAATTCACAAGTGCT
TCCGTGCATGCCCTTAGgggccctag

fr_PsK^{751}

tcagtcgacCAGGAGAGCTAGGAGTTGGAAAAAATTTTCAGCATAGAACCATTAAGGCAGAGGATGTAACTAGTCAGATAAAGGAAGAACTTGTACATAAGATGGAGATGCTAGAT
TCCTCAAAAAAAGCTTATAACCGTAAAGTGTGTATTAGGAGACTTCTTTGTATATAGAGTGCTAGTGTCATCTGTAAGTAGTTGATTTTTGAGACTCTCCTCTTAATGTTGCTGT
CGATAGTGAGAGTGCTCTTTAGGTGTATTGATTGAATTGTTAATGATACATTTACATAATTACCAAAAAAAAAAAAATTTGGGAAACAGTGTTAATATGCTAAATGCTAATCACA
TGAATCCTTTCAATCATTTCAGTGATTAAACTAAAATGCTTTAAACACAAGAAAAAGTAATGAATATCCAGGATAAAGGTTACTTGTGTCAGCCGTTAAAAATCTTGGAAGATGG
GATTGTAACTTTTCTACTCATGTTAAATTTCCTCTCTGCTATGATAACAGAGACATTCTCAAATTTTAATATACAAGTTAATTTTGCCCCATTTCAGAGTCATAAGAAACCTTTT
CAGTTTTACATTTAGCAAAACTTGATGTCATCTTTTATTTTTCTTTCAGCAGATATTTTGTAAGAGGAGTGAAAAGAAATTAAAGGAGGGCAAATATGGCAAAGAAGAAAGGATG
ATTGGAAAGTGCACCTACATCAATCATTTTCAATAAAGGGTGTGTTTGGTATGATGGAAAATATTTTCCGGAAAATGTTTTTCGATTTTTCCTTGTTTGGTTGCAACAAAAGTT
TAGGAAAATATTTTTCAAGACAATTTATTTTCCACCATTTGATGGAAAATGACTTCCCTCATGGGCCAAGAGAAGTCATTTTTCAGAAAATGACAAATGTGACTTATGATCTCAT
CCTCACCCCTCTTCCCATCCCAACAACACTTATATATTCACATGACTCAAAAAATTTACATTACTCAAAAATGTTTTTCACTGTGCTTTGCTTTAATTTTTCACTATTTGAAATA
AAAAATAATAAAGTCCGATTTTTTTTCATTTTCAATTCGTTGAATATATTGTTATTTTCATATGCATAGAGGAAGACTTACTTATGTTATCTTTGTTAATTGCATATTTCATTTT
GTTATCGATGTAACTTGTTTCACGAGGTTGAATAAGTTTGTCGTTCTGTTACATTTCTATTAATTGTAGTATCTTAAGATTGTCCTAACAAAATACTGAAAAGTATCTCTTATAT
TTGTTAATTAATAGTTGCTTAAATTTAGTGATCGTAATATTATAGTATTCGATATTGATATTATTTGTTATACTGAAATTAGTTCTTACAAATTATTGAGTTGCTAGAGGCACCG
ATATACTAGTTAACAGATAGTAGTATTTTCTAAAAAATATTTTTTCACTCATCAATCGAACACTAGAAAATATTTTTCTAAAAAATATTCTCTACTCACCAACCAAACACCATAA
AATATTTTTCGAAAAATATTTTTCATTCACCAACCAAACATGAAAAAATAAATAGAAAACTAAGTTATTTTTCAGAAAAATATTTTTCAAAAAAATATTTTTCATGGAAAATATT
TTGGATCGTACCAAACACACCCCAAATGCTCACAACTTGTCAAAAGTTCAAAAAAGCAGCAGGAGCCAGGAAGCTCACAACTTCCTTATCCACTCACTCCCACTAGTAACTTATT
GGATCTAAACCAACTAATAAACATAAGATATTTCAAGTGTTAGTGAGAAAAGATACTTCAATATTAGCAATGCAGTTCATCAAGGGACTCACAAGGAAGAAACAATCATCATCCT
CTGCCTCTGCAGAAACGCGGAAGAAAGAGCATGATTATTATTTAGAGAATGGAAGTGCAGTACTAGAGGAGCTTCTTGCTTTATGCGATGGAAATTGCCGAATTCCCATCCGTTA
CTTCACTGCCACTGATATAGAAAGAGCAATAAAGCACTCTGAAAACAAATTGGATATCGCCGATGGGCATATGGTTGCGGGATCATTGGACAAACGCCTCGTTTTCATTAGGACT
TTCCCTGGATATTTTAGAAACCATTTTAATAATATTTTCCGAGATATAGCAATTACTGCTCAGATGAGTCATCTCAAGAATGTGTTGAGACTTGTCGGTTGCTGTATCGAATTTG
AAGAACCAGTTATGGTGTATGAATATGTTGAGGCAACAAGTCTTTATGCTTTACTTTTTGAAAAGGATAATCATGATGATGAAACTAGAAAATCATTATTATCTTGGGGAAGTAG
ATTACGGATTGCCCACAAGGTCGCTTCGGCAGTCGTTTTTCTACACACTGAATTTACTACACCCATCATCCACAGAGATCTGAAGCCATCAAATGTGATTGTAGATCAGAACAGC
GGTGTTGCAAAACTATTGAACTTCTCATTATCCGTATCATTACCCCAGGGAAAATTGGAAGTAGTAAAAGATGTCGTTTGTGGAACGTATGGGTATGTAGCTCCAGAATATTCGG
AGTGGTGTATGATTACTCAAAAAATAGACGTCTATAGCTTCGGAGTTATTCTCCTACAGCTACTAACTAGACGGAATATGACTACTCTACATTTAAAGGATCGGGATATGTACTA
CATAGTGGATCCTTCAGATTCAGATTTTAAGATGCTAAACATTGAAGAGATTCATGTAATGGACATAGCAGATCCGGCCATTTTGGAAGAACATGGCATTAAAATTCGACAACAA
TTGGAGGACTGCTTGGATCTTGTTAAGAAATGCACGGCTTATAAGGGAGAAGAGAGACCATACATGATTGAAGTTGCAAAGGAATTAAGCCGAATTCACCACTGCTTCCGTGCCC
TCACTCTTTGCCGGAATTAGgggccctag

fr Bs6^{61}N-x DsRed

tcagtcgacCTATATAAGATGGGGAAAAAAATTAAACAGCACATCATTTGCATGAAATAATCATGGCTTTATCTGTATTGGTTTCAAGTTTCAAAGGAAATATAGTTTGTTTGCA
GAGTCATATTATGTAGGGATTTGGACGATAGAAGAGATCTGTAGATAATGAAATTGTCAATTTGGCATACTTAACAGGGAAAAGGTCTTACTAAATTCAATTGCTTTTTAAATAC
AACATCTGATATATGAATATTCAGGATAAATGTTATTCTCTTCGTTTCATAATAAATAAATTATTAAATTTTGACACATATATTAAAAAAAAATCACTAAAAATATGAATTTAAC
ACAATTTTTTATTTTTATCTCAAAAAAAAGTTGATGTAATAATACGTTTTCAAAAGTTAATTGATTGTCAAGTTATAAGAATAATTTTTTTAAAAAAAATTAATGATTCATTTAT
TTTAAAATATCAATAAATATCCTAACAATTTATTTATTTTGAAAAGGAGGAAGTAATTGTGTCACCGTTGAAAATATTGGAAGATGAGATTGTAACTTATCAAGTCATGTTAAAT
TTCCTCTCTGTTATGATAACAGGGATATTCTCAGATTTTAATACAGTAGTAGTTAATTTGCCTGCAAGAAGAAACCTTTTTATTTTTACATTTATCAAAACTTGATGTTGTTACC
TTTATTTTTTCCTTCAGAAGATATTTTGTAAGAGGGCTGAAAAAGAAATTAAAGGAGGGCAAATATGGCAAAGAAGCAAGATGATTGGAAAGTGGGACCTAAATCAGTACATCTT
TCAAATCAAAGGCCATTTATTGTCATTTTGTGTGGAAATTGTCAAAGTTCAAAAGAGCAGCAGGATCCAGGAAGCTCACAACTTCCTTATCCATTCATTCCCACTAGTGTTGCCT
TATTGGATCTAATCCAACGAGTAATGGCCCATTTATTGTCATTATGTTTTATTTTAGGCATAAAATAATATATATTTTTTCAATTGGTGTATTAATAGTTTTTTTAATTTAAAAA
ATAGTGTATTTTTAGGCACAATCCTAATATCATTAATTTTTTTAACAAAAGCTTCTCAAATTCATATCAGGTAAATATAAGTACCATTAAATAAAGAAAATCAATTGAAAAACAC
ATCAAATAAGCCTTGAGGCATCGACACTGCATCAACAATTCAGATTTCTTTGTATCATTACATCAATAATTGTTTCAATCTATTAATTTCATGATCTGATAAGACTCATTTAGAT
CAAAGATGATATCAACTTCTACGTTAGGTGATTTCAGAATCTTCAGTAGAGGTTAGTTAGAATTTTCATTAGCATCTAAAATTTTGTCTAAGAGAAGCTTCATACGCTGTAACTC
ACGCTCTTAGATTACAAAATCTTCCGAGTCAACTTTTTTGAGGTAATTAAATATTATATTAGTGAAACGTTCCACTTGAAGAAATTTTGGGATAGGAATATAATCGTAAAACATT
GTCATCTAAAACATTAACGGTGAGGTTTGTCAATAACATTTTGCAGGTGAGGTTTTTTTTTGGAGGGAAAAAATAGTGTGATAGAAAGGTGATTTGATTGCTGTTAAAAAAATTA
ACGGTGTTAGGATTATGACTAAAAATACACCACTTAACTAAATAAAAAGGACTATTAGTGCTTTAGTTGAAAGAATATGTATTATTTTGTGTTTAAGACAAAAATAATGTATGAT
TTTGGGCCATTACTCTAATCCAACTAGTAAACATGAGATATTTAAAGTGTTAGTGAGAAAAGATACTTCAGTATTAGCAATGCAGGTCTTTAGGGAACTCGCAAGGAAGAAACAA
TCATCATCCTCTGCCTCTGCAGAAACGCTGAAGAAAGAGCATGATTATTATTTAGAGAATGGAAGTGCAGTACTAAGAGGAGCTTCTTGCTTTATGCGATGGAAATTGCCGAATT
CCCATCCGTTACTTCACTGCCACTGAGATAGAAAAAGCAATAAAGCACCCTGAAAACAAAATGGAGCTTTCCTATGGGTATATGGTTACAGGATCACTGGACAAACGCCTCGTTT
TCATTAGGATTTTCCCGGATATTATAAAAATTACTTTTACCCGGATATTATAAAAATTACTTTTAATATTTTTCGAGATGTAGCAATTACTGCTCAGATGAGCCATCTCAAAAAT
GTAATCAAGGAGTTCATGCGCTTCAAGGTGCGCATGGAGGGCACCGTGAACGGCCACGAGTTCGAGATCGAGGGCGAGGGCGAGGGCCGCCCCTACGAGGGCCACAACACCGTGA
AGCTGAAGGTGACCAAGGGCGGCCCCCTGCCCTTCGCCTGGGACATCCTGTCCCCCCAGTTCCAGTACGGCTCCAAGGTGTACGTGAAGCACCCCGCCGACATCCCCGACTACAA
GAAGCTGTCCTTCCCCGAGGGCTTCAAGTGGGAGCGCGTGATGAACTTCGAGGACGGCGGCGTGGTGACCGTGACCCAGGACTCCTCCCTGCAGGACGGCTGCTTCATCTACAAG
GTGAAGTTCATCGGCGTGAACTTCCCCTCCGACGGCCCCGTAATGCAGAAGAAGACCATGGGCTGGGAGGCCTCCACCGAGCGCCTGTACCCCCGCGACGGCGTGCTGAAGGGCG
AGATCCACAAGGCCCTGAAGCTGAAGGACGGCGGCCACTACCTGGTGGAGTTCAAGTCCATCTACATGGCCAAGAAGCCCGTGCAGCTGCCCGGCTACTACTACGTGGACTCCAA
GCTGGACATCACCTCCCACAACGAGGACTACACCATCGTGGAGCAGTACGAGCGCACCGAGGGCCGCCACCACCTGTTCCTGTAGgtcgactga
