# Supplementary material for: Map-based cloning of the susceptibility locus conditioning bs6 resistance in pepper (Capsicum annuum L.) encoding mutant alleles of pseudokinase proteins
Source: Theor Appl Genet. 2026 Oct 20;139(11):308. doi: 10.1007/s00122-026-05407-2 (PMC13683501; doi:10.1007/s00122-026-05407-2)
Supplement: Supplementary file 3 [file 122_2026_5407_MOESM2_ESM.pdf]

Figure S1.

Name of the markers	Genetic Linkage groups of pepper	Number of the F2 plant individuals																Position of the markers (in Mb) on LG6 of CaPe	Distance between markers (in Mb) / recombination events
		P1	P2	P3	P4	P5	P6	P7	P8	P9	P10	P11	P12	P13	P14	P15	P16		
CAMS-855	1	1	2	1	1	2	3	2	1	2	1	1	2	1	2	1	1	Not relevant	Not relevant
EPMS-709	1	3	1	2	2	2	2	2	3	2	2	1	1	2	2	1	1		
Hpms 1–1c	1	1	3	3	3	2	2	2	2	1	2	2	2	2	2	2	2		
Hpms 1–143	2	2	1	3	2	3	2	1	2	3	2	1	2	3	3	3	1		
GPMS-100	2	2	2	1	3	2	2	2	2	2	2	1	1	2	2	2	1		
CAK-24	3	1	2	1	3	2	2	3	2	2	1	3	2	2	3	1	3		
Hpms 1–173	3	1	2	3	3	3	2	2	3	1	2	3	1	1	3	2	2		
CAMS-885	4	1	2	3	3	2	1	2	1	1	2	2	3	2	2	2	2		
CAMS094/4	4	1	3	2	2	1	1	2	1	2	1	3	2	2	2	1	2		
GPMS197	5	2	2	2	3	3	1	1	3	1	2	1	2	2	2	1	2		
GPMS202	5	2	2	2	3	2	3	2	2	2	1	2	2	3	3	3	3		
$XeR6^R/XeR6^S$; *bs6* [61] locus	6	S	R	R	R	R	R	R	R	S	S	S	S	S	S	S	S		
Hpms 1–5	6	1	3	3	3	3	3	3	2	1	2	1	1	2	2	2	2	212.3	15.2 Mb / 4
CAMS-351	6	1	3	2	3	3	3	3	1	2	2	1	1	2	2	2	3	227.5	
EPMS-417	6	2	2	2	3	3	2	3	2	2	2	2	1	2	2	3	3	251.6	24.1 Mb/ 6
CaES-0087	7	3	2	1	3	1	1	3	3	3	1	3	1	3	1	3	3	Not relevant	Not relevant
Hpms 1–166	7	2	2	3	2	3	3	2	2	2	2	3	1	3	1	2	2		
EPMS-642	7	2	2	3	2	3	3	2	2	2	1	3	1	3	1	2	2		
CAK-33	7	2	2	3	2	1	1	1	2	2	1	1	1	1	3	1	3		
CAMS-493	8	2	3	2	2	2	3	3	3	2	2	3	2	3	2	2	2		
CAK-31	8	3	2	2	2	3	2	2	2	3	1	3	2	2	2	1	2		
EPMS-694	8	1	3	3	2	3	2	2	3	3	3	2	2	2	3	3	3		
Hpms 2–24	9	3	3	1	2	2	3	3	2	2	1	2	1	2	2	2	2		
CaES-0091	9	3	3	2	2	3	2	1	3	2	2	2	2	1	2	3	2		
GPMS-159	10	2	3	1	2	2	3	2	2	1	2	2	2	2	3	2	1		
CaES-0192	10	2	3	1	2	2	3	1	2	2	2	2	2	2	3	1	1		
CAMS-117	11	1	3	2	2	1	2	2	1	1	1	3	3	3	1	1	1		
CAMS-647	11	2	1	1	3	1	1	2	1	1	3	1	1	1	2	1	3		
EPMS-716	11	2	1	2	2	3	2	1	2	2	2	2	1	1	3	1	3		
CaES-0048	12	2	2	2	3	2	1	2	3	1	2	2	2	1	2	2	2		
GPMS-166	12	2	1	3	3	2	2	2	3	1	2	2	2	2	2	1	2		
