# Supplementary material for: Map-based cloning of the susceptibility locus conditioning bs6 resistance in pepper (Capsicum annuum L.) encoding mutant alleles of pseudokinase proteins
Source: Theor Appl Genet. 2026 Oct 20;139(11):308. doi: 10.1007/s00122-026-05407-2 (PMC13683501; doi:10.1007/s00122-026-05407-2)
Supplement: Supplementary file 4 [file 122_2026_5407_MOESM3_ESM.pdf]

Figure S2

Distance between markers (Kb)	Position of the markers (Mb) on LG6 of *CaPe*	Name of the markers	P1	P2	P3	P4	P5	P6	P7	P8	P9	P10	P11	P12	P13	P14	P15	P16	P17	P18	P19	P20	P21	P22	P23	P24	P25	P26	P27	P28	P29	P30
3 589 067	205 554 011	m1634	1	3	3	3	3	2	3	2	1	2	2	1	2	2	2	2	3	3	3	3	3	3	2	1	2	2	2	2	1	2
249 635	209 143 078	m1635	1	3	3	3	3	2	3	2	1	2	1	1	2	2	2	2	3	3	3	3	3	3	2	1	2	2	2	2	1	2
1 390 823	209 392 713	m1636	1	3	3	3	3	2	3	2	1	2	1	1	2	2	2	2	3	3	3	3	3	3	2	1	2	2	2	2	1	2
	bs6^{61} locus	XeR6^{R}/XeR6^{S}	s	R	R	R	R	R	R	S	S	S	S	S	S	S	S	S	R	R	R	R	R	R	S	S	S	S	S	S	S	S
213 396	210 783 536	m1652	1	3	3	3	3	3	3	2	1	2	1	1	2	2	2	2	3	3	3	3	3	2	2	1	2	2	2	2	1	2
352 253	210 996 932	m1653	1	3	3	3	3	3	3	2	1	2	1	1	2	2	2	2	3	3	3	3	3	2	2	1	2	2	2	2	1	2
352 253	211 364 215	m1654	1	3	3	3	3	3	3	2	1	2	1	1	2	2	2	2	3	3	3	3	3	2	2	2	2	2	2	2	1	2
584634	211 716 468	m1555	1	3	3	3	3	3	3	2	1	2	1	1	2	2	2	2	3	3	3	3	3	2	2	2	2	2	2	2	1	2
	212 301 102	Hpms 1–5	1	3	3	3	3	3	3	2	1	2	1	1	2	2	2	2	3	3	3	3	3	2	2	2	2	2	2	2	1	2

Distance between markers (Kb)	Position of the markers (Mb) on LG6 of *CaPe*	Name of the markers	P31	P32	P33	P34	P35	P36	P37	P38	P39	P40	P41	P42	P43	P44	P45	P46	P47	P48	P49	P50	P51	P52	P53	P54	P55	P56	P57	P58	P59	P60	P61	P62
3 589 067	205 554 011	m1634	2	2	3	3	2	2	3	2	3	3	3	3	3	3	3	2	2	3	2	2	1	2	1	3	1	2	3	2	3	3	3	3
249 635	209 143 078	m1635	2	3	3	3	2	3	3	2	3	3	3	3	3	3	3	2	3	3	2	1	1	2	1	3	1	2	3	2	3	3	3	3
1 390 823	209 392 713	m1636	2	3	3	3	2	3	3	2	3	3	3	3	3	3	3	2	3	3	2	1	1	2	1	3	1	2	3	2	3	3	3	3
	bs6^{61} locus	XeR6^{R}/XeR6^{S}	S	R	R	R	S	R	R	S	R	R	R	R	R	R	R	S	R	R	S	S	S	S	S	R	S	S	R	S	R	R	R	R
213 396	210 783 536	m1652	2	3	3	3	2	3	3	2	3	3	3	3	3	3	3	2	3	3	2	1	1	2	1	3	1	2	3	2	3	3	3	3
352 253	210 996 932	m1653	2	3	3	3	2	3	3	2	3	3	3	3	3	2	3	2	3	3	2	1	1	2	1	3	1	2	3	2	3	3	3	3
352 253	211 364 215	m1654	2	3	3	3	2	3	3	2	3	3	3	3	3	2	3	2	3	3	2	1	1	2	1	3	1	2	3	2	3	3	3	3
584634	211 716 468	m1555	2	3	3	3	2	3	3	2	3	3	3	3	3	2	3	2	3	3	2	1	1	2	1	3	1	2	3	2	3	3	3	3
	212 301 102	Hpms 1–5	2	3	3	3	2	3	3	2	3	3	3	3	3	2	3	2	3	3	2	1	1	2	1	3	1	2	3	2	3	3	3	3

Distance between markers (Kb)	Position of the markers (Mb) on LG6 of *CaPe*	Name of the markers	P63	P64	P65	P66	P67	P68	P69	P70	P71	P72	P73	P74	P75	P76	P77	P78	P79	P80	P81	P82	P83	P84	P85	P86	P87	P88	P89	P90	P91	P92
3 589 067	205 554 011	m1634	2	2	2	3	3	3	2	1	1	2	3	2	3	2	3	3	2	3	3	3	2	3	1	1	2	2	1	1	1	2
249 635	209 143 078	m1635	2	2	2	3	3	3	2	1	1	2	3	2	3	2	3	3	2	3	3	3	2	3	1	1	2	2	1	1	2	2
1 390 823	209 392 713	m1636	2	2	2	3	3	3	2	1	1	2	3	2	3	2	3	3	2	3	3	3	2	3	1	1	2	2	1	1	2	2
	bs6^{61} locus	XeR6^{R}/XeR6^{S}	S	S	S	R	R	R	S	S	S	S	R	S	R	S	R	R	S	R	R	R	S	R	S	S	S	S	S	S	S	S
213 396	210 783 536	m1652	2	2	2	3	3	3	2	1	1	2	3	2	3	2	3	3	2	3	3	3	2	3	1	1	2	2	1	1	2	2
352 253	210 996 932	m1653	2	2	2	3	3	3	2	1	1	2	3	2	3	2	3	3	2	3	3	3	2	3	1	1	2	2	1	1	2	2
352 253	211 364 215	m1654	2	2	2	3	3	3	2	1	1	2	3	2	3	1	3	3	2	3	3	3	2	3	1	1	2	2	1	2	2	2
584634	211 716 468	m1555	2	2	2	3	3	3	2	1	1	2	3	2	3	1	3	3	2	3	3	3	2	3	1	1	2	2	1	2	2	2
	212 301 102	Hpms 1–5	2	2	2	3	3	2	2	1	1	2	3	2	3	1	3	3		3	3	3	2	3	1	1	2	2	1	2	2	2
