# Supplementary material for: Map-based cloning of the susceptibility locus conditioning bs6 resistance in pepper (Capsicum annuum L.) encoding mutant alleles of pseudokinase proteins
Source: Theor Appl Genet. 2026 Oct 20;139(11):308. doi: 10.1007/s00122-026-05407-2 (PMC13683501; doi:10.1007/s00122-026-05407-2)
Supplement: Supplementary file 5 [file 122_2026_5407_MOESM4_ESM.pdf]

Figure S3. DNA and deduced amino acid sequence of the Bs6^{Fo}A region.

```
        10        20        30        40        50        60        70        80        90       100       110       120
         |         |         |         |         |         |         |         |         |         |         |         |
tattgggtattgaagtttgtgctgcctcgcatggtctattattatgtcaatccaaatatatcagagatttattaactagagcttctatggaaggtgcaaagtctatatcgacacccatgg  120
|---------- pr1637_F ---------→
taattggtcttcaactttcccagcatggtagcccaatgtttgatcaaccggaactttatcggagtattgttggagcattacaatatatcacaattacccgacctgatgtctcctttgctg  240

ttaacaagttgtgtcagtttatgcatgatcctcacgaaatgcattatacagctatgaagaggttgcttagatatctaaaaggtacaattgatcttggtttactgttgcgttcatctgctt  360

ccttcacacttactggtttctcggatgctgactgggctggcttcccagatgaacgacgttctactcatggcttctgcatatttttgggtgccaacctggtgtcatggagctccaaaaaaa  480

caacgtatagttgctcgctctagcacagaatcagagtatcaaggtcttgctacaaccacagctgaaataactcggctggaaaatctacttaaagaactccaaattgtgcttccagctgct  600

ccaaaagtatggtgtgataatttatcagccacatacctcgctgtcaaccctgtgttccatgctcgcactaaacatattgaacttgattttcactttgtgggggagaaggtagtttcaaga  720

gacatgaaagtctgctacataagttctatggatcgggtggcagatatcatgactaaaagtcttgctacacgtcgattttctatttctcaggacaagtttgctatggttgtaccattgctg  840

cacttacgtggggctgtaaggataaaggaattgcctaatagataatgatactgttttgattttaaaaattcaaatgtatagtttgcaattatattcctttatctagtggatatatgatta  960

gcttaaactcacatactagagtcctacttggactcttcatagcagcaactttatgtatatatatagaggtccaatcctctaaacgtaatataatatattccaggattaaatcctttcaaa 1080

ttgtaaccaaAGAAATAATTGTTTTTTATAATTCTAAAATCAACAATAAAAGCTAAGACGTAATTAGATGGGTTGTAGACAATACTCCTTTCATATCACCCAAAATGCTAAAAAGACACT 1200

AGTATTAAGTGGCAAGATGTTTGCCCCAACCCCGATCTTGACTCTTGACCTTGATTCGAGACTCTATTTTGACCTTGACTTTAGACCCGAGACATGATTTTGGATCTCGACCAGGGATAA 1320

AATTCCCAATCCCATCCCATAATCCATTTCTTGATCCCCGACTCTGACTACTTGGAATTCGTTTCTCGATCCCCTACCTTGACTCGGGATCGTCACCTAACTTggaacccactccttatc 1440

tgattctagacccaaccctgtgttgggaaacaacaaaataaaaatgttaacaaaatattataataataatattaacaatacaataacataacagtcacctaactacttcttaacaagtta 1560

atatattaattatacttgagtcatgctaggcactgtcctaagaaactatttcagcagtgtgaaatattttttcaggattaaacaaacacttccatgatcaactaaaggatttctgagtta 1680

aataagtctttcctaattaaccagagtatacatgaatcaacaaaactattaatataaatatcaacaaattaaactataggtttactaaatactaactaaaatcactaaggaatataatat 1800

agaagGGAAGAGGAAAATGTCAAAAAGAAAGGAGATTGAAAGCAGGAATGTTCCTTCTTTTCTGTAAGGATTGAGAGAGTATTGTAACTAAGACTTAAATTCTCAAGTACATCTCTTGAT 1920

GACTTCTAGAGTAAGGTTTCTCAAGTACATCTCTTGATGACTTCCAGAGCCTTCTCAAGTATATCTCTTGATAACTTTCAAAGTAAGCCTTCTCAAGTACATTTGTTGATGACTTTCAAA 2040

GTAAGTCTTTTAAGTAGCCTCTAAGATACATTTAAAGACATGACTTTTTCAATACACTTCAAATATAAATTATTTTGAAATAACTAAATAATTTTCCTACACCCTAACCCCACTTTGACC 2160

CTGATCCAAGATTGGACTCCCGGCCTCGACCAGGAACCCAATTCTAATCAGTAACCAGCCTCTAAAACCGACCCAAGAGTTGGATCTCGGGTTGGGATCGGACTCAGATAGAAAAGACAT 2280

TTTTCTTGAATTTGAGAAGAATAAGTTCACTCAGAAAAATATTTTTCAAAACATTAATTTAACTAACCAAACATGAAAACATTGGAGTTACAACCTACCAAACACCCATTCTATACTGTT 2400

TTTCATGAAGTTCAAATTCTAGTCAAGAGAGACACTGCAGCCAGTGACAATGAAACTCCCCAATAACATGAGAGACTCTAGTCAAGACCAAAGTAACGTGGAACAATGCACACCCTCTAG 2520

TACACGGACCATCAGGAAACTTCCCAACTAATAGATTCATTCCACGCGCGAGAGATCAAGAGTTGGATCAGAGAAACCAGACTTATGAATTTATTTGTTGCTATTGCAATTGGTCTTGGA 2640

CAAGAACAAAATGCATCATTTGATGAGTGCACTACTCACAACCAAAAAACTTTTTTCCTCTGCCTCCGTAAAAAAGCGGAAGAAAGAGCAGGATTGCTATTTAAATAATGGAAGTGCAGT 2760
pBs6^FoNPK -> M  H  H  L  M  S  A  L  L  T  T  K  K  L  F  S  S  A  S  V  K  K  R  K  K  E  Q  D  C  Y  L  N  N  G  S  A  V
GCTAGAGGAGATTCTTGCTTTATGCAACGGAAATTGCCGAATTCCCATCCGTTACTTCACTGCCACTGAGATAGAAAGGGCGATCAAGCACTCTGAAAAGAAAATAGAACTCGACGAAGG 2880
 L  E  E  I  L  A  L  C  N  G  N  C  R  I  P  I  R  Y  F  T  A  T  E  I  E  R  A  I  K  H  S  E  K  K  I  E  L  D  E  G

CTATATGGTTACAGGCTCCCTAGACAACCACCTCTTTTTAATTAGGTTCTCTCTCTATTCCTATGGCGTGCACCGAGATATAGCAATTACAGCTCAGATGAGTCATCTCAAGAATGTGCT 3000
 Y  M  V  T  G  S  L  D  N  H  L  F  L  I  R  F  S  L  Y  S  Y  G  V  H  R  D  I  A  I  T  A  Q  M  S  H  L  K  N  V  L

GAGACTTATTGGTTGCTGTCTAGAATTTGAAGAACCGGTTATGGTGTATGAATATATTGAAGGGATATCTCTCGGCGATCTACTTTTCGAAGAGGGTAATCATGATGATCAAACTAGAAA 3120
 R  L  I  G  C  C  L  E  F  E  E  P  V  M  V  Y  E  Y  I  E  G  I  S  L  G  D  L  L  F  E  E  G  N  H  D  D  Q  T  R  N
TTCACTATTATCTTGGGGAAGTAGATTACGAATTGCCAATGAGGTTGCTTCTGCACTCGTCTTTCTCCATACCGAATTTACTACACCTATCATCCACAGAGATATAAAGCCTCACAAAGT 3240
 S  L  L  S  W  G  S  R  L  R  I  A  N  E  V  A  S  A  L  V  F  L  H  T  E  F  T  T  P  I  I  H  R  D  I  K  P  H  K  V

GATAATAGATCAGAATAGCAGCATTGCCAAAATAGTGGACTTCTCATTTTCCATATCATTGCCTTCAGGGGAATTGGAGGTACAAGATCGTGTGTGCGGAACAATCGAGTATTTAGATCC 3360
 I  I  D  Q  N  S  S  I  A  K  I  V  D  F  S  F  S  I  S  L  P  S  G  E  L  E  V  Q  D  R  V  C  G  T  I  E  Y  L  D  P

AGAATATGGCTACTGTGGTATTATTACTCAAAAAACTGACGTGTACAGCTTTGGAGTTCTTCTCTTTGAGCTATTAACTGGAAAGGAATTGCGTGATATTATGGAATCCAACGTTGAAGA 3480
 E  Y  G  Y  C  G  I  I  T  Q  K  T  D  V  Y  S  F  G  V  L  L  F  E  L  L  T  G  K  E  L  R  D  I  M  E  S  N  V  E  E

GGGTAATGTTATGGATAGAGTGGATTCTGCAATTTCAGAAGAGACAATAAATTTTGAAAATTTTCTACCTAACCTCATCGATCGCTATATTAAAGAGGGTAACATAATGGACATAGCAGA 3600
 G  N  V  M  D  R  V  D  S  A  I  S  E  E  T  I  N  F  E  N  F  L  P  N  L  I  D  R  Y  I  K  E  G  N  I  M  D  I  A  D

TAATGCCATTTTAGAAGAGCATGGAATTGAGATCCAACAGCAATTGCAAGATTACTTGAATCTTGTGAAGAAATGCACAACTTACAACAGAAAAAATAGACCATACATGATTCAAGTTGC 3720
 N  A  I  L  E  E  H  G  I  E  I  Q  Q  Q  L  Q  D  Y  L  N  L  V  K  K  C  T  T  Y  N  R  K  N  R  P  Y  M  I  Q  V  A

AAAGGAATTATGCCGAATTCACAAGTGCTTCCATGCCTTCACTCTTGGCCAGATTTAGCTATATAAGATGGGGAAAAAAATTAAACAGCACATCATTTGCATGAAATAATCATGGCTTTA 3840
 K  E  L  C  R  I  H  K  C  F  H  A  F  T  L  G  Q  I  *
TCTGTAGTGGTTTCAAGTTTCAAAGGAAATATAGTTTGTTTGCAGAGTCATATTATGTAGGGATTTGGACGATAGAAGAGATCTGTAGATAATGAAATTGTCAATTTGGCATACTTAACA 3960

GGGAAAAGGTCTTACTAAATGCTAGTTACATCGATCTTTTCAGTGCTTAATGAAGAAGCCAGGTATCGAGATAGGTCAATTTGAAATTTATGGGTTCGTCATGTTAAATTTCCTCTCTGT 4080

TATGATAACAGGGATATTCTCAGATTTTAATACAGTATAGTTAATTTGCCTGCAAGAAGAAACCTTTTTATTTTTACATTTATCAAAACTTGATGTTGTTACCTTTATTTTTTCCTTCAG 4200

AAGATATTTTGTAAGAGGGGTGAAAAAGAAATTAAAGGAGGGCAAATATGGCAAAGAAGCAAGATGATTGGAAAGTGGGACCTAAATCAGTCCATCTTTCAAATCAAAGGCCATTTATTG 4320

TCATTTTGTGTGGAAATTGTCAAAGTTCAAAAGAGCAGCAGGATCCAGGAAGCTCACAACTTCCTTATCCATTCATTCCCACTAGTGTTGCCTTATTGGATCCAATCCAACGAGTAATGG 4440

CCCATTTATTGTCAAATAATGTATTTTTAgacacaatcctaatatcattaatttttttaataaaagcttctcatattcatatcgggtaaatataagtagcattaaacaaagaaaatcaat 4560

tgaaaaacacatcaaataagccttgaggcatcgacgctgcatcaacaattcagatttctttgtatcattacatcaataattgtttcaatctattaatttcatgatctaataagactcatt 4680

tagatcaaagatgatatcaacttctacgttaggtgatttcagaatcttcagtagaggttggttagaattttcattagcatctaaaattttgtctaagagaagtttcatacgctgtaactc 4800

aggctcttagattacaaaatcttccgagtcaactttttttgaggtaattaaatattatattagtgaaacgttccacttgaagaaattttgggataggaatataatcgtaaaacattgtcac 4920

ctaaaacattaacggtgaggtttgtcagtaacattttgcaggtgaggtttttttttggaggaaaaaaatagtgtgatagaaaggtgatttgactgttgttaaaaaaattaacggtgttag 5040

gattatgactaaaaatacaccacttaactaaataaaaaggactattagtgctttagttgaaagaatatgtattattttgtgtttaaaaCAAAAATAATGTATGATTTTGGGCCATTACTC 5160

TAATCCAACTAGTAAACATGAGATATTTATAGTGTTAGTGAGAAAAGATACTTCAGTATTAGCAATGCAGGTCTTTAGGGAACTCGCAAGGAAGAAACAATCATCATCCTCTGCCTCTGC 5280
                                                  Bs6^FoN ->    M  Q  V  F  R  E  L  A  R  K  K  Q  S  S  S  S  S  A  S  A
AGAAACGCTGAAGAAAGAGCATGATTATTATTTAGAGAATGGAAGTGCAGTACTAGAGGAGCTTCTTGCTTTATGCGATGGAAATTGCCGAATTCCCATCCGTTACTCCACTGCCACTGA 5400
 E  T  L  K  K  E  H  D  Y  Y  L  E  N  G  S  A  V  L  E  E  L  L  A  L  C  D  G  N  C  R  I  P  I  R  Y  S  T  A  T  E

GATAGAAAAAGCAATAAAGCACCCTGAAAACAAAATGGAGCTTTCCTATGGGTATATGGTTACAGGATCACTGGACAAACGCCTCGTTTTCATTAGGATTTTCCCCGGATATTATAAAAA 5520
 I  E  K  A  I  K  H  P  E  N  K  M  E  L  S  Y  G  Y  M  V  T  G  S  L  D  K  R  L  V  F  I  R  I  F  P  G  Y  Y  K  N*

TTACTTTTAATATTTTTCGAGATGTAGCAATTACTGCTCAGATGAGCCATCTCAAAAATGTACTGAGACTTGTCGGTTGCTGTCTAGAATTCGAAGAACCAGTTATAGTGTATGAATATG 5640
 Y  F  *                          Bs6^FoPK -> M  S  H  L  K  N  V  L  R  L  V  G  C  C  L  E  F  E  E  P  V  I  V  Y  E  Y
TTGAGGCTATAAGTCTTTATTCTTTACTTTTTGAAAAGGGGACATATGATGATCAAATTAGAAAATCATTACTATCTTGGGGAAGTAGACTACGTATAGCCCACCAGGTCGTTTCTGCAG 5760
V  E  A  I  S  L  Y  S  L  L  F  E  K  G  T  Y  D  D  Q  I  R  K  S  L  L  S  W  G  S  R  L  R  I  A  H  Q  V  V  S  A

TCGTTTTTCTCCATACCGAATTTACTACGCCCGTCATCCATCGAGATCTGAAGCCATCGAATGTGATTGTAGATCAGAACAGCGGCGTCGCTAAACTATTGAATTTCTCATTATCTGTAT 5880
V  V  F  L  H  T  E  F  T  T  P  V  I  H  R  D  L  K  P  S  N  V  I  V  D  Q  N  S  G  V  A  K  L  L  N  F  S  L  S  V

CATTGCCCCGGGGAGAATTGGAAGTAGTAAAAGATGTTATGTGCGGAACGTATGGGTACTTAGCTTCAGAATATGCATCGTCTTGTATTGTTACTCAAAAAATAGACGTCTATAGCTTCG 6000
S  L  P  R  G  E  L  E  V  V  K  D  V  M  C  G  T  Y  G  Y  L  A  S  E  Y  A  S  S  C  I  V  T  Q  K  I  D  V  Y  S  F

GAGTTGTTCTCCTTCAGCTATTAACTGGATGGAAAATGAGTACTCTACATTTAAAAGATCGCAATATGGACTACATAGTGGATCCTTCGGATTCAGATTTTAAGATGATGAACATTGAAG 6120
G  V  V  L  L  Q  L  L  T  G  W  K  M  S  T  L  H  L  K  D  R  N  M  D  Y  I  V  D  P  S  D  S  D  F  K  M  M  N  I  E

AGATTCATGTAATGGACATAGCAGATCCGGCCATTTTGGAAGAACATGGAATTGCGATTCAACAACAACTGAAAGATTGCTGGGATCTTGTTAACAAATGCACAAAGTCAAGCGGAAAAG 6240
E  I  H  V  M  D  I  A  D  P  A  I  L  E  E  H  G  I  A  I  Q  Q  Q  L  K  D  C  W  D  L  V  N  K  C  T  K  S  S  G  K

ATAGACCATACATGATTGAAGTTGCAAGGGAATTACACCGAATTCACAAGTGCTTCCGTGCATGCCCTTAGTCTTGCCCACAATTAGTACAATAAGATGGCCAAAAAAAAAAAAAATCAAG 6360
D  R  P  Y  M  I  E  V  A  R  E  L  H  R  I  H  K  C  F  R  A  C  P  *

TGCACAAGAATTAAGCTTAGCTTTATTTGTGCGGTCATATTATGTCGGATAGTAAAAAAGATCAGCACATAATGAAATTGTCAATTTTTTTTTTTAATTTGCGACATCATTTTCATTTTT 6480

AACTTGGCtcatatatgaaaaatataattttctttttatttttcattttaacaaatcaatagagatttaatttattattttatattaatattactctatcattaagtaattattcatag 6600

gattttcaaagaaaaatttaataaaacaaccccctaaagtgtcaaatcaataattactaagtaaaaatttgtacggtggcaacgaaagcgacggccgaaatattgagaagtgacgctagag 6720

caaatgcaaacttccttatagagcggatcttttgtcaacttgagtggaattaacgctgtgtttggtatgatgaaaaatattttcctgaaaaatattttcatcaaaaataaattgattata 6840

tttgattggtgagtaaaaaatatttttcaaaaaatagtgtttgtttggtaaatagaaaatatattttgtagaaaaatatcttttagtgttgattgatgaacaaacaaaatattatttgaa 6960

aaatactactaaattgttaactaacatatcattgcctctaataactcaacaatttgtaggaactaatttaagtatagcaatcaatacgaatattatgtaccaaaatattacaagtcaata 7080

aaattaaagcaactactaattatataatccaagtttcctaacaatattccctaaaattaagggtgttaagtgggccggccgggtcaactcggaatcgtcctgttaagtttagctgggttg 7200

tgggctgggctggatcgggccgggttgtagttaagttggccgggtcgggtccgggttcaacagtaataaaaataactctgaatcggcccacttagcccaacccagtct 7308
                                                                                                  <------- prBs6A_R -------|
```
