# Supplementary material for: Map-based cloning of the susceptibility locus conditioning bs6 resistance in pepper (Capsicum annuum L.) encoding mutant alleles of pseudokinase proteins
Source: Theor Appl Genet. 2026 Oct 20;139(11):308. doi: 10.1007/s00122-026-05407-2 (PMC13683501; doi:10.1007/s00122-026-05407-2)
Supplement: Supplementary file 6 [file 122_2026_5407_MOESM5_ESM.pdf]

Figure S4. DNA and dediced amino acid sequence of the bs6[61]A region.

```
        10        20        30        40        50        60        70        80        90       100       110       120
         |         |         |         |         |         |         |         |         |         |         |         |
tattgggtattgaagtttgtgctgcctcgcatggtctattattatgtcaatccaaatatatcagagatttattaactagagcttttatggaaggtgcaaagtctatatcgacacccatgg  120
|---------- pr1637_F --------->

taattggtcttcaactttcccagcatggtagcccaatgtttgatcaaccggaactttatcggagtattgttggagcattacaatatatcacaattacccgacctgatgtctcctttgctg  240

ttaacaagttgtgtcagtttatgcatgatcctcgcgaaatgcattatacagctatgaagaggttgcttagatatctaaaaggtacaattgatcttggtttactgttgcgttcatctgctt  360

ccttcacacttactggtttctcggatgctgactgggctggcttcccagatgaacgacgttccactcatggcttctacatattttttgggtgccaacctggtgtcatggagctccaaaaaaa  480

caacgtatagttgctcgctctagcacagaatcagagtatcgaggtcttgctacaaccacagctgaaataactcggctgcaaaatctacttaaagaactccaaattgtgcttccagctgct  600

ccaaaagtatggtgtggaaaatttatcagccacatacctcgctgtcaaccctgtgttccatgctcgcactaaacatattgaacttgattttcactttgtgggggagaaggtagtttcaaga  720

gacatgaaagtctgctacataagttctatggatcaggtggcagatatcatgactaaaagtcttgctacacgtcggttttctatttctcaggacaagtttgctatggttgtaccattgctg  840

cacttacgtggggctgtaaggataaaggaattgcctaatagataatgatactgttttgattttaaaatttcaaatgtatagtttgcaattatattcctttatctagtggatatatgatta  960

gcttaaactcacatactagagtcctacttggactcttcatagcagcaactttatgtatatatatagaggtccaatcctctaaacgtaatataatatattccaggattaaatcctttcaaa 1080

ttgtaaccaaagaaataattgttttttataattctaaaatcaacaataaaagctaagacgtaattagatgggttgtagacaatactcctttcatatcacccaaaatgctaaaaagacact 1200

agtattaagtggcaagatgtttgccccaaccccgatcttgactcttgaccttgattcgagactctattttgaccttgacttagacccGAGACATGATTTTGGATCTCGACCAGGGATAAA 1320

ATTCCCAATCCCATCCCATAATCCATTTCTTGATCCCCGACTCTGACTACTTGGAATTCGTTTCTCGATCCCCTACCTTGACTCGGGATCGTCACCTAACTTGGAACCCACTCCTTATCT 1440

GATTCTAGacccaaccctgtgttgggaaacaacaaaataaaaatgttaacaaaatattataataataatattaacaatacaataacataacagtcacctaactacttcttaacaagttaa 1560

tatattaattatacttgagtcatgctaggcactgtcctaagaaactatttcagcagtgtgaaatattttttcaggattaaacaaacacttccatgatcaactaaaggatttctgagttaa 1680

ataagtctttcctaattaaccagagtatacatgaatcaacaaaactattaatataaatatcaacaaattaaactataggtttactaaatactaactaaaatcactaaggaatataatata 1800

gaagggaagaggaaaatgtcaaaaagaaaagagattgaaagcaggaatgttccttctTTTCTGTAAGGATTGAGAGAGTATTGTAACTAAGACTTAAATTCTCAAGTACATCTCTTGATG 1920

ACTTCTAGAGTAAGGTTTCTCAAGTACATCTCTTGATGACTTCCAGAGCTTTCTCAAGTATATCTCTTGATAACTTTCAAAGTAAGCCTTCTCAAGTACATTTGTTGATGACTTTCAAAG 2040

TAAGCCTTTTAAGTAGCTTCTAAGATACATTTAAAGACATGACTTTTTCAATACACTTCAAATATAAATTATTTTGAAATAACTAAATAATTTTCCTACACCCTAACCCCACTTTGACCC 2160

TGATCCAAGATTGGACTCCCGGCCTCGACCAGGAACCCAATTCCAATCAGTAACCAGCCTCTAAAACCGACCCAAGAGTTGGATCTCGGGTTGGGATCGGACTCAGATAGAAAAGACATT 2280

TTTCTTGAATTTGAGAAGAATAAGTTCACTCAGAAAAATATTTTTCAAAACATTAATTTAACTAACCAAACATGAAAACATTGGAGTTCCAACCTACCAAACACCCATTCTATACTGTTT 2400

TTCATGAAGTTCAAATTCTAGTCAAGAGAGACACTACAGCCAGTGACAATGAAACTCCCCAATAACTACGTGAGAGACTCTAGTCAAGACCAAACTAACGTGGAACAATGCACACCCTCT 2520

AGTACACGGACCATCAGGAAACTTCCCAACTAAAAGATTCATTCCACGCGCGAGAGATCAAGAGTTGGATCAGAGAAACCAGACTTATGAATTTATTTGTTGCTATTGCAATTGGTCTTG 2640

GACAAGAACAAAATGCATCATTTGATGAGTGCACTACTCACAACCAAAAAACTTTTTTCCTCTGCCTCCGTAAAAAAGCGGAAGAAAGAGCAGGATTGCTATTTAAATAATGGAAGTGCA 2760
 bs6[61]npk >  M  H  H  L  M  S  A  L  L  T  T  K  K  L  F  S  S  A  S  V  K  K  R  K  K  E  Q  D  C  Y  L  N  N  G  S  A

GTGCTAGAGGAGATTCTTGCTTTATGCAACGGAAATTGCCGAATTCCCATCCGTTACTTCACTGCCACTGAGATAGAAAGGGCGATCAAGCACTCTGAAAAGAAAATAGAACTCGACGAA 2880
 V  L  E  E  I  L  A  L  C  N  G  N  C  R  I  P  I  R  Y  F  T  A  T  E  I  E  R  A  I  K  H  S  E  K  K  I  E  L  D  E

GGCTATATGGTTACAGGCTCCCTAGAAAACCACCTCTTTTTAATTAGGTTCTCTCTCTATTCCTATGGCGTGCACCGAGATATTAGCAATTACAGCTCAGATGAGTCATCTCAAGAATGT 3000
 G  Y  M  V  T  G  S  L  E  N  H  L  F  L  I  R  F  S  L  Y  S  Y  G  V  H  R  D  I  S  N  Y  S  S  D  E  S  S  Q  E  C

GCTGAGACTTATTGGTTGCTGTCTAGAATTTGAAGAACCGGTTATGGTGTATGAATATATTGAAGGGATATCTCTCGGCGATCTACTTTTCGAAGAGGGTAATCATGATGATCAAACTAG 3120
 A  E  T  Y  W  L  L  S  R  I  *

AAAATCACTATTATCTTGGGGAAGTAGATTACGAATTGCCAATGAGGTTGCTTCTGCACTCGTCTTTCTCCATACCGAATTTACTACACCTATCATCCACAGAGATATAAAGCCTCACAA 3240

AGTGATAATAGATCAGAATAGCAGCATTGCCAAAATAGTGGACTTCTCATTTTCCATATCATTGCCTTCAGGGGAATTGGAGGTACAAGATCGTGTGTGCGGAACAATCGAGTATTTAGA 3360

TCCAGAATATGGCTACTGTGGTATTATTACTCAAAAAACTGACGTGTACAGCTTTGGAGTTCTTCTCTTTGAGCTATTAACTGGAAAGGAATTGCGTGATATTATGGAATCCAACGTTGA 3480

AGAGGGTAATGTTATGGATAGAGTGGATTCTGCAATTTCAGAAGAGACAATAAATTTTGAAAATTTTCTACCTAACCTCATCGATCGCTATATTAAAGAGGGTAATATAATGGACATAGC 3600

AGATAATGCCATTTTAGAAGAGCATGGAATTGAGATCCAACAGCAATTGCAAGATTACTTGAATCTTGTGAAGAAATGCACAACTTACAACAGAAAAAATAGACCATACATGATTCAAGT 3720

TGCAAAGGAATTATGCCGAATTCACAAGTGCTTCCATGCCTTCACTCTTGGCCAGATTTAGCTATATAAGATGGGGAAAAAAATTAAACAGCACATCATTTGCATGAAATAATCATGGCT 3840

TTATCTGTATTGGTTTCAAGTTTCAAAGGAAATATAGTTTGTTTGCAGAGTCATATTATGTAGGGATTTGGACGATAGAAGAGATCTGTAGATAATGAAATTGTCAATTTGGCATACTTA 3960

ACAGGGAAAAGgtcttactaaattcaattgctttttaaatacaacatctgatatatgaatattcaggataaatgttattctcttcgtttcataataaataaattattaaattttgacaca 4080

tatattaaaaaaaaatcactaaaaatatgaatttaacacaatttttttatttttatctcaaaaaaaaagttgatgtaataatacgttttcaaaagttaattgattgtcaagttataagaata 4200

atttttttaaaaaaaattaatgattcatttattttaaaatatcaataaatatcctaacaatttatttattttgaaaaggaggaagtaattgtgtcaccgttgaaaatattggaagatgag 4320

attgtaacttatcaagtcatgttaaatttcctctctgttatgataacagggatattctcagattttaatacagtagtagttaatttgcctgcaagaagaaaccttttttatttttacattt 4440

atcaaaacttgatgttgttacctttattttttccttcagaagatattttgtaagagggctgaaaaagaaattaaaggagggcaaatatggcaaagaagcaagatgattggaaagtgggac 4560

ctaaatcagtacatctttcaaatcaaaggccatttattgtcattttgtgtggaaattgtcaaagttcaaaagagcagcaggatccaggaagctcacaacttccttatccattcattccca 4680

ctagtgttgccttattggatctaatccaacgagtaatggcccatttattgtcattatgttttattttaggcataaaataatatatatttttttcaattggtgtattaatagttttttttaat 4800

ttaaaaaatagtgtatttttaggcacaatcctaatatcattaattttttttaacaaaagcttctcaaattcatatcaggtaaatataagtaccattaaataaagaaaatcaattgaaaaac 4920

acatcaaataagccttgaggcatcgacactgcatcaacaattcagatttctttgtatcattacatcaataattgtttcaatctattaatttcatgatctgataagactcatttagatcaa 5040

agatgatatcaacttctacgttaggtgatttcagaatcttcagtagaggttagttagaattttcattagcatctaaaattttgtctaagagaagcttcatacgctgtaactcacgctctt 5160

agattacaaaatcttccgagtcaactttttttgaggtaattaaatattatattagtgaaacgttccacttgaagaaattttgggataggaatataatcgtaaaacattgtcatctaaaaca 5280

ttaacggtgaggtttgtcaataacattttgcaggtgaggtttttttttggagggaaaaaaatagtgtgatagaaaggtgatttgattgctgttaaaaaaaattaacggtgttaggattatga 5400

ctaaaaatacaccacttaactaaataaaaaggactattagtgctttagttgaaagaatatgtattattttgtgtttaagacaaaaataatgtatgattttgggccattactctaatccaa 5520

ctagtaaacatgagatatttaaagtgttagtgagaaaagatacttcagtattagcaATGCAGGTCTTTAGGGAACTCGCAAGGAAGAAACAATCATCATCCTCTGCCTCTGCAGAAACGC 5640
                                                   bs6[61]n ->  M  Q  V  F  R  E  L  A  R  K  K  Q  S  S  S  S  A  S  A  E  T

TGAAGAAAGAGCATGATTATTATTTAGAGAATGGAAGTGCAGTACTAAGAGGAGCTTCTTGCTTTATGCGATGGAAATTGCCGAATTCCCATCCGTTACTTCACTGCCACTGAGATAGAA 5760
L  K  K  E  H  D  Y  Y  L  E  N  G  S  A  V  L  R  G  A  S  C  F  M  R  W  K  L  P  N  S  H  P  L  L  H  C  H  *

AAAGCAATAAAGCACCCTGAAAACAAAATGGAGCTTTCCTATGGGTATATGGTTACAGGATCACTGGACAAACGCCTCGTTTTCATTAGGATTTTCCCCGGATATTATAAAAATTACTTT 5880

TAATATTTTTCGAGATGTAGCAATTACTGCTCAGATGAGCCATCTCAAAAATGTACTGAGACTTGTCGGTTGCTGTCTAGAATTCGAAGAACCAGTTATAGTGTATGAATATGTTGAGGC 6000
                               bs6[61]pk ->  M  S  H  L  K  N  V  L  R  L  V  G  C  C  L  E  F  E  E  P  V  I  V  Y  E  Y  V  E  A

TATAAGTCTTTATTCTTTACTTTTTGAAAAGGGGACATATGATGATCAAATTAGAAAATCATTACTATCTTGGGGAAGTAGACTACGTATAGCCCACCAGGTCGTTTCTGCAGTCGTTTT 6120
 I  S  L  Y  S  L  L  F  E  K  G  T  Y  D  D  Q  I  R  K  S  L  L  S  W  G  S  R  L  R  I  A  H  Q  V  V  S  A  V  V  F

TCTCCATACCGAATTTACTACGCCCGTCATCCATCGAGATCTGAAGCCATCGAATGTGATTGTAGATCAGAACAGCGGCGTCGCTAAACTATTGAATTTCTCATTATCTGTATCATTGCC 6240
 L  H  T  E  F  T  T  P  V  I  H  R  D  L  K  P  S  N  V  I  V  D  Q  N  S  G  V  A  K  L  L  N  F  S  L  S  V  S  L  P

CCGGGGAGAATTGGAAGTAGTAAAAGATGTTATGTGCGGAACGTATGGGTACTTAGCTTCAGAATATGCAGCGTCTTGTATTGTTACTCAAAAAATAGACGTCTATAGCTTCGGAGTTGT 6360
 R  G  E  L  E  V  V  K  D  V  M  C  G  T  Y  G  Y  L  A  S  E  Y  A  A  S  C  I  V  T  Q  K  I  D  V  Y  S  F  G  V  V

TCTCCTTCAGCTATTAACTGGATGGAAAATGAGTACTCTACATTTAAAAGATCGCAATATGGACTACATAGTGGATCCTTCGGATTCAGATTTTAAGATGATGAACATTGAAGAGATTCA 6480
 L  L  Q  L  L  T  G  W  K  M  S  T  L  H  L  K  D  R  N  M  D  Y  I  V  D  P  S  D  S  D  F  K  M  M  N  I  E  E  I  H

TGTAATGGACATAGCAGATCCGGCCATTTTGGAAGAACATGGAATTGCGATTCAACAACAACTGAAAGATTGCTGGGATCTTGTTAACAAATGCACAAAGTCAAGCGGAAAAGATAGACC 6600
 V  M  D  I  A  D  P  A  I  L  E  E  H  G  I  A  I  Q  Q  Q  L  K  D  C  W  D  L  V  N  K  C  T  K  S  S  G  K  D  R  P

ATACATGATTGAAGTTGCAAGGGAATTACACCGAATTCACAAGTGCTTCCGTGCATGCCCTTAGtcttggccacaattagtacaataagatggccaaaaaaaaaaaaatcaggtgcacaa 6720
 Y  M  I  E  V  A  R  E  L  H  R  I  H  K  C  F  R  A  C  P  *

gaattaagcttagctttatttgtgcggtcatattatgtcggatagtaaaaaaagatcagcacataatgaaattgtcaatttttttttttttaatttgcgacatcatttccatttttaactt 6840

ggctcatatatgaaaaatacaattttctttttatttttttcattttaacaaatcaatagagatttaatttattattttatattaatattactctgtaactatttataggattttcaaagaa 6960

aaatttaataaaacaacccctaaagtgtcaaatcaataattactaagtacaaatttgtacggtggcaacgaaagcgacggccgaaatattgagaagagacgctagagcaaatgcaaactt 7080

ccttatagagcggatcttttgtcaacttgagtggaattaacgccgtgtttggtatgagaaaaatattttcatgaaaaataaattgattatatttgattggtgagtaaaaaatatttttta 7200

aaaaatagcgtttgtttggtaaatagaaaatatatttttttagaaaaatatcttttagtgttgattaatgaacaaacaaaatattatttgaaaaatactactaaattgttaactaacatat 7320

cattgcctctaataactcaacaatttgtagaaactaatttaagtatagcaatcaatacgaatattgtgtaccaaaatattacaagtcaataaaattaaagcaactactaattatataatc 7440

caagtttcctaacaatattccctaaaattaagggtgttaagtgggccggccgggtcaactcggaatcgtcctgttaagtttagctgggttgtgggctaggctggatcgggccgggttgta 7560

gttaagttggccgggtcgggtccgggttcaacagtaataaaaataactctgaatcggcccacttagcccaacccagtct                                          7639
                                                                                   <------- prBs6A_R -------|
```
