# Supplementary material for: Map-based cloning of the susceptibility locus conditioning bs6 resistance in pepper (Capsicum annuum L.) encoding mutant alleles of pseudokinase proteins
Source: Theor Appl Genet. 2026 Oct 20;139(11):308. doi: 10.1007/s00122-026-05407-2 (PMC13683501; doi:10.1007/s00122-026-05407-2)
Supplement: Supplementary file 7 [file 122_2026_5407_MOESM7_ESM.pdf]

```
CaFo     ::::::::::::::::::::::::::::::::::::::::::::::::::C:::::::::::::::::::::::::::::::::::::::::::::::::::::::::::::::::::::
Ca61R    ::::::::::::::::::::::::::::::::::::::::::::::::::T:::::::::::::::::::::::::::::::::::::::::::::::::::::::::::::::::::::
P164-F3  TAAAATTGGTAGTGCAACACATGCCCCAACATTGTCTTACATAAAGTTTTCTTTCTTTCTTCAAGTATAACCTAATTTCTATTTATTCTTTAACTTCCCATTCTGATAGCATTGTTGCCT
               pr1641F-->

CaFo     ::::::::::::::::::::::::::::::::::::::::::::::::::::::::::::::::::::::::::::::::::::::::::::::::::::::::::::::::::::::::
Ca61R    ::::::::::::::::::::::::::::::::::::::::::::::::::::::::::::::::::::::::::::::::::::::::::::::::::::::::::::::::::::::::
P164-F3  TCTCAAATTATTCTTGTCTTTTACAGGCTCTTCAATGAAGACGCCTCAGGATATTGGTAGAGATAGAGATGCCATATATCTTCTATGATCTGCATATCTTCTATGATCTGTATGTCTGAA

CaFo     ::::::::::::::::::::::::::::::::::::::::::::::::::::::::::::::::::::::::::::::::::::::::::::::::::::::::::::::::::::::::
Ca61R    ::::::::::::::::::::::::::::::::::::::::::::::::::::::::::::::::::::::::::::::::::::::::::::::::::::::::::::::::::::::::
P164-F3  GAACAACACCATACACTCATATAACCATTCAGCTCATACTGCTTCTATTTCTTCCTATGCACACATAGACCAAATTGCAAGTTTCATTTTAATAAAACTTGTAGCTCATCTATCCATATG

CaFo     ::::::::::::::::::::::::::::::::::::::::::::::::::::::::::::::::::::::::::::::::::::::::::::::::::::::::::::::::::::::::
Ca61R    ::::::::::::::::::::::::::::::::::::::::::::::::::::::::::::::::::::::::::::::::::::::::::::::::::::::::::::::::::::::::
P164-F3  TGCTTTCCCTTTCATTTCAATACTATTTGTTCTCCATTTGTTCAAAATAGATCTTTCCCTGCTACTACTGAATGTATTTCTGAAACTGTCTTTAATAAAGTTGTTGTTCGCAATAAATCT

CaFo     ::::::::::::::::::::::::::::::::::::::::::::::::::::::::::::G:::::::::::::::::::::::::::::::::::::::::::::::::::::::::::
Ca61R    ::::::::::::::::::::::::::::::::::::::::::::::::::::::::::::A:::::::::::::::::::::::::::::::::::::::::::::::::::::::::::
P164-F3  CATTCTATATCTTTAAATATATATTTTTCTTATGAAAATGATGTTAATGTCCTGTGGCACGATAGACTTGGGCATGCGCCCTTTAGAAAAATGAGAGAAATTCACTACGTACCAGATAAA

-
CaFo     ::::::::::::::::::::::::::::::::::::::::::::::::::::::::::::::::::::::::::::::::::::::::::::::::::::::::::::::::::::::::
Ca61R    ::::::::::::::::::::::::::::::::::::::::::::::::::::::::::::::::::::::::::::::::::::::::::::::::::::::::::::::::::::::::
P164-F3  TCTTTCCTTAGGCAATTTTTTCTTTGCTTTATTTGTCCCATGACTAGAAAAGAGAGACTTTCATTCCCTCAAAGAACCACAAGCTCTATTGAGATTTTCAACTTCTCAATGTAGATTTAT

CaFo     ::::::::::::::::::::::::::::::::::::::::::::::::::::::::::::::::::::::::::::::::::::::::::::::::::::::::::::::::::::::::
Ca61R    ::::::::::::::::::::::::::::::::::::::::::::::::::::::::::::::::::::::::::::::::::::::::::::::::::::::::::::::::::::::::
P164-F3  GGGTCCTTATCATACAATGACCCATGATAAGCTCATGTACTTCTTCACCATGGTATATTACTATAGTAGAGCTACCTGGACATAACACTTAACCTGCAAAGATAATGCCGTGAAAGTCAT

CaFo     ::::::::::::::::::::::::::::::::::::::::::::::::::::::::::::::::::::::::::::::::::::::::::::::::::::::::::::::::::::::::
Ca61R    ::::::::::::::::::::::::::::::::::::::::::::::::::::::::::::::::::::::::::::::::::::::::::::::::::::::::::::::::::::::::
P164-F3  AAAGAATTTCACTACCATGGTTGAAAACCAATTTAAAACCACAATAAAATCCATTAGATCTGACAGTGGACTATAATTTGTTAATACAGAACCAATATTATTTTTTCAAACATGTCCATA

CaFo     ::::::::::::::::::::::::::::::::::::::::::::::::::::::::::::::::::::::::::::::::::::::::::::::::::::::::::::::::::::::::
Ca61R    ::::::::::::::::::::::::::::::::::::::::::::::::::::::::::::::::::::::::::::::::::::::::::::::::::::::::::::::::::::::::
P164-F3  TACACCCTAATAAAATGGTGTGGTAAAAAAAAATACAAACACCTATTAGAGACTGCTAGATCCCTTCTTTTACAGTTCAAACTTACCATAAAGTATTGGAGAGAATGTATCTTATATGCC

CaFo     ::::::::::::::::::::::::::::::::::::::::::::::::::::::::::::::::::::::::::::::::::::::::::::::::::::::::::::::::::::::::
Ca61R    ::::::::::::::::::::::::::::::::::::::::::::::::::::::::::::::::::::::::::::::::::::::::::::::::::::::::::::::::::::::::
P164-F3  ACCTATTTCATCAATAGATTACCCCTAGTTACATAAAGAACAAGTACCCATATAAAATTCTTTACAAAAGAAAACCAACATACTAACACATGAAAAATTTTAGTTGTTTATGTTATCCTA

CaFo     ::::::::::::::::::::::::::::::::::::::::::::::::::::::::::::::::::::::::::::::::::::::::::::::::::::::::::::::::::::::::
Ca61R    ::::::::::::::::::::::::::::::::::::::::::::::::::::::::::::::::::::::::::::::::::::::::::::::::::::::::::::::::::::::::
P164-F3  GAGTCCCTAAACCACAACGAGATAAATAGGAACCTAAAACTACTCCCCATGTCTTTATTGGATACTCTTATTGTGTTAAAAGCTAGAAAATGAAGGTCTAGCCACCAGGAAAATTTGCAT

CaFo     ::::::::::::::::::::::::::::::::::::::::::::::::::::::::::::::::::::::::::::::::::::::::::::::::::::::::::::::::::::::::
Ca61R    ::::::::::::::::::::::::::::::::::::::::::::::::::::::::::::::::::::::::::::::::::::::::::::::::::::::::::::::::::::::::
P164-F3  TTCCAAAGATGTAGTTTTTAAACAAACTGTCTATCCTTTTACAATTGATCCTAATCTTACTTCATTCCCTTCTATTCTTACTTTTGTTCTATTCATTGATTATGATCTTGTCTACAAAAA

CaFo     ::::::::::::::A:::::::::::::::::::::::::::::::::::::::::::::::::::::::::::::::::::::::::::::::::::::::::::::::::::::::::
Ca61R    ::::::::::::::T:::::::::::::::::::::::::::::::::::::::::::::::::::::::::::::::::::::::::::::::::::::::::::::::::::::::::
P164-F3  TATTTTTGATGACCTAGGTATATGTGATGATACAAGTAGTATAACCCATCCTATAGAACTTATTACAAACCCATTTCATCCACTCCTCACATTTCGTCAAATGATGATCCATGCACTGCA

CaFo     ::::::::::::::::::::::::::::::::::::::::::::::::::::::::::::::::::::::::::::::::::::::::::::::::::::::::::::::::::::::::
Ca61R    ::::::::::::::::::::::::::::::::::::::::::::::::::::::::::::::::::::::::::::::::::::::::::::::::::::::::::::::::::::::::
P164-F3  TCCAATCCAACTTCCACACCAGATAGCACTGGACAACTTACTATAGAACCAGAAAAGTCTCAAAGAGGTTTTAAGCCTCCTAGACATTTTCATGATTACATACACAAATTACCCAATCTC

CaFo     ::::::::::::::::::::::::::::::::::::::::::::::::::::::::::::::::::::::::::::::::::::::::::::::::::::::::::::::::::::::::
Ca61R    ::::::::::::::::::::::::::::::::::::::::::::::::::::::::::::::::::::::::::::::::::::::::::::::::::::::::::::::::::::::::
P164-F3  AAACCTACGTTAAACACATCCATATCTCTCACTGCATTTTTCTCCCATTGTAATTACATTTCTCCTGATGTTTTGAGTCCTGAAAGCTTGAGTCTTGTGAATAATATTTGCACTTATAGT

CaFo     ::::::::::::::::::::::::::::::::::::::::::::::::::::::::::::::::::::::::::::::::::::::::::::::::::::::::::::::::::::::::
Ca61R    ::::::::::::::::::::::::::::::::::::::::::::::::::::::::::::::::::::::::::::::::::::::::::::::::::::::::::::::::::::::::
P164-F3  GAACCTACTTTATATGGAGAGACATCTATGAATCTTGCCTGGCAAACTTCCATGAGTCAAGAATTTGAGGCACTGCACCTGAATCACATTTGGAATTTAGTATTGCTACCTCATGGTAAA

CaFo     ::::::::::::::::::::::::::::::::::::::::::::::::::::::::::::::::::::::::::::::::::::::::::::::::::::::::::::A:::::::::::
Ca61R    ::::::::::::::::::::::::::::::::::::::::::::::::::::::::::::::::::::::::::::::::::::::::::::::::::::::::::::T:::::::::::
P164-F3  CAAGATATTGGGTATGTCCTGCCCTATTTTTATTAAGGTTAAATAAAACATGACCTATTTATGAACTGTTAAAGAATGAAATTTTGGATAGAATCACCACCCAATTTTTAAGAAAAATAA

CaFo     ::::::::::::::::::::::::::::::------------------------------------------------------::::::::::::::::::::::::::::::::::::
Ca61R    ::::::::::::::::::::::::::::::TACCTATTTAATTACTAACATGTATGATACCTATTTAATTACTAACATGTCTGA::::::::::::::::::::::::::::::::::::
P164-F3  GAATACCTATTTAATTACTAACATGTATGATACCTATTTAATTACTAACATGTATGATACCTATTTAATTACTAACATGTCTGACTTATATTTTAGTTAGTGAAAATCGATTTAGATTTT

CaFo     :::::::::::::::::::::::::::::::::::::::::T::::::::::::::::::::::::::::::::::::::::::::::::::::::::::G::::::::::::::::::
Ca61R    :::::::::::::::::::::::::::::::::::::::::C::::::::::::::::::::::::::::::::::::::::::::::::::::::::::A::::::::::::::::::
P164-F3  GAGTAAAGGTTCAAATTATTTCAAAGAAAAGAGGTTAGATACCCTTCAAAATCCACAAATATGATACTCGACTAGACTGAATTTATCAAAGAAGAAGGAGATACAAAATTATTCTTTTTA

CaFo     ::::::::::::::::::::::::::::::::::::::::::::::G:::::::::::::::::::::::::::::::::::::::::::::::::::::::::::::::::::::::::
Ca61R    ::::::::::::::::::::::::::::::::::::::::::::::C:::::::::::::::::::::::::::::::::::::::::::::::::::::::::::::::::::::::::
P164-F3  AAAAATATAAACATTTAAAATAACATAAAAGATGTATGCATATTAACTATATAAATAAAATCTATTGTTATAAAATATGTATATAAAAATATGTAAAAACATACATAAAAATGTACAAGA

CaFo     :::::::::::::::-:::::::::::::::::::::::::::::::::::::::::::::::::::::::::::::::::::G::::::::::::::::::::::::::::::::::::::
Ca61R    :::::::::::::::A:::::::::::::::::::::::::::::::::::::::::::::::::::::::::::::::::::T::::::::::::::::::::::::::::::::::::::
P164-F3  TACGTATGAAAGAAAAGTACTTTGTAACATACGAAAAGAAAAGTATTTTTATGTACTATAAAAAGAAAAGTATTTTCAAATAATACTTTTTACTTGACCAAATTACGGGGATGAAAAATAAA
                                                                                                        <------ pr1642R --------
```
