# Supplementary material for: Map-based cloning of the susceptibility locus conditioning bs6 resistance in pepper (Capsicum annuum L.) encoding mutant alleles of pseudokinase proteins
Source: Theor Appl Genet. 2026 Oct 20;139(11):308. doi: 10.1007/s00122-026-05407-2 (PMC13683501; doi:10.1007/s00122-026-05407-2)
Supplement: Supplementary file 8 [file 122_2026_5407_MOESM8_ESM.pdf]

```
              10        20        30        40        50        60        70        80        90       100       110       120       130       140       150       160       170       180       190       200
               |         |         |         |         |         |         |         |         |         |         |         |         |         |         |         |         |         |         |         |
CaFo     ::::::::::::::::::::::::::::::::::::::::::::::::::::::::::::::::::::::::::::::::::::C:::::::::::::::::::::::::::::::::::::::::::::::::::::::::::::::::::::::::::::::::::::::::::::::::::::::::::::::::::
Ca61R    ::::::::::::::::::::::::::::::::::::::::::::::::::::::::::::::::::::::::::::::::::::T:::::::::::::::::::::::::::::::::::::::::::::::::::::::::::::::::::::::::::::::::::::::::::::::::::::::::::::::::::
P1023-F3 TATTGGGTATTGAAGTTTGTGCTGCCTCGCATGGTCTATTATTATGTCAATCCAAATATATCAGAGATTTATTAACTAGAGCTTTTATGGAAGGTGCAAAGTCTATATCGACACCCATGGTAATTGGTCTTCAACTTTCCCAGCATGGTAGCCCAATGTTGATCAACCGGAACTTATTCGGAGTATTGTTGGAGCATTA
200
        |------- pr1637F ------->

CaFo     :::::::::::::::::::::::::::::::::::::::::::::::::::::::::::::::::::::::::A::::::::::::::::::::::::::::::::::::::::::::::::::::::::::::::::::::::::::::::::::::::::::::::::::::::::::::::::::::::::::::::::::
Ca61R    :::::::::::::::::::::::::::::::::::::::::::::::::::::::::::::::::::::::::G::::::::::::::::::::::::::::::::::::::::::::::::::::::::::::::::::::::::::::::::::::::::::::::::::::::::::::::::::::::::::::::::::
P1023-F3 CAATATATCACAATTACCCGACCTGATGTCTCCTTTGCTGTTAACAAGTTGTGTCAGTTTATGCATGATCCTCGCGAAATGCATTATACAGCTATGAAGAGGTTGCTTAGATATCTAAAAGGTACAATTGATCTTGGTTTACTGTTGCGTTCATCTGCTTCCTTCACACTTACTGGTTTCTCGGATGCTGACTGGGCTGG
400

CaFo     :::::::::::::::::::::T:::::::::::::G::::::::::::::::::::::::::::::::::::::::::::::::::::::::::::::::::::A:::::::::::::::::::::::::::::::::::::G:::::::::::::::::::::::::::::::::::::::::
Ca61R    :::::::::::::::::::::C:::::::::::::A::::::::::::::::::::::::::::::::::::::::::::::::::::::::::::::::::::G:::::::::::::::::::::::::::::::::::::A:::::::::::::::::::::::::::::::::::::::::
P1023-F3 CTTCCCAGATGAACGACGTTCCACTCATGGCTTCTACATATTTTTGGGTGCCAACCTGGTGTCATGGAGCTCCAAAAAAACAACGTATAGTTGCTCGCTCTAGCACAGAATCAGAGTATCGAGGTCTTGCTACAACCACAGCTGAAATAACTCGGCTGAAAAATCTACTTAAAGAACTCCAAATTGTGCTTCCAGCTGCT
600

CaFo     :::::::::::::::::T::::::::::::::::::::::::::::::::::::::::::::::::::::::::::::::::::::::::::::::::::::::::::::::::::::::::::::::::::::::::::G:::::::::::::::::::::::::::::::::::::::A:::::
Ca61R    :::::::::::::::::A::::::::::::::::::::::::::::::::::::::::::::::::::::::::::::::::::::::::::::::::::::::::::::::::::::::::::::::::::::::::::A:::::::::::::::::::::::::::::::::::::::G:::::
P1023-F3 CCAAAAGTATGGTGTGAAAATTTATCAGCCACATACCTCGCTGTCAACCCTGTGTTCCATGCTCGCACTAAACATATTGAACTTGATTTTCACTTTGTGGGGGAGAAGGTAGTTTCAAGAGACATGAAAGTCTGCTACATAAGTTCTATGGATCAGGTGGCAGATATCATGACTAAAAGTCTTGCTACACGTCGGTTTC
800

CaFo     :::::::::::::::::::::::::::::::::::::::::::::::::::::::::::::::::::::::::::::::::::::::::::::::::::::::::::A::::::::::::::::::::::::::::::::::::::::::::::::::::::::::::::::::::::::::::::::::::::::::::
Ca61R    :::::::::::::::::::::::::::::::::::::::::::::::::::::::::::::::::::::::::::::::::::::::::::::::::::::::::::T::::::::::::::::::::::::::::::::::::::::::::::::::::::::::::::::::::::::::::::::::::::::::::
P1023-F3 TATTTCTCAGGACAAGTTTGCTATGGTTGTACCATTGCTGCACTTACGTGGGGCTGTAAGGATAAAGGAATTGCCTAATAGATAATGATACTGTTTTGATTTTAAAATTTCAAATGTATAGTTTGCAATTATATTCCTTTATCTAGTGGATATATGATTAGCTTAAACTCACATACTAGAGTCCTACTTGGACTCTTCAT
1000
                                                                                                                                                                                                     <----- pr1637R --
CaFo     ::::::::::::::::::::::::::::::::::::::::::::::::::::::::::::::::::::::::::::::::::::::::::::::::::::::::::::::::::::::::::::::::::::::::::::::::::::::::::::::::::::::::::::::::::::::::::::::::::::::::
Ca61R    ::::::::::::::::::::::::::::::::::::::::::::::::::::::::::::::::::::::::::::::::::::::::::::::::::::::::::::::::::::::::::::::::::::::::::::::::::::::::::::::::::::::::::::::::::::::::::::::::::::::::
P1023-F3 AGCAGCAACTTTATGTATATATATAGAGGTCCAATCCTCTAAACGTAATATAATATATTCCAGGATTAAATCCTTTCAAATTGTAACCAAAGAAATAATTGTTTTTTATAATTCTAAAATCAACAATAAAAGCTAAGACGTAATTAGATGGGTTGTAGACAATACTCCTTTCATATCACCCAAAATGCTAAAAAGACACT
1200
         ----|

CaFo     :::::::::::::::::::::::::::::::::::::::::::::::::::::::::::::::::::::::::::::::::T::::::::::::::::::::::::::::::::::::::::::::::::::::::::::::::::::::::::::::::::::::::::::::::::::::::::::::::::::::::
Ca61R    :::::::::::::::::::::::::::::::::::::::::::::::::::::::::::::::::::::::::::::::::-::::::::::::::::::::::::::::::::::::::::::::::::::::::::::::::::::::::::::::::::::::::::::::::::::::::::::::::::::::::
P1023-F3 AGTATTAAGTGGCAAGATGTTTGCCCCAACCCCGATCTTGACTCTTGACCTTGATTCGAGACTCTATTTTGACCTTGACTT-AGACCCGAGACATGATTTTGGATCTCGACCAGGGATAAAATTCCCAATCCCATCCCATAATCCATTTCTTGATCCCCGACTCTGACTACTTGGAATTCGTTTCTCGATCCCCTACCTT
1400

CaFo     ::::::::::::::::::::::::::::::::::::::::::::::::::::::::::::::::::::::::::::::::::::::::::::::::::::::::::::::::::::::::::::::::::::::::::::::::::::::::::::::::::::::::::::::::::::::::::::::::::::::::
Ca61R    ::::::::::::::::::::::::::::::::::::::::::::::::::::::::::::::::::::::::::::::::::::::::::::::::::::::::::::::::::::::::::::::::::::::::::::::::::::::::::::::::::::::::::::::::::::::::::::::::::::::::
P1023-F3 GACTCGGGATCGTCACCTAACTTGGAACCCACTCCTTATCTGATTCTAGACCCAACCCTGTGTTGGGAAACAACAAAATAAAAATGTTAACAAAATATTATAATAATAATATTAACAATACAATAACATAACAGTCACCTAACTACTTCTTAACAAGTTATGATTCTAGACCCAACCCTGTGTTGGGAAACAACAAAATA
1600

CaFo     ::::::::::::::::::::::::::::::::::::::::::::::::::::::::::::::::::::::::::::::::::::::::::::::::::::::::::::::::::::::::::::::::::::::::::::::::::::::::::::::::::::::::::::::::::::::::::::::::::::::::
Ca61R    ::::::::::::::::::::::::::::::::::::::::::::::::::::::::::::::::::::::::::::::::::::::::::::::::::::::::::::::::::::::::::::::::::::::::::::::::::::::::::::::::::::::::::::::::::::::::::::::::::::::::
P1023-F3 AAAATGTTAACAAAATATTATAATAATAATATTAACAATACAATAACATAACAGTCACCTAACTACTTCTTAACAAGTTAAATAAGTCTTTCCTAATTAACCAGAGTATACATGAATCAACAAACTATTAATATAAATATCAACAAATTAAACTATAGGTTTACTAAATACTAACTAAAATCACTAAGGAATATAATAT
1800

CaFo     ::::::::::::::::::::::::::::::G:::::::::::::::::::::::::::::::::::::::::::::::::::::::::::::::::::::::::::::::::::::::::::::::::::::::::::C:::::::::::::::::::::::::::::
Ca61R    ::::::::::::::::::::::::::::::A:::::::::::::::::::::::::::::::::::::::::::::::::::::::::::::::::::::::::::::::::::::::::::::::::::::::::::T:::::::::::::::::::::::::::::
P1023-F3 AGAAGGGAAGAGGAAAATGTCAAAAGAAAAGAGATTGAAAGCAGGAATGTTCCTTCTTTTCTGTAAGGATTGAGAGAGTATTGTAACTAAGACTTAAATTCTCAAGTACATCTCTTGATGACTTCTAGAGTAAGGTTTCTCAAGTACATCTCTTGATGACTTCCAGAGCTTTCTCAAGTATATCTCTTGATAACTTTCA
2000

CaFo     :::::::::::::::::::::::::::::::::::::::::::::T::::::::::::C:::::::::::::::::::::::::::::::::::::::::::::::::::::::::::::::::::::::::::::::::::::::::::::::::::::::::::::::::::::::::::::::::::::::::::::
Ca61R    :::::::::::::::::::::::::::::::::::::::::::::C::::::::::::T:::::::::::::::::::::::::::::::::::::::::::::::::::::::::::::::::::::::::::::::::::::::::::::::::::::::::::::::::::::::::::::::::::::::::::::
P1023-F3 AAGTAAGCCTTCTCAAGTACATTTGTTGATGACTTTCAAAGTAAGCCTTTTAAGTAGCTTCTAAGATACATTTAAAGACATGACTTTTTCAATACACTTCAAATATAAATTATTTTGAAATAACTAAATAATTTTCCTACACCCTAACCCCACTTTGACCCTGATCCAAGATTGGACTCCCGGCCTCGACCAGGAACCCA
2200

CaFo     ::::T::::::::::::::::::::::::::::::::::::::::::::::::::::::::::::::::::::::::::::::::::::::::::::::::::::::::::::::::::::::::::::::::::::::::::::::::::::::::::::::::A::::::::::::::::::::::::::::::
Ca61R    ::::C::::::::::::::::::::::::::::::::::::::::::::::::::::::::::::::::::::::::::::::::::::::::::::::::::::::::::::::::::::::::::::::::::::::::::::::::::::::::::::::::T::::::::::::::::::::::::::::::
P1023-F3 ATTCCAATCAGTAACCAGCCTCTAAAACCGACCCAAGAGTTGGATCTCGGGTTGGGATCGGACTCAGATAGAAAAGACATTTTTCTTGAATTTGAGAAGAATAAGTTCACTCAGAAAAATATTTTTCAAAACATTAATTTAACTAACCAAACATGAAAACATTGGAGTTCCAACCTACCAAACACCCATTCTATACTGTT
2400

CaFo     ::::::::::::::::::::::::::::::::::::G:::::::::::::::::::::::::::::::---::::::::::::::::::::::::G::::::::::::::::::::::::::::::::::::::::::::::::::::::::::T:::::::::::::::::::::::::::::::::::::::::::::
Ca61R    ::::::::::::::::::::::::::::::::::::A:::::::::::::::::::::::::::::::TAC::::::::::::::::::::::::C::::::::::::::::::::::::::::::::::::::::::::::::::::::::::A:::::::::::::::::::::::::::::::::::::::::::::
P1023-F3 TTTCATGAAGTTCAAATTCTAGTCAAGAGAGACACTACAGCCAGTGACAATGAAACTCCCCAATAACTACGTGAGAGACTCTAGTCAAGACCAAACTAACGTGGAACAATGCACACCCTCTAGTACACGGACCATCAGGAAACTTCCCAACTAAAAGATTCATTCCACGCGCGAGAGATCAAGAGTTGGATCAGAGAAAC
2600

CaFo     ::::::::::::::::::::::::::::::::::::::::::::::::::::::::::::::::::::::::::::::::::::::::::::::::::::::::::::::::::::::::::::::::::::::::::::::::::::::::::::::::::::::::::::::::::::::::::::::::::::::::
Ca61R    ::::::::::::::::::::::::::::::::::::::::::::::::::::::::::::::::::::::::::::::::::::::::::::::::::::::::::::::::::::::::::::::::::::::::::::::::::::::::::::::::::::::::::::::::::::::::::::::::::::::::
P1023-F3 CAGACTTATGAATTTATTTGTTGCTATTGCAATTGGTCTTGGACAAGAACAAAATGCATCATTTGATGAGTGCACTACTCACAACCAAAAAACTTTTTTCCTCTGCCTCCGTAAAAAAGCGGAAGAAAGAGCAGGATTGCTATTTAAATAATGGAAGTGCAGTGCTAGAGGAGATTCTTGCTTATGCAACGGAAATTGC
```

2800 ORF-2 **start codon 2654 nt**

|--> *bs6[61]npk*

```
CaFo     :::::::::::::::::::::::::::::::::::::::::::::::::::::::::::::::::::::::::::::::::::::::::::::::::::::::::::C::::::::::::::::::::::::::::::::::::::::::::::::::::::::-:::::::::::::::::::::::::::::::::::
Ca61R    :::::::::::::::::::::::::::::::::::::::::::::::::::::::::::::::::::::::::::::::::::::::::::::::::::::::::::A::::::::::::::::::::::::::::::::::::::::::::::::::::::::T:::::::::::::::::::::::::::::::::::
P1023-F3 CGAATTCCCATCCGTTACTTCACTGCCACTGAGATAGAAAGGGCGATCAAGCACTCTGAAAAGAAAATAGAACTCGACGAAGGCTATATGGTTACAGGCTCCCTAGAAAACCACCTCTTTTTAATTAGGTTCTCTCTCTATTCCTATGGCGTGCACCGAGATATTAGCAATTACAGCTCAGATGAGTCATCTCAAGAATG
3000

CaFo     ::::::::::::::::::::::::::::::::::::::::::::::::::::::::::::::::::::::::::::::::::::::::::::::::::::::::::::::::::::::::::::T:::::::::::::::::::::::::::::::::::::::::::::::::::::::::::::::::::::::::::
Ca61R    ::::::::::::::::::::::::::::::::::::::::::::::::::::::::::::::::::::::::::::::::::::::::::::::::::::::::::::::::::::::::::::A:::::::::::::::::::::::::::::::::::::::::::::::::::::::::::::::::::::::::::
```

P1023-F3 TGCTGAGACTTATTGGTTGCTGTCTAGAATT**TGA**AGAACCGGTTATGGTGTATGAATATATTGAAGGGATATCTCTCGGCGATCTACTTTTCGAAGAGGGTAATCATGATGATCAACTAGAAAATCACTATTATCTTGGGGAAGTAGATTACGAATTGCCAATGAGGTTGCTTCTGCACTCGTCTTTCTCCATACCGAA

3200

bs6[61]npk-->|

```
CaFo     ::::::::::::::::::::::::::::::::::::::::::::::::::::::::::::::::::::::::::::::::::::::::::::::::::::::::::::::::::::::::::::::::::::::::::::::::::::::::::::::::::::::::::::::::::::::::::::::::::::::::
Ca61R    ::::::::::::::::::::::::::::::::::::::::::::::::::::::::::::::::::::::::::::::::::::::::::::::::::::::::::::::::::::::::::::::::::::::::::::::::::::::::::::::::::::::::::::::::::::::::::::::::::::::::
P1023-F3 TTTACTACACCTATCATCCACAGAGATATAAAGCCTCACAAAGTGATAATAGATCAGAATAGCAGCATTGCCAAAATAGTGGACTTCTCATTTTCCATATCATTGCCTTCAGGGGAATTGGAGGTACAAGATCGTGTGTGCGGAACAATCGAGTATTTAGATCCAGAATATGGCTACTGTGGTATTATTACTCAAAAAAC
3400

CaFo     ::::::::::::::::::::::::::::::::::::::::::::::::::::::::::::::::::::::::::::::::::::::::::::::::::::::::::::::::::::::::::::::::::::::::::::::::::::::::::::::::::::::::::::::::::::::::::C:::::::::::::
Ca61R    ::::::::::::::::::::::::::::::::::::::::::::::::::::::::::::::::::::::::::::::::::::::::::::::::::::::::::::::::::::::::::::::::::::::::::::::::::::::::::::::::::::::::::::::::::::::::::T:::::::::::::
P1023-F3 TGACGTGTACAGCTTTGGAGTTCTTCTCTTTGAGCTATTAACTGGAAAGGAATTGCGTGATATTATGGAATCCAACGTTGAAGAGGGTAATGTTATGGATAGAGTGGATTCTGCAATTTCAGAAGAGACAATAAATTTTGAAAATTTTCTACCTAACCTCATCGATCGCTATATTAAAGAGGGTAATATAATGGACATAG
3600

CaFo     ::::::::::::::::::::::::::::::::::::::::::::::::::::::::::::::::::::::::::::::::::::::::::::::::::::::::::::::::::::::::::::::::::::::::::::::::::::::::::::::::::::::::::::::::::::::::::::::::::::::::
Ca61R    ::::::::::::::::::::::::::::::::::::::::::::::::::::::::::::::::::::::::::::::::::::::::::::::::::::::::::::::::::::::::::::::::::::::::::::::::::::::::::::::::::::::::::::::::::::::::::::::::::::::::
P1023-F3 CAGATAATGCCATTTAGAAGAGCATGGAATTGAGATCCAACAGCAATTGCAAGATTACTTGAATCTTGTGAAGAAATGCACAACTTACAACAGAAAAAATAGACCATACATGATTCAAGTTGCAAAGGAATTATGCCGAATTCACAAGTGCTTCCATGCCTTCACTCTTGGCCAGATTTAGCTATATAAGATGGGGAA
3800 ORF-2 stop codon 3780 nt

CaFo     ::::::::::::::::::::::::::::::::::::::::::::::::::G:::::::::::::::::::::::::::::::::::::::::::::::::::::::::::::::::::::::::::::::::::::::::----------------
Ca61R    ::::::::::::::::::::::::::::::::::::::::::::::::::A:::::::::::::::::::::::::::::::::::::::::::::::::::::::::::::AACAGGGAAAAGGTCTTACTAAATTCAATTGCTTTTTAAA
P1023-F3 AAAATTAAACAGCACATCATTTGCATGAAATAATCATGGCTTTATCTGTAATGGTTTCAAGTTTCAAAGGAAATATAGTTTGTTTGCAGAGTCATATTATGTAGGGATTTGGACGATAGAAGAGATCTGTAGATAATGAAATTGTCAATTTGGCATACTTAACAGGGAAAAGGTCTTACTAAATTCAATTGCTTTTTAAA
4000

CaFo     --------------------------------------------------------------------------------------------------------------------------------------------------------------------------------------------------------
Ca61R    TACAACATCTGATATATGAATATTCAGGATAAATGTTATTCTCTTCGTTTCATAATAAATAAATTATTAAATTTTGACACATATATTAAAAAAAAATCACTAAAAATATGAATTTAACACAATTTTTTATTTTTATCTCAAAAAAAAGTTGATGTAATAATACGTTTTCAAAAGTTAATTGATTGTCAAGTTATAAGAAT
P1023-F3 TACAACATCTGATATATGAATATTCAGGATAAATGTTATTCTCTTCGTTTCATAATAAATAAATTATTAAATTTTGACACATATATTAAAAAAAAATCACTAAAAATATGAATTTAACACAATTTTTTATTTTTATCTCAAAAAAAAGTTGATGTAATAATACGTTTTCAAAAGTTAATTGATTGTCAAGTTATAAGAAT
4200

CaFo     -----------------------------------------------------------GCTAGTTACATCGATCTTTTCAGTGCTTAATGAAGAAGCCAGGTATCGAGATAGGTCAATTTGAAATTTATGGGTTC::::::::::::::::::::::::::::::::::::::::::::::::::::::::::::-:::
Ca61R    AATTTTTTAAAAAAAAATTAATGATTCATTTATTTTAAAATATCAATAAATATCCTAACAATTTATTTATTTTGAAAAGGAGGAAGTAATTGTGTCACCGTTGAAAATATTGGAAGATGAGATTGTAACTTATCAA::::::::::::::::::::::::::::::::::::::::::::::::::::::::::::G:::
P1023-F3 AATTTTTTAAAAAAAAATTAATGATTCATTTATTTTAAAATATCAATAAATATCCTAACAATTTATTTATTTTGAAAAGGAGGAAGTAATTGTGTCACCGTTGAAAATATTGGAAGATGAGATTGTAACTTATCAAGTCATGTTAAATTTCCTCTCTGTTATGATAACAGGGATATTCTCAGATTTAATACAGTAGTAG
4400

CaFo     :::::::::::::::::::::::::::::::::::::::::::::::::::::::::::::::::::::::::::::::::::::::::::::::::::G:::::::::::::::::::::::::::::::::::::::::::::::::::::::::::::::::::::::C::::::::::::::::::::::::::::
Ca61R    :::::::::::::::::::::::::::::::::::::::::::::::::::::::::::::::::::::::::::::::::::::::::::::::::::C:::::::::::::::::::::::::::::::::::::::::::::::::::::::::::::::::::::::A::::::::::::::::::::::::::::
P1023-F3 TTAATTTGCCTGCAAGAAGAAACCTTTTTATTTTTACATTTATCAAAACTTGATGTTGTTACCTTTATTTTTTCCTTCAGAAGATATTTTGTAAGAGGGCTGAAAAAGAAATTAAAGGAGGGCAAATATGGCAAAGAAGCAAGATGATTGGAAAGTGGGACCTAAATCAGTACATCTTTCAAATCAAAGGCCATTTATTG
4600

CaFo     ::::::::::::::::::::::::::::::::::::::::::::::::::::::::::::::::::::::::::::::::::::::::::::::::::::::C:::::::::::::::::::::::::::::::------------------------------------------------------------------
Ca61R    ::::::::::::::::::::::::::::::::::::::::::::::::::::::::::::::::::::::::::::::::::::::::::::::::::::::T:::::::::::::::::::::::::::::::TTATGTTTTATTTTAGGCATAAAATAATATATATTTTTCAATTGGTGTATTAATAGTTTTTTAA
P1023-F3 TCATTTTGTGTGGAAATTGTCAAGTTCAAAAGAGCAGCAGGATCCAGGAAGCTCACAACTTCCTTATCCATTCATTCCCACTAGTGTTGCCTTATTGGATCTAATCCAACGAGTAATGGCCCATTTATTGTCATTATGTTTTATTTTAGGCATAAAATAATATATATTTTTCAATTGGTGTATTAATAGTTTTTTAA
4800

CaFo     -------::::A:::::::::::A:::::::::::::::::::::::::::::T::::::::::::T:::::::::G::::::::::::::G:::::::C::::::::::::::::::::::::::::::::::::::::::::::::G:::::::::::::::::::::::::::::::::::::::::::::::::::
Ca61R    TTTAAAA::::G:::::::::::G:::::::::::::::::::::::::::::G::::::::::::A:::::::::A::::::::::::::C:::::::T::::::::::::::::::::::::::::::::::::::::::::::::A:::::::::::::::::::::::::::::::::::::::::::::::::::
P1023-F3 TTTAAAAAATAGTGTATTTTTAGGCACAATCCTAATATCATTAATTTTTTTTAAGAAAAGCTTCTCAAATTCATATCAGGTAAATATAAGTACCATTAAATAAAGAAAATCAATTGAAAAACACATCAAATAAGCCTTGAGGCATCGACACTGCATCAACAATTCAGATTTCTTGTATCATTACATCAATAATTGTTTCA
5000

CaFo     ::::::::::::::::::::A:::::::::::::::::::::::::::::::::::::::::::::::::::::::G:::::::::::::::::::::::::::::::::::::::::::::::::::::::::::::G:::::::::::::::::::::::::::::::::::::::::::::
Ca61R    ::::::::::::::::::::G:::::::::::::::::::::::::::::::::::::::::::::::::::::::A:::::::::::::::::::::::::::::::::::::::::::::::::::::::::::::C:::::::::::::::::::::::::::::::::::::::::::::
P1023-F3 ATCTATTAATTTCATGATCTGATAAGACTCATTTAGATCAAAGATGATATCAACTTCTACGTTAGGTGATTTCAGAATCTTCAGTAGAGGTTAGTTAGAATTTTCATTAGCATCTAAAATTTTGTCTAAGAGAAGCTTCATACGCTGTAACTCACGCTCTTAGATTACAAAATCTTCCGAGTCAACTTTTTTGAGGTAAT
5200

CaFo     ::::::::::::::::::::::::::::::::::::::::::::::::::::::::::::::::::::::::C:::::::::::::::::::::::::::G:::::::::::::::::::::::::::::::::A::::::::::::::::::::::::::::::C::T:::::::::::::::::::::::::::::::
Ca61R    ::::::::::::::::::::::::::::::::::::::::::::::::::::::::::::::::::::::::T:::::::::::::::::::::::::::A:::::::::::::::::::::::::::::::::G::::::::::::::::::::::::::::::T::C:::::::::::::::::::::::::::::::
P1023-F3 TAAATATTATATTAGTGAAACGTTCCACTTGAAGAAATTTTGGGATAGGAATATAATCGTAAAACATTGTCATCTAAAACATTAACGGTGAGGTTTGTCAATAACATTTTGCAGGTGAGGTTTTTTTTTGGAGGGAAAAAATAGTGTGATAGAAAGGTGATTGATTGCTGTTAAAAAAATTAACGGTGTTAGGATTATG
5400
```

```
CaFo      :::::::::::::::::::::::::::::::::::::::::::::::::::::::::::::::::::::::::::::::A::::::::::::::::::::::::::::::::::::::::::::::::::::::::::::::T:::::::::::::::::::::::::::::::::::::::::::::::::::::::::
Ca61R     :::::::::::::::::::::::::::::::::::::::::::::::::::::::::::::::::::::::::::::::G::::::::::::::::::::::::::::::::::::::::::::::::::::::::::::::A:::::::::::::::::::::::::::::::::::::::::::::::::::::::::
P1023-F3  ACTAAAAATACACCACTTAACTAAATAAAAAGGACTATTAGTGCTTTAGTTGAAAGAATATGTATTATTTTGTGTTTAAGACAAAAATAATGTATGATTTTGGGCCATTACTCTAATCCAACTAGTAAACATGAGATATTTAAAGTGTTAGTGAGAAAAGATACTTCAGTATTAGCAATGCAGGTCTTTAGGGAACTCGC
5600
```

|--> $bs6^{61}n$

```
CaFo      ::::::::::::::::::::::::::::::::::::::::::::::::::::::::::::::::::::::::::::::::::::::::-::::::::::::::::::::::::::::::::::::::::::::::::::::C::::::::::::::::::::::::::::::::::::::::::::::::::::::::::
Ca61R     ::::::::::::::::::::::::::::::::::::::::::::::::::::::::::::::::::::::::::::::::::::::::A::::::::::::::::::::::::::::::::::::::::::::::::::::T::::::::::::::::::::::::::::::::::::::::::::::::::::::::::
P1023-F3  AAGGAAGAAACAATCATCATCCTCTGCCTCTGCAGAAACGCTGAAGAAAGAGCATGATTATTATTTAGAGAATGGAAGTGCAGTACTAAGAGGAGCTTCTTGCTTTATGCGATGGAAATTGCCGAATTCCCATCCGTTACTTCACTGCCACTGAGATAGAAAAAGCAATAAAGCACCCTGAAAACAAAATGGAGCTTTCC
5800
```

$bs6^{61}n$-->|

```
CaFo      ::::::::::::::::::::::::::::::::::::::::::::::::::::::::::::::::::::::::::::::::::::::::::::::::::::::::::::::::::::::::::::::::::::::::::::::::::::::::::::::::::::::::::::::::::::::::::::::::::::::::
Ca61R     ::::::::::::::::::::::::::::::::::::::::::::::::::::::::::::::::::::::::::::::::::::::::::::::::::::::::::::::::::::::::::::::::::::::::::::::::::::::::::::::::::::::::::::::::::::::::::::::::::::::::
P1023-F3  TATGGGTATATGGTTACAGGATCACTGGACAAACGCCTCGTTTTCATTAGGATTTTCCCCGGATATTATAAAAATTACTTTTAATATTTTTCGAGATGTAGCAATTACTGCTCAGATGAGCCATCTCAAAAATGTACTGAGACTTGTCGGTTGCTGTCTAGAATTCGAAGAACCAGTTATAGTGTATGAATATGTTGAGG
6000
```

|--> $bs6^{61}pk$ |----- pr1638F ----->

```
CaFo      ::::::::::::::::::::::::::::::::::::::::::::::::::::::::::::::::::::::::::::::::::::::::::::::::::::::::::::::::::::::::::::::::::::::::::::::::::::::::::::::::::::::::::::::::::::::::::::::::::::::::
Ca61R     ::::::::::::::::::::::::::::::::::::::::::::::::::::::::::::::::::::::::::::::::::::::::::::::::::::::::::::::::::::::::::::::::::::::::::::::::::::::::::::::::::::::::::::::::::::::::::::::::::::::::
P1023-F3  CTATAAGTCTTTATTCTTTACTTTTTGAAAAGGGGACATATGATGATCAAATTAGAAAATCATTACTATCTTGGGGAAGTAGACTACGTATAGCCCACCAGGTCGTTTCTGCAGTCGTTTTTCTCCATACCGAATTTACTACGCCCGTCATCCATCGAGATCTGAAGCCATCGAATGTGATTGTAGATCAGAACAGCGGC
6200
```

```
CaFo      :::::::::::::::::::::::::::::::::::::::::::::::::::::::::::::::::::::::::::::::::::::::::::::::::::::::::::::::T::::::::::::::::::::::::::::::::::::::::::::::::::::::::::::::::::::::::
Ca61R     :::::::::::::::::::::::::::::::::::::::::::::::::::::::::::::::::::::::::::::::::::::::::::::::::::::::::::::G::::::::::::::::::::::::::::::::::::::::::::::::
::::::::::::::::::::::::::::::::::::::::
P1023-F3  GTCGCTAAACTATTGAATTTCTCATTATCTGTATCATTGCCCCGGGGAGAATTGGAAGTAGTAAAAGATGTTATGTGCGGAACGTATGGGTACTTAGCTTCAGAATATGCATCGTCTTGTATTGTTACTCAAAAAATAGACGTCTATAGCTTCGGAGTTGTCTCCTTCAGCTATTAACTGGATGGAAAATGAGTACTCTA
6400
```

←----- pr1638R ------|

```
CaFo      ::::::::::::::::::::::::::::::::::::::::::::::::::::::::::::::::::::::::::::::::::::::::::::::::::::::::::::::::::::::::::::::::::::::::::::::::::::::::::::::::::::::::::::::::::::::::::::::::::::::::
Ca61R     ::::::::::::::::::::::::::::::::::::::::::::::::::::::::::::::::::::::::::::::::::::::::::::::::::::::::::::::::::::::::::::::::::::::::::::::::::::::::::::::::::::::::::::::::::::::::::::::::::::::::
P1023-F3  CATTTAAAAGATCGCAATATGGACTACATAGTGGATCCTTCGGATTCAGATTTTAAGATGATGAACATTGAAGAGATTCATGTAATGGACATAGCAGATCCGGCCATTTTGGAAGAACATGGAATTGCGATTCAACAACAACTGAAAGATTGCTGGGATCTTGTTAACAAATGCACAAAGTCAAGCGGAAAAGATAGACC
6600
```

```
CaFo      :::::::::::::::::::::::::::::::::::::::::::::::::::::::::::::::::::::C:::::::::::::::::::::::::::::::::::::::::A::::::::::::::::::::::::::::::::::::::::::::::::::::::::::::- ::::::::::::::::::::::::::
Ca61R     :::::::::::::::::::::::::::::::::::::::::::::::::::::::::::::::::::::G:::::::::::::::::::::::::::::::::::::::::G::::::::::::::::::::::::::::::::::::::::::::::::::::::::::::A:::::::::::::::::::::::::::
P1023-F3  ATACATGATTGAAGTTGCAAGGGAATTACACCGAATTCACAAGTGCTTCCGTGCATGCCCTTAGTCTTGCCCACAATTAGTACAATAAGATGGCCAAAAAAAAAAAAATCAAGTGCACAAGAATTAAGCTTAGCTTTATTTGTGCGGTCATATTATGTCGGATAGTAAAAAA-GATCAGCACATAATGAAATTGTCAATT
6800
```

$bs6^{61}pk$ -->|1-->|

```
CaFo      :::::::::-::::::::::::::::::T::::::::::::::::::::::::::::::T:::::::::::::::::::::::::::::::::::::::::::::::::::::::::::::::::::::::::ATCATTAA::::T::::C:::::::::::::::::::::::::::::::::::::::::::::::::
Ca61R     :::::::::T::::::::::::::::::C::::::::::::::::::::::::::::::C:::::::::::::::::::::::::::::::::::::::::::::::::::::::::::::::::::::::::--------::::C::::T:::::::::::::::::::::::::::::::::::::::::::::::::
P1023-F3  :::::::::-AATTTGCGACATCATTTTCATTTTTAACTTGGCTCATATATGAAAAATATAATTTTCTTTTTATTTTTTCATTTTAACAAATCAATAGAGATTTAATTTATTATTTTATATTAATATTACTCTATCATTAAGTAATTATTCATAGGATTTTCAAAGAAAAATTTAATAAAACAACCCCTAAAGTGTCAAA
7000
```

```
CaFo      :::::::::::::::::A::::::::::::::::::::::::::::::::::::::::::::::T::::::::::::::::::::::::::::::::::::::::::::::::::::::::::::::::::T:::::::::::::T:::::::::::::C:::::::::TTTTCATCAAAAATA::::::::::::::::
Ca61R     :::::::::::::::::C::::::::::::::::::::::::::::::::::::::::::::::A::::::::::::::::::::::::::::::::::::::::::::::::::::::::::::::::::C:::::::::::::-:::::::::::::A:::::::::---------------::::::::::::::::
P1023-F3  TCAATAATTACTAAGTAAAAATTTGTACGGTGGCAACGAAAGCGACGGCCGAAATATTGAGAAGTGACGCTAGAGCAAATGCAAACTTCCTTATAGAGCGGATCTTTTGTCAACTTGAGTGGAATTAACGCTGTGTTTGGTATGATGAAAAATATTTTCCTGAAAAATATTTTCATCAAAAATAAATTGATTATATTTGA
7200
```

```
CaFo      ::::::::::::::::::::::C:::::::::T:::::::::::::::::::::::::::::G::::::::::::::::::::::::::G::::::::::::::::::::::::::::::::::::::::::::::::::::::::::::::::::::::::::::::::::::::::::::G:::::::::::::::::::::::::
Ca61R     ::::::::::::::::::::::T:::::::::C:::::::::::::::::::::::::::::T::::::::::::::::::::::::::A::::::::::::::::::::::::::::::::::::::::::::::::::::::::::::::::::::::::::::::::::::::::::::A:::::::::::::::::::::::::
P1023-F3  TTGGTGAGTAAAAAATATTTTTCAAAAAATAGTGTTTGTTTGGTAAATAGAAAATATATTTTGTAGAAAAATATCTTTTAGTGTTGATTGATGAACAAACAAAATATTATTTGAAAAATACTACTAAATTGTTAACTAACATATCATTGCCTCTAATAACTCAACAATTTGTAGGAACTAATTTAAGTATAGCAATCAAT
7400
```

```
CaFo      :::::::::A:::::::::::::::::::::::::::::::::::::::::::::::::::::::::::::::::::::::::::::::::::::::::::::::::::::::::::::::::::::::::::::::::::::::::::::G::::::::::::::::::::::::::::::::::::::
Ca61R     :::::::::G:::::::::::::::::::::::::::::::::::::::::::::::::::::::::::::::::::::::::::::::::::::::::::::::::::::::::::::::::::::::::::::::::::::::::::::A::::::::::::::::::::::::::::::::::::::
P1023-F3  ACGAATATTATGTACCAAAATATTACAAGTCAATAAAATTAAAGCAACTACTAATTATATAATCCAAGTTTCCTAACAATATTCCCTAAAATTAAGGGTGTTAAGTGGGCCGGCCGGGTCAACTCGGAATCGTCCTGTTAAGTTTAGCTGGGTTGTGGGCTGGGCTGGATCGGGCCGGGTTGTAGTTAAGTTGGCCGGGT
7600
```

```
CaFo      :::::::::::::::::::::::::::::::::::::::::::::::::::::::::::::::
Ca61R     :::::::::::::::::::::::::::::::::::::::::::::::::::::::::::::::
P1023-F3  CGGGTCCGGGTTCAACAGTAATAAAAATAACTCTGAATCGGCCCACTTAGCCCAACCCAGTCT
7663
```

<---- prBs6A_R ----|
