# Supplementary material for: Map-based cloning of the susceptibility locus conditioning bs6 resistance in pepper (Capsicum annuum L.) encoding mutant alleles of pseudokinase proteins
Source: Theor Appl Genet. 2026 Oct 20;139(11):308. doi: 10.1007/s00122-026-05407-2 (PMC13683501; doi:10.1007/s00122-026-05407-2)
Supplement: Supplementary file 9 [file 122_2026_5407_MOESM1_ESM.docx]

**Figure and Table legends**

Figure S1. Genetic mapping of 16 randomly selected F2 plants (from P1 to P16 of which 7 and 9 were *X. euvesicatoria* R6 susceptible and resistant, respectively). Susceptible and resistant phenotypes were determined as described in Materials and Methods. Molecular genetic markers were selected from publications as shown in Table S1. Polymorphism of the molecular markers (amplicon length) were converted to genotypes based on the parental pattern. In the figure, genotypes were represented by numbers (in colored background) as follows: 1 (yellow), paternal homozygotes; 2 (dark green), heterozygotes; 3 (purple), maternal homozygotes; 5 (light green), non-maternal homozygotes. XeR6 phenotypes (XeR6^R^/XeR6^S^) number 5 in pale green background, susceptible; number 3 in purple background, resistant phenotype. Genetic mapping was done by colormapping as described by Kiss et al. 1998. Bold horizontal bars in the colormap highlight recombination sites, which determined the location of the *bs6^61^* locus on LG6. Mb, megabase (pairs); CaPe, *C. annuum* var. Perennial; The positions of the markers were determined from the ASM2707371v1 genomic assembly of CaPe. Markers on LG6 are highlighted by blue box.

Figure S2. Genetic mapping of 92 F2 individuals (including those 16 plants used previously, see Fig. S1). Susceptible and resistant phenotypes were determined as described in Materials and Methods. Polymorphic molecular genetic markers were collected from published sequences (*C. annuum* 61R and *C. annuum* ECW specific RNASeq reads; (https://www.ncbi.nlm.nih.gov /sra/?term=capsicum));. Polymorphism of the molecular markers (amplicon length) were converted to genotypes based on the parental pattern. In the figure, genotypes were represented by numbers (in colored background) as follows: 1 (yellow), paternal homozygotes; 2 (dark green), heterozygotes; 3 (purple), maternal homozygotes; 5 (light green), non-maternal homozygotes. Genetic mapping was done by colormapping (Kiss et al. 1998). Bold horizontal bars in the colormap highlight the recombination sites that determined the location of the *bs6^61^ locus* between markers m1636 and m1652 on LG6. Mb, megabase (pairs); CaPe, *C. annuum* var. Perennial; The positions of the markers were determined from the ASM2707371v1 genomic assembly of CaPe. XeR6 phenotypes: number 5 in pale green background, susceptible; number 3 in purple background, resistant phenotype.

Figure S3. DNA and deduced amino acid sequence of the Bs6^Fo^A region. The nucleotide sequence of the Bs6^Fo^A region is shown. Transcribed and predicted protein coding regions are highlighted. The transcribed regions were identified by NCBI BLAST-search of CaECW SRA RNA-Seq reads (NCBI SRA databank; SRX24240619-SRX24240625, SRX24240631 and SRX24240642-SRX24240648) using the sequence of the Bs6^Fo^A regions, respectively, as query. Identified full length reads of 100% identity were aligned to the query sequence using the SeqMan program (Lasergene programs, data not shown). Overlapping reads were aligned to three regions: transcribed region *trBs6^Fo^1*; transcribed region *trBs6^Fo^NPK*, and transcribed region *trBs6^Fo^N-Bs6^Fo^PK*. Untranscribed sequences are shown in lower case letters, while transcribed regions are in capital letters. Predicted protein coding regions are underlined. Potential protein coding regions were identified by blastx search of „Non-redundant protein sequences” database of NCBI using the Bs6^Fo^A sequence as query, and *C. annuum* was given as selected „Organism”. That protein was selected for which the correspondence between the nucleic acid and amino acid sequence was 100%. For the *trBs6^Fo^1*, no protein product could be identified by blastx. For *trBs6^Fo^NPK* and *trBs6^Fo^N-Bs6^Fo^PK* one protein (denoted pBs6^Fo^NPK) and two proteins (denoted pBs6^Fo^N and pBs6^Fo^PK, respectively), were identified (see text and Fig. 3). The one letter amino acid sequence of the deduced proteins are given under the DNA sequence. Start (ATG) and stop codons (TAG/TAA) are highlighted by bold red letters. *, nonsense amino acids. Primers (pr1637-F and prBs6A-R) delimiting the CaFo-Bs6A region are shown below the sequence by dotted arrows.

Figure S4. DNA and deduced amino acid sequence of the bs6^61^A region. The nucleotide sequence of the bs6^61^A region is shown highlighting its transcribed and predicted protein coding regions. The transcribed regions were identified by NCBI BLAST-search of Ca61R SRA RNA-Seq reads (SRX30438917) using the sequence of the bs6^61^A region as query. Identified full length reads of 100 % identity were aligned to the query sequence using the SeqMan program (Lasergene programs, data not shown). Overlapping reads were aligned to three regions: transcribed region *trbs6^61^1*; transcribed region *trbs6^61^npk*, and transcribed region *trbs6^61^n-bs6^61^pk*. Untranscribed sequences are shown in lower case letters, while transcribed regions are in capital letters. Predicted protein coding regions are underlined. Potential protein coding regions were taken from Fig. S3. The three proteins in bs6^61^A region were denoted pbs6^61^npk, pbs6^61^n and pbs6^61^pk, respectively (see text and Fig 3.), were identified (see text and Fig. 3). The one letter amino acid sequence of the deduced proteins are given under the DNA sequence. Start (**ATG**) and stop codons (**TAG/TAA**) are highlighted by bold red letters. *, nonsense amino acids. Primers (prBs6A-R and pr1637-F) delimiting the bs6^61^A region are shown below the sequence by dotted arrows. One nucleotide insertions (T and A) and a T to G mutations are highlighthed by bold red character.

Figure S5. Detection of gene specfic transcripts in the VIGS experiment on CaFo plants silenced with non-specific sequences. Specific transcripts detected by *GADPH2* (Panel A), *Bs6^Fo^NPK* (Panel B), *Bs6^Fo^PK* (Panel C), *Bs6^Fo^N* (Panel D) gene specific primers; Samples: DNA ladder (1, 20, 27), CaFo (2, 8, 14, 21), CaFo plants silences with TRV::Bs6^Fo^NPK_VNs (3, 4, 9, 10, 15, 16, 22, 23), CaFo plants silenced with TRV::Bs6^Fo^PK_VNs (5,6, 11, 12, 17, 18, 24, 25), NTC (7, 13, 19, 26).

Figure S6. The nucleotide sequence of the recombination region of plant P164-F3 between primers pr1641F and pr1641R aligned to the sequence of the same region from parents *C. annuum* cv. Feherozon and *C. annuum* var. 61R, respectively. The nucleotide sequence of P164-F3 is shown on the bottom line. The two flanking primers pr1641F and 1641R are shown below the sequence. Those nucleotides which match in the parental sequence with that of P164-F3 are highlighted by „:”. Dissimilar nucleotides are highlighted with yellow (CaFo specific) and purple (Ca61R specific) background, respectively. Indels are shown by „-„. Grey background highlight the recombination region where the three sequence are identical. Note, that the flanking nucleotides of the grey sector in P164-F3 change genotypes indicating that the recombination event occurred in this sector. Yellow and purple background of the name of the sequence at the beginning of the sequence of each line represent CaFo and Ca61R specific genotypes/alleles.

Figure S7. The nucleotide sequence of the recombination region of plant P1023-F3 between primers m1637F-prBs6A_R aligned to the sequence of the same region from parents *C. annuum* cv. Feherozon and *C. annuum* var. 61R, respectively. The nucleotide sequence of P1023-F3 is shown on the bottom line. The two flanking primers pr1637F and prBs6A_R are shown below the sequence. Those nucleotides which match in the parental sequence with that of P1023-F3 is highlighted by „:”.Dissimilar nucleotides are highlighted with yellow (CaFo specific) and purple (Ca61R specific) background, respectively. Indels are shown by „-„. Grey background highlight the recombination region where the three sequence are identical. Note, that the flanking nucleotides of the grey sector in P1023-F3 change genotypes indicating that the recombination event occurred in this sector. Yellow and purple background of the name of the sequence at the beginning of the sequence of each line represent CaFo and Ca61R specific genotypes/alleles.

Figure S8. Results of transient expression assays in tobacco protoplasts. Panel A, p35S:*DsRed* control construct; Panel B, *Bs6^Fo^N*-*DsRed*-fusion constructs. The fluorescence signal was observed 48-72 h after infiltrating *N. benthamiana* leaves. Scale bars: 100 µm. All constructs, which were cloned in binary Ti-vectors, were flanked by a left border (LB) and a right border (RB) of T-DNA. *DsRED* was cloned into pCAMBIA 0390 vector under the control of CaMV35S promoter (35S-pr). The cassette of *Bs6^Fo^N* gene including its own promoter was cloned into pCAMBIA 0390 binary vector together with a 21 nt stretch from the 5’ end of the *Bs6^Fo^PK* gene (shown by „x” in the construct). The restriction enzyme cleavage sites used to make the constructs are shown. The primers used for the construction are shown in Table S1.

Table 1. Summary of the genotypes and phenotypes of the Virus Induced Gene Silencing and transient expression experiments (S - indicates susceptible phenotype, R – indicates resistant phenotype).

Table S1. Primer sequences used for genetic mapping, cloning different constructs and qPCR tests.

Table S2. Fragment sequences used in the constructs for VIGS and transient expression studies
